# Two new families of the FtsZ-tubulin protein superfamily implicated in membrane remodeling in diverse bacteria and archaea

**DOI:** 10.1186/1745-6150-5-33

**Published:** 2010-05-07

**Authors:** Kira S Makarova, Eugene V Koonin

**Affiliations:** 1 National Center for Biotechnology Information, NLM, National Institutes of Health Bethesda, Maryland 20894, USA

## Abstract

Several recent discoveries reveal unexpected versatility of the bacterial and archaeal cytoskeleton systems that are involved in cell division and other processes based on membrane remodeling. Here we apply methods for distant protein sequence similarity detection, phylogenetic approaches, and genome context analysis to described two previously unnoticed families of the FtsZ-tubulin superfamily. One of these families is limited in its spread to Proteobacteria whereas the other is represented in diverse bacteria and archaea, and might be the key component of a novel, multicomponent membrane remodeling system that also includes a Von Willebrand A domain-containing protein, a distinct GTPase and membrane transport proteins of the OmpA family.

This article was reviewed by Purificación López-García and Gáspár Jékely; for complete reviews, see the Reviewers Reports section.

## Findings

Proteins of the actin and tubulin superfamilies are major components of the cytoskeleton in all 3 domains of life, archaea, bacteria and eukaryotes [1,2]. FtsZ, the prokaryotic homolog of the eukaryotic cytoskeletal protein tubulin, is an essential protein that plays a central role in cell division of most bacteria and archaea [2-4]. Both FtsZ and tubulin undergo GTP- hydrolysis-dependent cycles of polymerization and depolymerization. Structural and biochemical analysis of both FtsZ and tubulin revealed mechanisms and structural determinants of GTP-binding and polymerization [5-7]. Recent progress in genome sequencing revealed numerous FtsZ paralogs, especially in archaea; some of these proteins lack the sequence motifs known to be important for the FtsZ-tubulin function, so their biological roles in the respective organisms remain obscure [8]. Furthermore, highly diverged FtsZ homologs were discovered on plasmids in several *Bacillus *species. These proteins, RepX from the pXO1 plasmid of *Bacillus anthracis *and TubZ from the *Bacillus thuringiensis *virulence plasmid pBtoxis, are required for plasmid replication and stability [9,10]. The diversity and functional flexibility of the FtsZ/tubulin superfamily suggests that additional families might surface when more genomic sequences become available. Here we report two new FtsZ-like protein families found in archaea and bacteria. One of these families is associated with a set of genes that can be predicted to encode multiple components of a novel molecular machinery for membrane remodeling.

### Identification of new FtsZ-tubulin families

Analysis of the gene context of cell division related genes in archaea, including serine/threonine protein kinases (PK) implicated in FtsZ phosphorylation, revealed a locus in *Halorhabdus utahensis *(Huta_2050-Huta_2056) that, along with 3 PK genes, encompassed 3 additional "hypothetical genes" which we further examined using distant sequence similarity search methods. For the 1051 amino acid long Huta_2051 protein, HHpred search [11] identified a statistically significant similarity (E-value = 1.4 × 10^-5^) with the FtsZ protein from the hyperthemophilic bacterium *Thermotoga maritima *(PDB:d1w5). The region of similarity encompassed the N-terminal portions of these proteins and included several structural elements of the nucleotide-binding core domain, in particular, the signature GTP-binding loop containing the GGGTG(S/T)G motif [8,12]. The central portion of the Huta_2051 protein (445-781 aa) consists of an extended coiled-coil as predicted using the Marcoil program [13], followed by a mostly α-helical globular domain according to the secondary structure prediction made using the PSIPRED program [14].

We performed an exhaustive PSI-BLAST search [15,16] using the sequence of the N-terminal FtsZ-like domain of Huta_2051 as the initial query to identify all representatives of the same protein family. In this search, we detected 8 proteins from archaea and 113 proteins from bacteria, none of which has been previously annotated as FtsZ-like (Additional file 1). The majority of these proteins have the same organization as the Huta_2051 protein, namely, an N-terminal FtsZ-like domain, an extended coiled-coil region, and a mostly α-helical C-terminal domain. Notably, 4 FtsZ-like proteins from Planctomycetes possess an additional N-terminal PK domain.

One of the previously unnoticed archaeal FtsZ-like proteins was detected in two closely related strains of the crenarchaeaon *Sulfolobus solfataricus *(SSO1376). This is the first observation of FtsZ/tubulin proteins in Crenarchaeota which apparently employ a distinct mechanism of cell division centered around homologs of the eukaryotic ESCRT-III complex proteins [17-20]. The gene encoding this protein in *S. solfataricus *is disrupted by a transposon, most likely, as a result of a relatively recent event, given that the protein sequence still retains the GTP-binding motifs in the N-terminal domain. Consequently, this sequence could not be included in the phylogenetic analysis (see below). Nevertheless, this protein clearly is quite diverged from other archaeal FtsZ sequences and might be also functionally distinct given the presence of transmembrane helices at the N-terminus. It seems likely that this protein is first discovered representative of a crenarchaea-specific subfamily of FtsZ-like proteins, additional members of which are likely to be detected when more crenarchaeal genomes are sequenced.

We further applied motif search, namely, searched for the signature GTP-binding loop sequence GGGTG(S/T)G of the FtsZ-tubulin superfamily in the Refseq database in an attempt to identify additional protein families containing the FtsZ domain. In addition to the FtsZ families described previously [8,9] and the family reported above, we detected another previously unknown family. This family can be typified by the PFL_1302 protein from *Pseudomonas fluorescens *Pf-5 and is encoded in the genomes of several other Proteobacteria (Additional file 1). For PFL_1302, HHpred search identifies statistically significant similarity (E-value = 0.0033) with the profile for PF00091 which includes the GTPase domain of the Tubulin-FtsZ family, revealing conservation of all structural elements of the nucleotide-binding core domain [12]. Thus, we identified two previously unnoticed families of FtsZ-like proteins; the first, widespread family was denoted FtsZ-like 1 (FtsZl1) and the second, proteobacterial family FtsZ-like 2 (FtsZl2). Unlike the FtsZl1 family, the FtsZl2 proteins do not contain a coiled-coil domain but instead possess a ~300 aa C-terminal region with a predicted mixed α/β structure for which no similarity with other protein families was detected.

**Figure 1:**
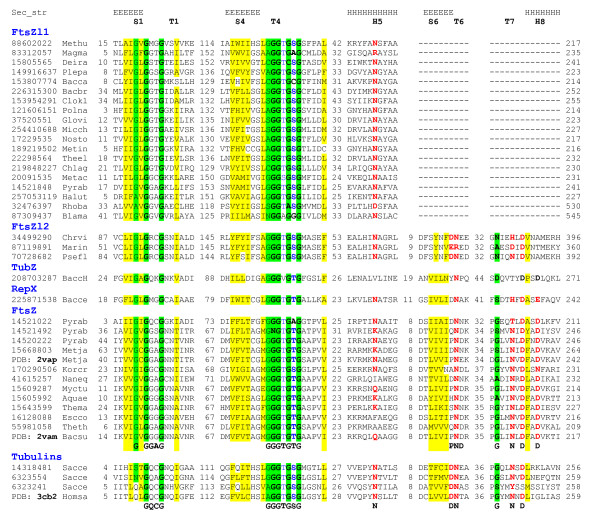
**Multiple alignment of conserved blocks of all previously described and the two new protein families of the FtsZ-tubulin superfamily**. The sequences are denoted by their GI numbers and species names. The positions of the first and the last residues of the aligned region in the corresponding protein are indicated for each sequence. Key elements of secondary structure based on known FtsZ tertiary structures are shown on two lines above the alignment [6,12,44]. The evolutionarily conserved functional motifs of the FtsZ and tubulin families are shown underneath the alignment for the corresponding groups [8,9]. The alignment columns are colored on the basis of the respective position conservation throughout the superfamily: yellow background indicates hydrophobic residues (ACFILMVWY), red letters indicate polar residues (DEHKNQR), blue letters indicate hydroxyl-containing residues (TS), and green background indicates small residues (ACGNPSTV). Species abbreviations: Methu - *Methanospirillum hungatei *JF-1; Magma - *Magnetospirillum magneticum *AMB-1; Deira - *Deinococcus radiodurans *R1; Plepa - *Plesiocystis pacifica *SIR-1; Bacca - *Bacteroides caccae *ATCC 43185; Bacbr - *Brevibacillus brevis *NBRC 100599; Clokl - *Clostridium kluyveri *DSM 555; Polna - *Polaromonas naphthalenivorans*; Glovi - *Gloeobacter violaceus *PCC 7421; Micch - *Microcoleus chthonoplastes*; Nosto - *Nostoc sp*. PCC 7120; Metin - *Methylacidiphilum infernorum *V4; Theel - *Thermosynechococcus elongatus *BP-1; Chlag - *Chloroflexus aggregans *DSM 9485; Metac - *Methanosarcina acetivorans *C2A; Pyrab - *Pyrococcus abyssi *GE5; Halut - *Halorhabdus utahensis *DSM 12940; Rhoba - *Rhodopirellula baltica*; Blama - *Blastopirellula marina *DSM 3645; Chrvi - *Chromobacterium violaceum*; Marin - *Marinomonas sp*.; Psefl - *Pseudomonas fluorescens*; BaccH - *Bacillus cereus *H3081-97; Bacce - *Bacillus cereus *03BB102; Metja - *Methanocaldococcus jannaschii*; Korcr *- Korarchaeum cryptofilum *OPF8; Naneq - *Nanoarchaeum equitans *Kin4-M; Myctu - *Mycobacterium tuberculosis *H37Rv; Aquae - *Aquifex aeolicus *VF5; Thema - *Thermotoga maritima *MSB8; Escco - *Escherichia coli *K-12; Theth - *Thermus thermophilus *HB8; Sacce - *Saccharomyces cerevisiae*.

To compare the two new families of FtsZ-like proteins with the previously described families, we examined in detail the multiple alignment of a representative set of sequences from FtsZ-tubulin families (Figure 1). Both new families possess several family-specific inserts in the region between the highly conserved structural elements located in loops T1 and T4 (see also the complete alignment in Additional file 2). The FtsZl2 family contains all conserved motifs of the nucleotide-binding domain including functionally important loops T1, T4, T6 and T7, and the connector helix H7, whereas in the FtsZl1 family, counterparts to the T6-H7 structural elements could not be confidently identified. In this case, the most distal structural element that could be reliably aligned is the H5 helix (Figure 1). Furthermore, the secondary structure prediction for the FtsZl1 family was not fully compatible with the known structure of the S6-T7 region of the nucleotide-binding and connector domains (Additional File 2). Along the same lines, we did not detect any similarity between the C-terminal domain of the FtsZl1 family and C-terminal domains of previously characterized FtsZ-tubulin superfamily proteins. These structural elements have been shown to contribute to the formation of FtsZ filaments [6]. Accordingly, the FtsZl1 proteins might form filaments via a novel mechanism or, less likely, might not form filaments at all. In general, the FtsZl2 and especially FtsZl1 families seem to include the most structurally diverged proteins in the entire FtsZ-tubulin superfamily and therefore are of particular interest for further structural and functional analysis.

### Phylogenetic analysis of the FtsZ-tubulin superfamily

**Figure 2:**
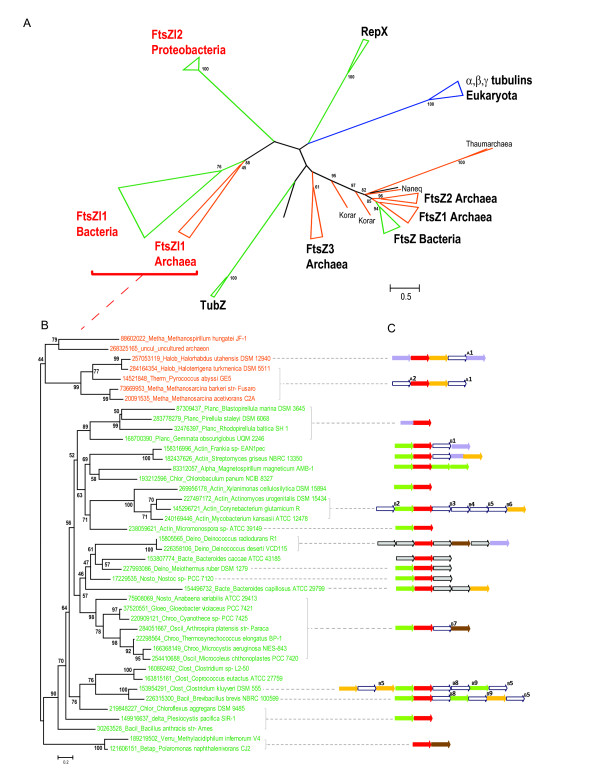
**Phylogentic analysis of the FtsZ-tubulin superfamily and representative operonic architectures of FtsZl1 family genes**. Color code: eukaryotes - blue; bacteria - green; archaea - orange. The RELL bootstrap values (%) are indicated for selected major branches. A. Phylogeny of the FtsZ-tubulin superfamily. The tree was reconstructed from 118 phylogenetically informative positions. B. Phylogeny of FtsZl1 family. The tree was reconstructed from 103 phylogenetically informative positions. C. Genomic context of the FtsZl1 family genes. Homologous genes are shown by arrows of the same color; genes are shown not to scale. Color code for the gene context: green arrows, VWA-domain-containing proteins; red, FtsZ-like 1 family proteins; brown, OmpA-like proteins; pale blue, serine/threonine protein kinases; yellow, GTPase; empty arrows (A1-A2; B1-B9) represent families that are associated with the FtsZ-like 1 genes in many genomes (see Additional File 6); gray arrows, genes associated with the FtsZ-like 1 family genes in one or a few related genomes.

To characterize the relationships between the new Ftzl1 and FtsZl2 families and the rest of the FtsZ-tubulin proteins, we employed the alignable blocks of the FtsZ-tubulin nucleotide-binding domain to construct a tree for a representative set of sequences from all families, with a special emphasis on FtsZl1 and FtsZl2 (103 sequences in total, including 41 from the FtsZl1 family and 10 from the FtsZl2 family) (Figure 2A). Generally, the tree topology reproduces all previously established major branches and relationships between them [8,9]. These include the monophyletic bacterial FtsZ and eukaryotic α,β,γ tubulin branches, 3 major archaeal clades, and distinct branches for the RepX and TubZ families, suggesting that the alignment of the nucleotide-binding domains contains sufficient information for phylogenetic inference.

The FtsZl1 and FtsZl2 families clearly form distinct branches; the respective branch lengths are comparable to those seen for the plasmid-born TubZ and RepZ family and eukaryotic tubulins (original tree is available in the Additional file 3). The second salient observation is that the archaeal branch of the FtsZl1 family is confidently separated from the bacterial branch. To further assess this observation, we constructed a separate FtsZl1 family tree using more sequences (45) (Figure 2B; the original tree is available in Additional file 4). Despite the formally low bootstrap values for the separation of the archaeal and bacterial branches, the tree includes a strongly supported (90%) branch that includes all bacterial sequences except for two sequences from *Polaromonas naphthalenivorans *and *Methylacidiphilum infernorum *V4. However, the neighborhoods of the FtsZl1 gene in these two bacteria resemble those in other bacteria, so there are no indications of horizontal gene transfer between archaea and bacteria within this family. The FtsZl1 family is represented in most of the major bacterial lineages, with the notable exception of the hyperthermophiles *Thermatogales *and *Aquificales*. The tree of the bacterial part of the Ftzl1 family shows likely case of both horizontal gene transfer and long term vertical inheritance. The vertical evolutionary pattern is particularly characteristic of the Planctomycetes, actinobacteria and cyanobacteria, all of which form distinct clades. Thus, despite the patchy distribution of the FtsZl1 family, the results of phylogenetic analysis suggest that this family evolved as a result of an ancient duplication that might even antedate the last universal common ancestor of cellular life forms.

### The genomic context of the new FtsZ-like families and functional implications

To shed light on the potential roles of the newly detected families of FtsZ-like proteins in archaea and bacteria and their functional associations with other proteins, we analyzed the genomic context of the respective genes. Given that the new FtsZ-like families are typically present in the respective genomes along with conventional FtsZ, it seems unlikely that the FtsZl1 or FtsZl2 are directly involved in cell division. The genomic context analysis revealed a putative distinct molecular machinery associated with the FtsZl1 family (Figure 2C and Additional file 5). The predicted operon organization generally correlates with the topology of the phylogenetic tree of the FtsZl1 proteins. The majority of the predicted operons, in addition to the FtsZl1 family gene, contain a gene for a protein containing a von Willebrand factor type A (vWA) domain. VWA domain-containing proteins have been studied primarily in eukaryotes and have been shown to contribute to a variety of cellular functions such as basal membrane formation, cell migration, cell differentiation, adhesion, signaling, and chromosomal stability [21]. Often, vWA-domains are fused or interact with other domains or proteins to form multiprotein complexes; these interactions typically depend on divalent cations [21]. Although many bacteria and some archaea encode vWA-domain proteins, specific information on their functions is scarce. The best characterized prokaryotic vWA-domain proteins also contain an AAA+ ATPase domain and function as Mg^2+ ^and Co^2+ ^chelatases that are involved in protoporphyrin IX biosynthesis; these chelatases are represented in bacteria, archaea and chloroplasts [22]. The vWA-domain proteins associated with the FtsZl1 family are highly diverse and are fused to various, mostly, uncharacterized domains. Some of these proteins contain predicted signal peptides and/or transmembrane segments, and could be secreted or membrane-associated. In the majority of these proteins, the metal-binding loops of the vWA domain (the so-called MIDAS motif, DxSxS [21]) are intact suggesting that conformational changes of these vWA domains are regulated by divalent cations.

Other frequent components of the predicted vWA/FtsZl1 operons are PKs and members of a diverged family of predicted GTPases; most of the latter are annotated as "hypothetical proteins" but contain clearly identifiable sequence signatures of P-loop GTPases including the Walker A and B motifs, and the TxKD signature GTPase motif [23]. In bacteria, in contrast to archaea, these GTPases typically are not membrane-associated but in some cases are fused to other domains including a domain related to the cysteine-rich domain [24] of the chaperone protein DnaJ which is involved in protein translocation through membranes [25].

In several genomes, the FtsZl1 genes are associated with proteins containing OmpA family peptidoglycan-binding domains that are known to interact with both the inner and the outer membrane of Proteobacteria (Figure 2C). These proteins form 8-14 stranded transmembrane β-barrel domains and function as porins, adhesins or enzymes such as the PagP acyltransferase [26-28].

Most of the other genes in the predicted operons containing FtsZl1 genes are lineage-specific and do not encode any recognizable domains (Additional File 6). Many of these proteins contain predicted transmembrane regions suggesting that the respective multisubunit complexes are associated with membranes. In general, the genomic contexts of the FtsZl1 genes suggest that these proteins are central components of novel, uncharacterized protein complexes involved in membrane remodeling and vesicle biogenesis.

In a sharp contrast to the FtsZl1 genes, genes encoding FtsZl2 family proteins are not associated with any other genes, so no functional clues could be obtained as to their likely functions.

## Concluding remarks

Several recent findings indicate that bacterial and archaeal cytoskeleton systems involved in cell division and various other processes that involve membrane remodeling are considerably more versatile than previously realized [1,2]. The most notable of these discoveries arguably are the demonstration that ESCRT-III-like complexes are responsible for cell division in the hyperthemophilic Crenarchaeota of the order *Sulfolobales *and probably *Desulfurococcales *[17,18,20]; the demonstration of the diversity of cytoskeleton systems in the Planctomycete-Verrucomicrobia-Chlamydia superphylum of bacteria [29]; and the detection of highly conserved homologs of the eukaryotic actins in the third crenarchaeal order, *Thermoproteale*s [30]. In addition, the formation of exosome-like vesicles during stress response and virus egress from infected cells has been reported in *Sulfolobales *as [31,32], and DNA-containing vesicles have been observed in some *Thermococci *[33]. The actin homolog MamK plays a role in the subcellular location of magnetosomes, membrane-bound organelles in the α-proteobacterium *Magnetospirillum madneticum *[34]. In another α-proteobacterium, *Caulobacter crescentus*, a lineage-specific protein, crescentine, is responsible for maintaining the distinct cell shape [35]. In Spirochaetes and Myxococci, intracellular filaments have been observed and implicated in cell motility [2]. In many cases, the proteins that are involved in the formation and functioning of these structures are unknown. Here we describe two FtsZ-like protein families which are candidates for involvement in some of these functions. In particular, the FtsZl1 family is most likely involved in a basic, ancient mechanism of membrane remodeling, which often requires GTP hydrolysis and is regulated by PK. The analysis presented here may trigger and facilitate experimental study of these processes.

## Methods

The NCBI Refseq database [36] was used for retrieval of information on genomic context. For partially sequenced genomes that are not represented in Refseq, the context information was retrieved from the original nucleotide GenBank files. Protein sequence database searches were performed using PSI-BLAST [37] with an inclusion threshold E-value of 0.01 and no composition-based statistical correction. In addition, distant similarity detection approaches were applied, namely, the conserved domain database (CDD) search [38] and the HHpred server that is based on the comparison of protein family profiles using the Hidden Markov Model technique [11]. Multiple alignments of protein sequences were constructed by using the Promals3D program [39], followed by a minimal manual correction on the basis of local alignments obtained using PSI-BLAST [37] and HHpred [11]. Protein secondary structure was predicted using the PSIPRED program [14]. Signal peptides were predicted using the SignalP program [40] and transmembrane regions were predicted using the TMHMM software [41].

Maximum likelihood (ML) phylogenetic trees were constructed by using the MOLPHY program [42] with the JTT substitution matrix to perform local rearrangement of an original Fitch tree [43]. The MOLPHY program was also used to compute RELL bootstrap values.

## Competing interests

The authors declare that they have no competing interests.

## Authors' contributions

KSM collected and analyzed data, and wrote the original draft of the manuscript; EVK analyzed data and wrote the final manuscript that was approved by both authors.

## Reviewer 1

Gáspár Jékely, Max Planck Institute for Developmental Biology, Tübingen

This interesting paper describes two novel families related to the filament forming FtsZ/tubulin proteins. The identification of these novel families and the careful analyses of the sequences already warrant publication. Besides, the authors also provide useful information about the genomic context of the novel FtsZ-like proteins and propose that they may be involved in membrane remodeling processes.

This is a very interesting possibility, but I think that it is very difficult to decide based on the data, and there are also alternative interpretations possible. Regarding the membrane remodeling complexes formed by OmpA-like proteins, von Willebrand factor type A domain proteins and FtsZ-like proteins, it is unclear in what cellular context these could function together. Looking at the genomic context data in their AF5 supplementary table, it is clear that many of the FtsZl1 genes, in gram-negatives at least, are associated with periplasmic and outer-membrane components. OmpA-like proteins are outer membrane proteins. Several of the proteins containing a von Willebrand factor type A domain have a signal peptide and are likely periplasmic (I only checked Corynebacterium glutamicum cgR_2627, Bacillus thuringiensis konkukian BT9727_3336 and Micromonospora sp. ATCC 39149 MCAG_00589, these all have a signal peptide). On the contrary, the FtsZl1 protein is likely cytoplasmic. Given the different subcellular localizations, which of the two membranes could be remodeled in Gram-negatives? A cytoplasmic FtsZ-like protein will not form a complex with periplasmic and outer-membrane proteins. Given this problem, it would be very useful to provide the predicted cellular localization (cytoplasm, outer membrane etc.) for all of the proteins listed in the supplementary table AF5 and Figure 2C.

### Authors' response

*The predictions of cellular localization for the proteins associated with the FtsZ homologs are given in *Additional File 6. *The emerging picture is extremely complicated and at present does not allow a detailed reconstruction of the macromolecular organization of the respective complexes. Given that all major predicted components of these complexes (vWA containing proteins, OmpA-like proteins, and many other proteins encoded in the respective operons; see *Additional File 6*) appear to be membrane-associated (obviously, the distinction between the inner and outer membrane is only relevant for Proteobacteria) and given the accumulating evidence of the existence of diverse membrane structures in prokaryotes (see text), we consider the membrane remodeling hypothesis to most plausible functional prediction for the FtsZl1 proteins. In Proteobacteria that encode FtsZl1, it is the inner membrane that might be remodeled with the participation of this protein*.

It would be very useful to see a discussion about whether these novel proteins are likely to form filaments, a property necessary for membrane remodeling activity. Could the additional C-terminal domains hinder filament formation? Besides, FtsZl1 lacks the T6-H7 structural elements. How does it fit to the known structure of the FtsZ filaments?

### Authors' response

*This is indeed an interesting and important question. As indicated in the text, FtsZl2 contains all structural elements characteristic of FtsZ-tubulin and can be confidently predicted to form filaments. The case of FtsZl1 is different as these proteins seem to lack some of structural elements required for filament formation. There are three potential explanations for this: i) these elements are present but are extremely diverged and not identifiable by our methods, ii) the FtsZl1 proteins employ a novel mechanism of filament formation, perhaps, mediated by their unique C-terminal structures, iii) the FtsZl1 proteins do not form filaments. A definitive choice between these possibilities will become feasible only as a result of experimental study of these proteins. Nevertheless, so far all characterized FtsZ-tubulin superfamily proteins have been shown to form filaments, so it seems likely to be their intrinsic property. We have a clarification on this issue to the text*.

Given these difficulties with the functional predictions, I would suggest to change the title and the abstract and to provide a more careful discussion of the possible function of these proteins.

### Authors' response

*We changed "predicted" to "might be" in the Abstract to soften the stance. Nevertheless, as pointed out in the response to the reviewer's comment above, we do consider membrane remodeling the most likely function of these proteins and accordingly were disinclined to modify the title. The interested reader will be duly forewarned of the possibility of alternative interpretations by the reviewer's comments*.

Glancing through the genomic context data, I would propose, as an alternative to the authors' interpretation, that FtsZl1 proteins may have a role in transmembrane transport and/or signalling processes. For example in *Polaromonas naphthalenivorans *the FtsZl1 protein is associated with Pnap_3259 (TRAP-type uncharacterized transport system protein, periplasmic component). This protein has a signal sequence and a domain similar to the periplasmic binding proteins (PBP), components of Tripartite ATP-independent periplasmic (TRAP) transporter systems. In *Pirellula staleyi *FtsZl1 is associated with Psta_0487, a diguanylate phosphodiesterase, involved in cyclic diguanylic acid (c-di-GMP) signalling, regulating e.g. motility, biofilm formation and virulence. In *Thermosynechococcus elongatus *FtsZl1 is associated with tll1019, a PilA-like protein, involved in the general secretion pathway for extracellular targeting of proteins from the periplasm. In *Bacteroides capillosus *FtsZl1 is associated with BACCAP_04120, an OEP family (Outer membrane efflux protein) with a signal peptide and periplasmic localization. In *Arthrospira platensis *FtsZl1 is associated with AplaP_010100009370, the periplasmic component of an ABC-type branched-chain amino acid transport system. There are also other examples that suggest a transport function.

### Authors' response

*Our hypothesis is based on a detailed analysis of the genomic context. However, we relied only on those components of the FtsZl1 neighborhoods that are conserved in at least a few genomes. We noticed the cases mentioned by the reviewer, and most of them are listed in *Additional File 5, *along with a few additional ones; however, these genes are represented in only one or several closely related organisms and therefore cannot be unequivocally implicated in functionally important interactions with FtsZl1*.

## Reviewer 2

Purificación López-García, Unité d'Ecologie, Systématique et Evolution - CNRS UMR8079 Université Paris-Sud

The authors of this manuscript unveil two previously undescribed families of FtsZ/tubulin superfamily that are widespread in prokaryotes and the Proteobacteria, respectively. The use of distant protein similarity detection, phylogenetic and genome context analyses allowed the detection of these new FtsZ-like families, FtsZl1 and FtsZl2. Interestingly, one of these families is present in Sulfolobus genomes, which were so far thought to lack FtsZ/tubulin homologs. The detection of these distant homologs to FtsZ/tubulins opens the way to their functional exploration and the evolution of cell division mechanisms.

The observation of FtsZ-like proteins in Crenarchaeota (Sulfolobus) is interesting. However, the corresponding homologs do not appear in the phylogenetic analysis of Figure 2. Are the proteins not alignable? It would be interesting to know whether this is a case of horizontal transfer or a vertically inherited homolog from the last common ancestor.

### Authors' response

*The N-terminal portion of the FtsZ-like protein sequence from Sulfolobus is perfectly alignable with other family members. However, there is a transposon insertion immediately following the GTP-binding loop, and the part of the SSO1376 sequence after the transposon is partly disrupted and poorly alignable (clearly, the protein was rather recently inactivated). The best hit for the part of the protein corresponding to the GTP-binding domain comes from an uncultured archaeon (GI: 268325165), so there is no indication of a bacterial transfer. However, this is the only archaeal FtsZl1 protein that is fused to a transmembrane region, so we believe that it has a distinct evolutionary history that hopefully will be elucidated when additional FtsZl1 sequences with this type of domain organization become available*.

## Supplementary Material

Additional file 1 **Complete list of proteins that belong to FtsZ-like 1 and FtsZ-like 2 families**. The data provided represent the list of all sequences that were found to belong FtsZl1 and FtsZ2 families. Click here for file

Additional file 2 **Multiple alignments of the FtsZ-tubulin superfamily proteins**. The provided alignments support the analysis and description of A. FtsZ superfamily nucleotide-binding domain; B. FtsZl1 family; C. FtsZl2 family. Click here for file

Additional file 3 **The Newick format tree of the FtsZ-tubulin superfamily and results of bootstrap analysis**. The provided Newick format tree allows one to view the tree presented on the Figure 2A using any tree-viewing program and to see the original RELL bootstrap analysis data. Click here for file

Additional file 4 **The Newick format tree of the FtsZl1 family and results of bootstrap analysis**. The provided Newick format tree allows one to view the tree presented on the Figure 2B using any tree-viewing program and to see the original RELL bootstrap analysis data. Click here for file

Additional file 5 **Genomic neighborhoods of the FtsZl1 and FtsZl2 genes for a representative set of archaea and bacteria**. The provided data give details for analysis shown in the Figure 2C. Click here for file

Additional file 6 **Uncharacterized putative components of the predicted FtsZl1-centered membrane remodeling system**. The data represents the list and comments for gene families associated with FtsZl1 genes in archaeal and bacterial genomes. Click here for file
