# Supplementary material for: Two new families of the FtsZ-tubulin protein superfamily implicated in membrane remodeling in diverse bacteria and archaea
Source: Biol Direct. 2010 May 7;5:33. doi: 10.1186/1745-6150-5-33 (PMC2875224; doi:10.1186/1745-6150-5-33)
Supplement: Additional file 2 — Multiple alignments of the FtsZ-tubulin superfamily proteins. The provided alignments support the analysis and description of A. FtsZ superfamily nucleotide-binding domain; B. FtsZl1 family; C. FtsZl2 family. [file 1745-6150-5-33-S2.DOCX]

All alignments were constructed by PROMALS-3D program. Amino acids within alignment are colored according to PSIPRED secondary structure prediction - red: alpha-helix, blue: beta-strand. The consensus predicted secondary structures are shown in the last line in each block. If the fraction of helix or strand predictions among representative sequences in a position is larger than 0.5, the consensus letter is "h" or "e", respectively. **Conserved** amino acid residues: bold and uppercase letters (such as **G**); **aliphatic** residues (**I, V, L**): l; **aromatic** residues (**Y, H, W, F**): @; **hydrophobic** residues (**W, F, Y, M, L, I, V, A, C, T, H**): h; **alcohol** residues (**S, T**): o; **polar** residues (**D, E, H, K, N, Q, R, S, T**): p; **tiny** residues (**A, G, C, S**): t; **small** residues (**A, G, C, S, V, N, D, T, P**): s; **bulky** residues (**E, F, I, K, L, M, Q, R, W, Y**): b; **positively charged** residues (**K, R, H**): +; **negatively charged** residues (**D, E**): -; **charged** (**D, E, K, R, H**): c

**A. FtsZ/tubulin superfamily**

Conservation: 55 9 9

gi_14318481 1 -----------------MREIIHISTGQCGNQIGAAFWETI--CGEHG---------------------L 30

gi_18568139 1 -----------------MREIVCVQAGQCGNQIGSKFWEVI--SDEHG---------------------V 30

gi_6323554 1 -----------------MREVISINVGQAGCQIGNACWELY--SLEHG---------------------I 30

gi_15241179 1 -----------------MREIISIHIGQAGIQVGNSCWELY--CLEHG---------------------I 30

gi_71397525 1 -----------------MREAICIHIGQAGCQVGNACWELF--CLEHG---------------------I 30

gi_6323241 1 ----------------MGGEIITLQAGQCGNHVGKFLWSQL--AKEHA---------------------I 31

gi_71652627 1 ----------------MPREIITLQAGQCGNQVGSEFWRLL--CAEHG---------------------I 31

gi_15233174 1 ----------------MPREIITLQVGQCGNQIGMEFWKQL--CLEHG---------------------I 31

gi_190016319 1 ----------------MPREIITLQLGQCGNQIGFEFWKQL--CAEHG---------------------I 31

gi_161528770 1 ----------------VKEPVLVVGLGGAGSKLALKAKDS------------------------------ 24

gi_118194654 1 ----------------MRGPVLLVGAGGAGSRLASRAGGI------------------------------ 24

gi_20089831 1 ----------------FLLNILIIGNGQCGNRILDSINRHA--LGG------------------------ 28

gi_14521022 1 ------------------MRAIIIGIGQCGGKIADIFSLVD----------------------------- 23

gi_240102830 1 ------------------MRAIIIGVGQCGTKIADLFSLVD----------------------------- 23

gi_15789554 1 ------------------MKAALIGVGQAGGKVTEALLAED----------------------------- 23

gi_257052276 1 ------------------MKVVLIGLGQAGGKLTQALASYD----------------------------- 23

gi_126178676 1 ------------------MRVFFIGFGQAGGKIVDMFIEQD----------------------------- 23

gi_11498814 1 ----------------ILMRFFIIGFGQAGGKILDMFIENE----------------------------- 25

gi_170290506 1 ----------------TEAKINIVGIGGCGNNIISAFYKKF----------------------------- 25

gi_182414456 1 ----------------RAIAIKMVGVGGAGSNAVDRLKMEN----------------------------- 25

gi_41615257 1 ----------------RAANIKVVGVGGAGCNIIEWLYKKK----------------------------- 25

gi_14521492 1 ----------------DLIKIAVIGVGGSGNNTITRLYDLG----------------------------- 25

gi_16082526 1 ----------------RNFRIKVFGFGGSGSNTINRLMREN----------------------------- 25

gi_170290956 1 ----------------VKARIVIMGVGGGGSNTITRLNAIG----------------------------- 25

gi_41614929 1 ----------------IKKKIKVIGVGGAGCNTINRLYELG----------------------------- 25

gi_11498178 1 ----------------ETPKIVVVGCGGSGNNTVHRLSNMN----------------------------- 25

gi_15668803 1 ----------------GEARIVVVGCGGAGNNTINRLMEIG----------------------------- 25

gi_15789500 1 ----------------GDPRIVIVGCGGAGNNTVNRLYNIG----------------------------- 25

gi_20092672 1 ----------------GQPRIMIVGCGGAGNNTVNRLYNIG----------------------------- 25

gi_126179395 1 ----------------GQPRIVIVGCGGAGNNTVNRLYHMQ----------------------------- 25

gi_15679670 1 ----------------SRAKIYVVGTGGAGNNTVTRLSEIG----------------------------- 25

gi_11498146 1 ----------------LKTVIKVIGVGGGGCNTITRMYEEG----------------------------- 25

gi_15789633 1 ----------------LQTNITVVGCGGAGSNTVDRMATEG----------------------------- 25

gi_126178359 1 ----------------LRTEIAVVGCGGGGSNTVTRMADEG----------------------------- 25

gi_20093060 1 ----------------LKTTIKVIGCGGGGSNSIQRMMGEG----------------------------- 25

gi_14520222 1 ----------------IKARIYVVGVGGAGCNTVNRMMEVG----------------------------- 25

gi_15668546 1 ----------------TKAKITVVGCGGAGNNTITRLKMEG----------------------------- 25

gi_53711593 1 ----------------SPKIIKVIGVGGGGGNAVNHMYREG----------------------------- 25

gi_170291053 1 ----------------VTGNLVIVGVGGCGSNTIDNISKLG----------------------------- 25

gi_15605992 1 ----------------NPCKIKVIGVGGGGSNAVNRMYEDG----------------------------- 25

gi_16128088 1 ----------------NDAVIKVIGVGGGGGNAVEHMVRER----------------------------- 25

gi_15643599 1 ----------------NNLKIKVIGVGGAGNNAINRMIEIG----------------------------- 25

gi_15639381 1 ----------------SPTVIKVIGAGGGGSNAVNRMMSCG----------------------------- 25

gi_55981058 1 ----------------EGAVIKVIGLGGAGNNAVNRMIEAG----------------------------- 25

gi_16330088 1 ----------------NIAKIKVIGVGGGGCNAVNRMIASG----------------------------- 25

gi_15609287 1 ----------------YLAVIKVVGIGGGGVNAVNRMIEQG----------------------------- 25

gi_255767353 1 ----------------GLASIKVIGVGGGGNNAVNRMIENE----------------------------- 25

gi_73669953 1 -----------------PMDLTIVGLGGCGKRLCEEICRHDWILDSYL---------------------- 31

gi_20091535 1 -----------------PTDLTILGLGGCGKKLAREICSYDWLLHEYS---------------------- 31

gi_14521848 1 -----------------TFPDVIIGVGGAGKLLIFSMLQKEWFIRELL---------------------- 31

gi_257053119 1 -----------------NLPDRIFAVGGAGKEITLRLLEANWVLEDIL---------------------- 31

gi_284164354 1 -----------------NLPERIFSVGGAGKQIALELLESEWVLREIL---------------------- 31

gi_88602022 1 -----------------IIPTLAIGVGMGGVSVVKEFISFV--EKN------------------------ 27

gi_268325165 1 -----------------TVNRVAIGIGGQGSSIVNNILRTL--KHKTG---------------------- 29

gi_269956178 1 -----------------LRPFLLVGVGGSGGKTLRIIREDL--LRRLK---------------------Q 30

gi_227497172 1 -----------------MRKVLVVGCGGSGAKTLAYMMDQL--HADLA---------------------- 29

gi_240169446 1 -----------------MRRFLIVGCGGSGGATLAYMMDQL--RSELH---------------------- 29

gi_19553917 1 -----------------MKKVLVVGCGGSGAKTLAYMMDQL--KTTLADSL---------------PERY 36

gi_182437626 1 -----------------FQPMLFVGLGGTGGLVGAELERKL--RVDLC---------------------- 29

gi_83312057 1 -----------------AATNLFLGFGGTGAHILTFLKEFT--VYKHG---------------------- 29

gi_160892492 1 -----------------NGNLLVIGLGGIGSRTVCNLKGMM--VDDITPE-------------------- 31

gi_163815161 1 -----------------NKNFLVIGLGGLGSRAVCALKGML--VNDITPE-------------------- 31

gi_189219502 1 -----------------NANHFIIGLGGTGGKVIRAFRKTI--FQEFR---------------------- 29

gi_121606151 1 ------------------MNHFIIGLGGTGGKIIRALRKSL--YQEFH---------------------- 28

gi_154496732 1 -----------------EELFIFISSGGNGHKSLCAVRKEL--EWRVA---------------------- 29

gi_238059621 1 -----------------VLPALVIGLGGTGSIAAAHLKHRL--ATEQRWQ----------------DLRR 35

gi_15805565 1 -----------------VFKTLVIGLGSTGTEILESLADRI--DWEVG---------------------- 29

gi_226358106 1 -----------------VFKTLVIGMGSTGTEILEALADRI--DWEVG---------------------- 29

gi_149916637 1 -----------------VTPTLFVGLGGSGGRAVGRIAKRL--RAQQD---------------------- 29

gi_32476397 1 -----------------TGHALVVAVGGVGAECLHELRSRV--ATLHS---------------------- 29

gi_168704658 1 -----------------LQPAIVIGLGNAGLRVLQRLRFEL--HERYG---------------------- 29

gi_168700390 1 -----------------LFPALVVALGHTGRRVVEQLKRII--TDRHG---------------------- 29

gi_87309437 1 -----------------HRPTLVIGVGGVGVRLAYAIEQKY--RDSGK---------------------- 29

gi_283778279 1 -----------------APPTIVIGIGGTAGRVLSALRKRQ--ADLPAE--------------------- 30

gi_52141958 1 -----------------QVPTILIGLGGIGSTVTHQIYERL--PEERR---------------------- 29

gi_154500451 1 -----------------DAPTLFIGIGGTGCDIVKRVAEMC--RPGET---------------------- 29

gi_153954291 1 -----------------PNPMLIIGLGGTGIDAMLRLKYQI--NKRFI---------------------L 30

gi_226315300 1 -----------------DNPMLVIGLGGTGIDALLRLKYQV--NRRFK---------------------L 30

gi_193212596 1 -----------------VVPTLFIALGGTGAQVLWRIRRRI--INNLW--------------------GS 31

gi_75908069 1 -----------------INRTICIGLGGTGRDVLMRIRRLI--VDRYG---------------------- 29

gi_37520551 1 -----------------MTPTVVVGLGGTGKEILIKIRRMI--VESYG---------------------- 29

gi_284051667 1 -----------------IVPTLIIGVGGTGLEAMTRVRRLI--VESYG---------------------- 29

gi_22298564 1 -----------------IVPTVIVGVGGTGIEVLSRVRRLV--EETYG---------------------- 29

gi_254410688 1 -----------------MVPTILIGIGGTGAEIVSRVRRLV--AETYG---------------------- 29

gi_153807774 1 -----------------IKRCLYIGLGGTGMKSLLHTKKMF--IETYG---------------------- 29

gi_219848227 1 -----------------IRPTLVIGLGGTGVDVIRQLKRRI--RQSMQ---------------------- 29

gi_17229535 1 -----------------FRPTVVIGLGGTGYEVALKLKKRF--IDVYG---------------------- 29

gi_227993086 1 -----------------LKRTVLIGLGGTGKWALLHAKKKL--LEAFG---------------------- 29

gi_90409221 1 ----------EDQTNAPFPSVCLIGLGRCGSNISLDLANLV--YNARNYYLNDFQK-----SGAKTKNQS 53

gi_34499290 1 ----------SGPTEKPFPSVCLIGLGRCGSNIALDVATLV--YNARKFYLNEFNQ----DDKSAPEQEQ 54

gi_152997985 1 ----------EGINEQVFPSVCLIGLGRCGSNIALDVASLV--YDARANYMREVES-----QEILDSESE 53

gi_87119891 1 ----------SEINDQNFPSVCLIGLGRCGSNIALDVATLV--YNARSNYMADLEL-----QDKASSEGE 53

gi_226941044 1 ----------DANAEQPFPSVCLIGLGRCGSNIALDVASLV--YNARQFYSNEFEHE--EASKSPVVETE 56

gi_192360015 1 ----------DNRKQPQFPSVCLIGLGRCGSNIALDVASLV--YNARNFYLNEFLS-----EEKAIREQD 53

gi_146308213 1 ----------ESSRIQQFPSVCLIGLGRCGSNIALDVASLV--YNARQFYLEEFHS-----EATAAIEQA 53

gi_109896728 1 ----------ESRSPQNFPSVCLIGLGRCGSNIALDVASLV--YNARNFYLNEFEN-----EERETREQE 53

gi_77457477 1 ----------EQHNDQEFPSVCLIGLGRCGSNIALDVAELV--YNARKFYLNEFNNEDRAADRRLASDKG 58

gi_70728682 1 ----------DNRRDQEFPSVCLIGLGRCGSNIALDVAELV--YNARKFYLNEFN------NEDKAYEGG 52

gi_161598655 1 ------------SVNDISIRWGVIGAGQKGNKEADLFAGYK--FSNGT---------------------- 34

gi_208703287 1 ------------EINNVAIRFGVIGAGQKGNKVADIFAGYT--FSDGT---------------------- 34

gi_169825600 1 ------------MEQKLSLNFGFLGLGMGGTSIAAACADIK--TNIKNNN-------------------- 36

gi_227811490 1 ------------SQGNISLKFGFLGLGMGGCAIAAECANKE----------------------------- 29

gi_225871538 1 ------------SQGNISLKFGFLGLGMGGCAIAAECANKE----------------------------- 29

2vam_chainA_p001 1 ------------------ASIKVIGVGGGGNNAVNRMIENE----------------------------- 23

2vap_chainA_p002 1 LELSPEDKELLEYLQQTKAKITVVGCGGAGNNTITRLKMEG----------------------------- 41

3cb2_chainA_p003 1 -----------------PREIITLQLGQCGNQIGFEFWKQL--CAEHG---------------------I 30

[Consensus_aa:](http://prodata.swmed.edu/promals3d/info/consensus.html) ....................*hhhl***G***h***G**.s**G**.p*hh*..*h*.p...............................

[Consensus_ss:](http://prodata.swmed.edu/promals3d/info/consensus_ss.html) eeeeee hhhhhhhhhhhh hh

Conservation: 5

gi_14318481 31 DFNGTYHGHDDIQKER---LNVYFNEASSGK--------WVPRSINVDLEP------------------- 70

gi_18568139 31 DPTGTYQGDSDLQLER---INVYFDEATGGR--------YVPRAVLIDLEP------------------- 70

gi_6323554 31 KPDGHLEDGLSKPKGGEEGFSTFFHETGYGK--------FVPRAIYVDLEP------------------- 73

gi_15241179 31 QPDGMMPSDTTVGVAHD-AFNTFFSETGAGK--------HVPRAVFVDLEP------------------- 72

gi_71397525 31 QPDGAMPSDKTIGVEDD-AFNTFFSETGAGK--------HVPRAVFLDLEP------------------- 72

gi_6323241 32 GTDGLSQLPDSSTERDD-DTKPFFRENSRNK--------FTPRAIMMDSEP------------------- 73

gi_71652627 32 RHDGIVEPFASAGDDR---KDVFFYQADDDH--------YIPRALLIDMEP------------------- 71

gi_15233174 32 SKDGILEDFATQGGDR---KDVFFYQADDQH--------YIPRALLIDLEP------------------- 71

gi_190016319 32 SPEAIVEEFATEGTDR---KDVFFYQADDEH--------YIPRAVLLDLEP------------------- 71

gi_161528770 25 ---------------------------------------LNSDCLLISNDS------------------- 36

gi_118194654 25 ---------------------------------------LGLDTLQISSDP------------------- 36

gi_20089831 29 --GKSCGKL------------ARFYSTQRFK--------SHVETLALNTAIN------------------ 58

gi_14521022 24 ------------------------------F--------EAIAINTSRGDL------------------- 36

gi_240102830 24 ------------------------------F--------EALAINTSRGDL------------------- 36

gi_15789554 24 -------------------------QRAEYG--------AVRGTLAVNTAK------------------- 41

gi_257052276 24 -------------------------YEMGFD--------AIRDALAVNTAE------------------- 41

gi_126178676 24 -----------------------KRMQTQSF--------RGIAVNTARTDL------------------- 43

gi_11498814 26 ----------------------KMRGSNIRM--------RWLAINSARTDL------------------- 46

gi_170290506 26 ------------------------------P--------KNVKTIAVNTDS------------------- 38

gi_182414456 26 ------------------------------L--------ERLQLGVINTDY------------------- 38

gi_41615257 26 ------------------------------I--------ENVDLIAMNTDA------------------- 38

gi_14521492 26 ------------------------------V--------QGADLIAMNTDA------------------- 38

gi_16082526 26 ------------------------------L--------VGVKLIACNTDA------------------- 38

gi_170290956 26 ------------------------------I--------DSVETVAVNTDA------------------- 38

gi_41614929 26 ------------------------------L--------QDVELIAVNADV------------------- 38

gi_11498178 26 ------------------------------V--------SSAMTIAINTDK------------------- 38

gi_15668803 26 ------------------------------I--------QGAETIAINTDK------------------- 38

gi_15789500 26 ------------------------------V--------EGADTVAINTDK------------------- 38

gi_20092672 26 ------------------------------I--------EGAETVCINTDK------------------- 38

gi_126179395 26 ------------------------------V--------SGAETIAINTDK------------------- 38

gi_15679670 26 ------------------------------V--------EGAETIAVNTDA------------------- 38

gi_11498146 26 ------------------------------I--------EGAELIALNTDV------------------- 38

gi_15789633 26 ------------------------------I--------HGADLVAANTDV------------------- 38

gi_126178359 26 ------------------------------I--------NGARLIALNTDA------------------- 38

gi_20093060 26 ------------------------------I--------QGADLVALNTDA------------------- 38

gi_14520222 26 ------------------------------V--------TGAKIIAVNTDA------------------- 38

gi_15668546 26 ------------------------------I--------EGAKTVAINTDA------------------- 38

gi_53711593 26 ------------------------------I--------HDVTFVLCNTDN------------------- 38

gi_170291053 26 ------------------------------I--------RGIKLVAINTDK------------------- 38

gi_15605992 26 ------------------------------I--------EGVELYAINTDV------------------- 38

gi_16128088 26 ------------------------------I--------EGVEFFAVNTDA------------------- 38

gi_15643599 26 ------------------------------I--------HGVEFVAVNTDL------------------- 38

gi_15639381 26 ------------------------------L--------QCVEFIAANTDV------------------- 38

gi_55981058 26 ------------------------------L--------SGVEFIAANTDA------------------- 38

gi_16330088 26 ------------------------------V--------TGIDFWAINTDS------------------- 38

gi_15609287 26 ------------------------------L--------KGVEFIAINTDA------------------- 38

gi_255767353 26 ------------------------------V--------QGVEYIAVNTDA------------------- 38

gi_73669953 32 ----------------------------VPG--------KRLRVYTMDTDA------------------- 46

gi_20091535 32 ----------------------------KGM--------NRLRIFTMDTDA------------------- 46

gi_14521848 32 --------KYTKESN------------------------ERVTFVIVDTAR------------------- 50

gi_257053119 32 ---------KPRPNP------------------------HSVTVTVIDTAE------------------- 49

gi_284164354 32 ---------QPRPNP------------------------QSVRVTILDTAE------------------- 49

gi_88602022 28 ----------------------------GII--------DNYRFVAIDSNI------------------- 42

gi_268325165 30 ----------------------------KAP--------RNEEFLIIDTDP------------------- 44

gi_269956178 31 AGWEHD----------------------DLP--------AAWQMIHIDVPN------------------- 51

gi_227497172 30 AYGIE-----------------------KIP--------GCWQFLSVDTPL------------------- 49

gi_240169446 30 AAGIES----------------------LLP--------G-WQFVVIDVPS------------------- 49

gi_19553917 37 PNPKEA----------------------KLP--------GAWQFVSVDVPT------------------- 57

gi_182437626 30 ---GPDGTALQHI--------GGLAPYQLP---------DCLQFVYADFSE------------------- 60

gi_83312057 30 ----------------------------SKP--------DGVMFLEFDTIAGWKPGQTVDIAGGGGGEEV 63

gi_160892492 32 ---------------------------------------DNIHFLMVDSDI------------------- 43

gi_163815161 32 ---------------------------------------DNINFLMIDSDI------------------- 43

gi_189219502 30 ----------KVD---------------PPD--------IRLSYLYVDSSD------------------- 47

gi_121606151 29 ----------GGP---------------PAG--------VGIGYLYVDSSS------------------- 46

gi_154496732 30 --------------------------LQELK--------DKVRFLAVDAAY------------------- 46

gi_238059621 36 DPGAPERTAGRPYD-------------------------WPVLLRALDVDR------------------- 61

gi_15805565 30 ----------GLSRA------------------------PWVEFLAVETDV------------------- 46

gi_226358106 30 ----------GLQRA------------------------PWLEFLAVETDV------------------- 46

gi_149916637 30 ---------FDLQYR------------------------SLVRFVAIDTND------------------- 47

gi_32476397 30 ----------------------------ACP--------LDLHSVLIDTDM------------------- 44

gi_168704658 30 ----------PPATT------------------------PALRTLYIDTDP------------------- 46

gi_168700390 30 ----------AAERV------------------------PHVRILHIDTDP------------------- 46

gi_87309437 30 ---------------------------EQGQ--------AWYRAIAVDTDA------------------- 45

gi_283778279 31 ---------------------------------------QQAAMILLDTDS------------------- 42

gi_52141958 30 ---------------------------------------KKVAMHVFDTDV------------------- 41

gi_154500451 30 ---------------------------------------ENINFACLDTNV------------------- 41

gi_153954291 31 EEDIISNTRKDKP--------------------------KKVEFLGFETNQ------------------- 55

gi_226315300 31 PVDQLSKKRKEKP--------------------------DNIEFIAFETNE------------------- 55

gi_193212596 32 GTGQAVRID-NLTEF------------------------PFAEFLQIDLSA------------------- 57

gi_75908069 30 ----------DLSNL------------------------PIVSFVHLDTDK------------------- 46

gi_37520551 30 ----------TLDAL------------------------PIVSFLHLDTEQ------------------- 46

gi_284051667 30 ----------SLEKL------------------------PVVGFLHIDTDE------------------- 46

gi_22298564 30 ----------SLKQF------------------------PVISFLAIDTDR------------------- 46

gi_254410688 30 ----------NLTNF------------------------PILSFLVIDTDK------------------- 46

gi_153807774 30 ----------------------------EVP--------PMIGFLGIDTDG------------------- 44

gi_219848227 30 ----------PMP--------------------------GVLEFLVVDTEM------------------- 44

gi_17229535 30 ----------------------------DVP--------DIIRFLSIDTTE------------------- 44

gi_227993086 30 ----------------------------EEP--------PLVKFLLIDTTA------------------- 44

gi_90409221 54 SLTTQWIKNTLGIKDKHNKHAAFLIEPFVILGDLDQDISGRVHYSYMDGKH------------------- 104

gi_34499290 55 RRPRRWLHRNLLAQHKA-AKPAFLIEPMVMLGDLDKDIKGRIRFSRRGEQG------------------- 104

gi_152997985 54 FRPMRWIRKHLPLEDKDGFKPVFLIEPIVLLGDLDKDIEGRIRFSNQEGRT------------------- 104

gi_87119891 54 FRPMRWIQKHLPIEIKGEPRPVFLIEPVVMLGDLDKDIEGRIRFSSQNGRS------------------- 104

gi_226941044 57 RQPKNWIRRNLARTQSRVSKPVFLIEPMVMLGDLDKDIKGRILFSRRGGDG------------------- 107

gi_192360015 54 QRPMRWIKRSLNIKSGRQLKPVFLIEPLVMLGDLDKDIDGRIRFSHKGERS------------------- 104

gi_146308213 54 SRPSRWIRSNLLRAPHKSSKPVFLIEPLVMLGDLDKDIAGRIRFSRKGEMS------------------- 104

gi_109896728 54 YRPMRWIKRGLHLSSAQDVKPVFLIEPLVMLGDLDKDIEGRVRFSNKGEKA------------------- 104

gi_77457477 59 YSPAQWIKQNLRIGPNKSTKPVFLVEPLVMLGDLDKDIAGRIRFSRKGEKS------------------- 109

gi_70728682 53 YSPGQWIRQNLRLGQNKASKPVFLVEPLVMLGDLDKDIAGRIRFSRKGEKS------------------- 103

gi_161598655 35 ---------------------------------------TCYPTLAVNFAE------------------- 46

gi_208703287 35 ---------------------------------------PCYPTLALNLSH------------------- 46

gi_169825600 37 ---------------------------------------YPYTALLINSNQ------------------- 48

gi_227811490 30 ------------------------TQIKNNK--------YPYRAILVNTNS------------------- 48

gi_225871538 30 ------------------------TQIKNNK--------YPYRAILVNTNS------------------- 48

2vam_chainA_p001 24 ------------------------------V--------QGVEYIAVNTDA------------------- 36

2vap_chainA_p002 42 ------------------------------I--------EGAKTVAINTDA------------------- 54

3cb2_chainA_p003 31 SPEAIVEEFATEGTDR---KDVFFYQADDEH--------YIPRAVLLDLEP------------------- 70

[Consensus_aa:](http://prodata.swmed.edu/promals3d/info/consensus.html) .........................................*h*p*hhhh*sss....................

[Consensus_ss:](http://prodata.swmed.edu/promals3d/info/consensus_ss.html) eeeeeee h

Conservation:

gi_14318481 71 ------------------GTIDA---------------------VRNSAIG-------NLFRPDNYIFGQ 94

gi_18568139 71 ------------------GTMDS---------------------VRAGPYG-------QIFRPDNFIFGQ 94

gi_6323554 74 ------------------NVIDE---------------------VRNGPYK-------DLFHPEQLISGK 97

gi_15241179 73 ------------------TVIDE---------------------VRTGTYR-------QLFHPEQLISGK 96

gi_71397525 73 ------------------TVVDE---------------------IRTGTYR-------QLFHPEQLISGK 96

gi_6323241 74 ------------------SVIAD---------------------VENTFRG-------FFDPRNTWVASD 97

gi_71652627 72 ------------------RVINA---------------------IQRGSMQ-------RLFNPENVYIHS 95

gi_15233174 72 ------------------RVING---------------------IQNGDYR-------NLYNHENIFVAD 95

gi_190016319 72 ------------------RVIHS---------------------ILNSPYA-------KLYNPENIYLSE 95

gi_161528770 37 ------------------KDFAG---------------------DVPSVHV------------------- 48

gi_118194654 37 ------------------DDLGP---------------------GGVL---------------------- 45

gi_20089831 59 ----------------DLKEMKF---------------------TKA---------------KDRIHIEH 76

gi_14521022 37 ------------------EYLKH---------------------IPQ---------------DKRILIGE 52

gi_240102830 37 ------------------DYLKH---------------------IPS---------------DRRILIGE 52

gi_15789554 42 ------------------TDLEG---------------------LDI----------------DTMLIGQ 56

gi_257052276 42 ------------------ADLQP---------------------LDI----------------DTMLIGQ 56

gi_126178676 44 ------------------MGLKN---------------------IEL---------------KDRLLIGQ 59

gi_11498814 47 ------------------MGLKH---------------------VPV---------------QDRILIGQ 62

gi_170290506 39 ------------------AVLKK---------------------ADA---------------DEKVLIGR 54

gi_182414456 39 ------------------QALAS---------------------SPV---------------QDKVLIGM 54

gi_41615257 39 ------------------VHLKS---------------------MKVDPER-----------VKRILLGP 58

gi_14521492 39 ------------------QHLHY---------------------VKA---------------HKKLLLGR 54

gi_16082526 39 ------------------AHLLR---------------------IRA---------------HAKILLGK 54

gi_170290956 39 ------------------QHLLI---------------------TTA---------------DRKLLIGK 54

gi_41614929 39 ------------------KDLAK---------------------IKA---------------HKKVLIGE 54

gi_11498178 39 ------------------QQLLR---------------------TKA---------------DKRVLIGR 54

gi_15668803 39 ------------------QHLEV---------------------IQA---------------DKKILIGA 54

gi_15789500 39 ------------------QHLKM---------------------IKA---------------DTKILVGK 54

gi_20092672 39 ------------------QHLDN---------------------VRA---------------DKKILVGK 54

gi_126179395 39 ------------------QHLDM---------------------IQA---------------DKRVLVGK 54

gi_15679670 39 ------------------QDLFY---------------------SVA---------------NRKLLIGK 54

gi_11498146 39 ------------------QHLYY---------------------TKA---------------NRRILIGK 54

gi_15789633 39 ------------------QHLVD---------------------IEA---------------DTKILMGQ 54

gi_126178359 39 ------------------QHLVR---------------------TRS---------------DTRILIGR 54

gi_20093060 39 ------------------QHLLH---------------------IRS---------------GKKILIGK 54

gi_14520222 39 ------------------QDLLK---------------------IKA---------------HQKILIGK 54

gi_15668546 39 ------------------QQLIR---------------------TKA---------------DKKILIGK 54

gi_53711593 39 ------------------QALAE---------------------SPV---------------PVKLQLGR 54

gi_170291053 39 ------------------VHLDG---------------------INA---------------PYKVLIGD 54

gi_15605992 39 ------------------QHLST---------------------LKV---------------PNKIQIGE 54

gi_16128088 39 ------------------QALRK---------------------TAV---------------GQTIQIGS 54

gi_15643599 39 ------------------QVLEA---------------------SNA---------------DVKIQIGE 54

gi_15639381 39 ------------------QALSY---------------------STA---------------PKKLAIGT 54

gi_55981058 39 ------------------QVLAK---------------------SLA---------------DHRIQLGE 54

gi_16330088 39 ------------------QALTN---------------------TNA---------------PDCIQIGQ 54

gi_15609287 39 ------------------QALLM---------------------SDA---------------DVKLDVGR 54

gi_255767353 39 ------------------QALNL---------------------SKA---------------EVKMQIGA 54

gi_73669953 47 ------------------NERADDEWYRRQ-------VKSRIQEMGAGG-------NIEYK-YYYLPSLA 83

gi_20091535 47 ------------------SERFQDKKLETD-------IMEKVNKLNGTG-------NILFQ-SYFLPYLA 83

gi_14521848 51 ------------------AELSKDREALKEIE---KKIDKIAKEMKVPK-------NVHIV-LKCLIEDL 91

gi_257053119 50 ------------------GEENTDKQRLQSIRNRIESIESDLRDSDQGRTG-----TIDIQ-YKLITEDI 95

gi_284164354 50 ------------------EEENSDRERIAEIRERVATLKSELRETGTGRPG-----DVSIE-YKMITRNI 95

gi_88602022 43 ------------------DDLNR---------------------IIEFAPN-----------TSKIAITD 62

gi_268325165 45 ------------------ASANA---------------------CSEI------------EERKKIILNR 63

gi_269956178 52 ------------------RADGD---------------------DVDLPAQ------LPDRQYKGLVATG 76

gi_227497172 50 ------------------QEEKP----------------------GGLGS-------VTQQGGAYVACGV 72

gi_240169446 50 ------------------GAEDG---------------------PEGLSN-------VPAQGGTYIGCGP 73

gi_19553917 58 ------------------SPESP---------------------GPNLPN-------VPEAGGRYISCGS 81

gi_182437626 61 ------------------SDLQR---------------------LPQFN----------VDSSLRAAYAR 81

gi_83312057 64 VAKGYEEANSLQPQAEYFQLMDR---------------------HPS---------------LRDLVTHH 97

gi_160892492 44 ------------------PEMEQ---------------------TIEDSKE-----HIGFNALEVLSIYR 69

gi_163815161 44 ------------------SEMER---------------------TIEDSKE-----GVGFNALEVISIYR 69

gi_189219502 48 ------------------ELMRL---------------------DDPSWKVL--GQSVQIPPTSQLLIKS 76

gi_121606151 47 ------------------EMMAM---------------------DDPTWKTL--GTSVQLSKASQLLITD 75

gi_154496732 47 ------------------KELDE---------------------LLEKY---------GFDSTEVLKLPF 68

gi_238059621 62 ------------------RTRPR---------------------VDGV--------SLDV-DTEDLYLDG 83

gi_15805565 47 ------------------AKPNR---------------------FNGTDDF-----KTLGVPATAWRDML 72

gi_226358106 47 ------------------AKPNR---------------------FNGTDDF-----KTLGIPATAWRDIL 72

gi_149916637 48 ------------------ADLAR---------------------LRKGTQE-------FGKVDETVLISD 71

gi_32476397 45 ------------------HTIHA---------------------ARLAEA-------SDRIPPATILHTP 68

gi_168704658 47 ------------------DGLEE---------------------AGRERFG----DRLAALGSDEVFPAR 73

gi_168700390 47 ------------------GAGGA---------------------DGSDPAG--------LTVREVVPARL 69

gi_87309437 46 ------------------RTMNS---------------------MEIPPP-------LPEATDITRLLIP 69

gi_283778279 43 ------------------RDLSR---------------------AVTQTG-------SAALDPEDTVHLP 66

gi_52141958 42 ------------------NTLSK---------------------FDH---------------IRKFKTQT 57

gi_154500451 42 ------------------NDLSA---------------------VAKSR------------AHIYYVQTS 60

gi_153954291 56 ------------------GEKNK---------------------RYPGNGG-----VGLDPQSELVMLSN 81

gi_226315300 56 ------------------HDRNK------------------------KYKG-----IGLDPVTEFVLLSN 78

gi_193212596 58 ------------------FETEQ---------------------GKAEKNDIL-SNKIKFKESERLVKKL 87

gi_75908069 47 ------------------AATQV---------------------TGIRTGSTYHGVDLSFREAEKVSATM 77

gi_37520551 47 ------------------NAKVS---------------------EPQTVLK----QDISLRPVEQVWTKV 73

gi_284051667 47 ------------------KPQVK---------------------KPEMA-------GPPLEDYEKFWASV 70

gi_22298564 47 ------------------DYKVS---------------------NPLAA-------GSPLKDNEKHWASV 70

gi_254410688 47 ------------------DYKIN---------------------NPDAA-------GSPLKDNEKHWASV 70

gi_153807774 45 ------------------GEYKK---------------------ELDSKYG-----NVVLTPNEQLPIRV 70

gi_219848227 45 ------------------PQNMP---------------------GEER-------------ILDREIAYI 62

gi_17229535 45 ------------------NIQSR---------------------EKSPDGT-----KVVLEPNELYAISV 70

gi_227993086 45 ------------------ANNDH---------------------LLTADGK-----KARLQASEILHIEA 70

gi_90409221 105 ------------------SLLDG---------------------YPK---------------MKIMDLSE 120

gi_34499290 105 ------------------DFINN---------------------YNK---------------LKIMDLAE 120

gi_152997985 105 ------------------KFLEE---------------------YTK---------------LQIMDLSE 120

gi_87119891 105 ------------------NFLDE---------------------YKK---------------LQIMDLSE 120

gi_226941044 108 ------------------SFLSD---------------------YNK---------------MKIMDLSE 123

gi_192360015 105 ------------------NFLKD---------------------YTK---------------MKIMDLSE 120

gi_146308213 105 ------------------GFLDD---------------------YSK---------------MKIMDLSE 120

gi_109896728 105 ------------------SFLQE---------------------YTK---------------MKIMDLSE 120

gi_77457477 110 ------------------GFLRD---------------------YSK---------------MKIMDLSE 125

gi_70728682 104 ------------------GFIRD---------------------YSK---------------MKIMDLSE 119

gi_161598655 47 ------------------SDMMH---------------------LQNIIK------------EDRIHFDG 65

gi_208703287 47 ------------------TDMIH---------------------LKNIDK------------QDRIHFDG 65

gi_169825600 49 ------------------IDLNK---------------------IEPAN----------TTSEKMLIGDG 69

gi_227811490 49 ------------------QDFNK---------------------IEIKNA----------GNVRKIQLEG 69

gi_225871538 49 ------------------QDFNK---------------------IEIKNA----------GNVRKIQLEG 69

2vam_chainA_p001 37 ------------------QALNL---------------------SKA---------------EVKMQIGA 52

2vap_chainA_p002 55 ------------------QQLIR---------------------TKA---------------DKKILIGK 70

3cb2_chainA_p003 71 ------------------RVIHS---------------------ILNSPYA-------KLYNPENIYLSE 94

[Consensus_aa:](http://prodata.swmed.edu/promals3d/info/consensus.html) ....................b..........................................p.*h*.*h*..

[Consensus_ss:](http://prodata.swmed.edu/promals3d/info/consensus_ss.html) hhhh eeee

Conservation:

gi_14318481 95 SS-------------------------------------------------------------------- 96

gi_18568139 95 S--------------------------------------------------------------------- 95

gi_6323554 98 ED-------------------------------------------------------------------- 99

gi_15241179 97 ED-------------------------------------------------------------------- 98

gi_71397525 97 ED-------------------------------------------------------------------- 98

gi_6323241 98 GA-------------------------------------------------------------------- 99

gi_71652627 96 EG-----------------------------------------------------G-------------- 98

gi_15233174 96 HG-----------------------------------------------------G-------------- 98

gi_190016319 96 HG-----------------------------------------------------G-------------- 98

gi_161528770 49 -----------------------------------------------------STDS------------- 52

gi_118194654 46 -----------------------------------------------------VPTR------------- 49

gi_20089831 77 LH-------------------------------------------------------------------- 78

gi_14521022 53 SI---------------------------------------------------VGGK------------- 58

gi_240102830 53 SL---------------------------------------------------TGGK------------- 58

gi_15789554 57 ER---------------------------------------------------VKGH------------- 62

gi_257052276 57 DR---------------------------------------------------VKGH------------- 62

gi_126178676 60 TV---------------------------------------------------VKGH------------- 65

gi_11498814 63 TI---------------------------------------------------VKGH------------- 68

gi_170290506 55 YT---------------------------------------------------HKGR------------- 60

gi_182414456 55 SI---------------------------------------------------TRGL------------- 60

gi_41615257 59 DI---------------------------------------------------TKGH------------- 64

gi_14521492 55 SI---------------------------------------------------THGK------------- 60

gi_16082526 55 NL---------------------------------------------------TRGL------------- 60

gi_170290956 55 EL---------------------------------------------------CGGN------------- 60

gi_41614929 55 EV---------------------------------------------------TRGL------------- 60

gi_11498178 55 SI---------------------------------------------------TRGL------------- 60

gi_15668803 55 TL---------------------------------------------------TRGL------------- 60

gi_15789500 55 SL---------------------------------------------------TNGL------------- 60

gi_20092672 55 TL---------------------------------------------------TRGL------------- 60

gi_126179395 55 SL---------------------------------------------------TKGL------------- 60

gi_15679670 55 NV---------------------------------------------------CGGL------------- 60

gi_11498146 55 RR---------------------------------------------------TRGL------------- 60

gi_15789633 55 QK---------------------------------------------------TKGR------------- 60

gi_126178359 55 QR---------------------------------------------------TRGL------------- 60

gi_20093060 55 KK---------------------------------------------------TRGL------------- 60

gi_14520222 55 EL---------------------------------------------------TRGL------------- 60

gi_15668546 55 KL---------------------------------------------------TRGL------------- 60

gi_53711593 55 SI---------------------------------------------------TQGL------------- 60

gi_170291053 55 SI---------------------------------------------------THGL------------- 60

gi_15605992 55 KV---------------------------------------------------TRGL------------- 60

gi_16128088 55 GI---------------------------------------------------TKGL------------- 60

gi_15643599 55 NI---------------------------------------------------TRGL------------- 60

gi_15639381 55 KV---------------------------------------------------TRGL------------- 60

gi_55981058 55 KL---------------------------------------------------TRGL------------- 60

gi_16330088 55 KL---------------------------------------------------TRGL------------- 60

gi_15609287 55 DS---------------------------------------------------TRGL------------- 60

gi_255767353 55 KL---------------------------------------------------TRGL------------- 60

gi_73669953 84 NITQTSDLASQEVA-EKIKDRKSEPL------------VKTWWMNDSGDFGLSFEELRAIDP-------- 132

gi_20091535 84 SVEHVADLTSSKVL-ASIKDSQSVT-------------ANTWWLHDPENG-ITFKDLKNIDP-------- 130

gi_14521848 92 HITNPYNLYD---------LDLLRKVKE--------GVARVWWLYDNEFG-IDYTEGLKIY--------- 134

gi_257053119 96 QLSGSIDLLGDDA--VPRITSGAGME------------QENWWIKENHIN--ENLDF------------- 136

gi_284164354 96 QLNDQNDLIGETA--VPRIAAGNGMD------------EDDWWVEEQHIN--ENLDF------------- 136

gi_88602022 63 --------------------------------------------HQYDVMNLKKNCPYLHKW-------- 80

gi_268325165 64 PD----KILMKN--------------------------TNRWLPDPY----LTAAGA------------- 86

gi_269956178 77 V-----DYTTIDTA-MKAKAGPSFVD-----------AAATWRPDPNSVN--VSPSK------------- 114

gi_227497172 73 SAGSYSVVDDSLTHQVQSKGPAGLRQ------------LATWMPGTVQDV-PFPVTV------------- 116

gi_240169446 74 QGSSYAILDAA------LSQRLAANAAL--------DTIATWAPRHPEEV-TNPIST------------- 115

gi_19553917 82 SD-RYATVDTA------VSNQLSSRGAL--------GGVSSWALRNPDSE-TTPISK------------- 122

gi_182437626 82 TSRATHNLLP---------NFDSSPEVTQMLRASLREEVAGWLPSRDGEP-RVTPLH------------- 128

gi_83312057 98 L--------------------------------------------------SPAGNPGDYPQYRDWLHSQ 117

gi_160892492 70 P--NIENILA---------DGIKKNPVH--------PNLANWMDADFPDV--TVTKD------------- 105

gi_163815161 70 P--DIENVLE---------NGIQNNKIH--------KNLANWMSPEFPEI--IIGRD------------- 105

gi_189219502 77 SN--LAAILD---------DLPSYPG------------IQPWIGDKNVWK-DILNSIVG----------- 111

gi_121606151 76 AN--LTSRLD---------NLDSYPG------------LKHWLGSPQEWR-DILNSIVG----------- 110

gi_154496732 69 E--GAHESIN---------PDTISPQ------------MKEWVDPELYEV-TGGKAITMSSQ-------- 106

gi_238059621 84 SVRSMIDKVRQG----RDVAQEFYPT------------VVPWFGPEDARE-IRDDEALSF---------- 126

gi_15805565 73 N--RPEL-------------YDASIA------------LNTWADLETLGQ-LPAQSID------------ 102

gi_226358106 73 H--RPEI-------------HEASIA------------LNTWADAETLAQ-LPAQSID------------ 102

gi_149916637 72 FDKVAYSKLRRG-----ESFADADDY------------FTQWVHDWYNF--REESGA------------- 109

gi_32476397 69 LK---SAQQY---------REGRTDH------------FRSLSRR-WIYN--VPRSG------------- 98

gi_168704658 74 LNRAGHYLKP---------RFNGRSL------------TEGWFDPQLLYK--LPRNP------------- 107

gi_168700390 70 N---RSTHYM---------QRDGLPP------------VEQWMPTGSLYQ-LPRNPG------------- 101

gi_87309437 70 LR---RPQSY---------RSESARI------------TQSVSRR-WLYN--IPNSL------------- 99

gi_283778279 67 LR----KTQD---------YRTDSRK------------ILEWLSRRWLYN--IPRSL------------- 96

gi_52141958 58 S--SSKTPRE---------YIAGDPT------------IPEWFPMDPTIL-DKPLTE------------- 90

gi_154500451 61 STQTVGDY-----------LDYDRDA------------LEHWFPKNAVMY-DKTVSE------------- 93

gi_153954291 82 A--EIRSILK---------DRKILDD-----------CIKEWLSPELSS---ESGTD------------- 113

gi_226315300 79 P--EIGGVLQ---------NRSILEP-----------YITDWLSPELTI---TDGIS------------- 110

gi_193212596 88 D---LNQYIKS------EEALDCYPL------------IREWFPLSRKTI-NELNIDP------------ 123

gi_75908069 78 SAKEVTMFVEGLERRSEYTRYGPYDH------------IARWFPPQLLRN-IKAVEE------------- 121

gi_37520551 74 E--DAKAILS---------RIGSYPY------------LAEWFPSQLKG--TDSILA------------- 105

gi_284051667 71 TFDEAKKVKD---------NPTTYSW------------YYDWLPPELTPQ-NLVSEQ------------- 105

gi_22298564 71 SGKNVQQIIQ---------NLDNYPW------------IASWFPRELERN-MTSLEA------------- 105

gi_254410688 71 SGKQVRDMVS---------NMENYPW------------INRWFPRELERN-ITSLEA------------- 105

gi_153807774 71 D--EARPI------------YEVNKE------------HFAWLPEENIYA-LTSMTL------------- 100

gi_219848227 63 GDFNAGKVLD---------HLDQHPH------------IKDWWPNGQVV--TGSIFR------------- 96

gi_17229535 71 A------NPL---------PLTRNDH------------IDEWWPRNIPT---SSLIS------------- 97

gi_227993086 71 R--GASL------------LPKVHDE------------IREWFPPKADL--KANILA------------- 99

gi_90409221 121 -----------------------------------------------------VHAG------------- 124

gi_34499290 121 -----------------------------------------------------VHAG------------- 124

gi_152997985 121 -----------------------------------------------------VHAG------------- 124

gi_87119891 121 -----------------------------------------------------VHAG------------- 124

gi_226941044 124 -----------------------------------------------------VHAG------------- 127

gi_192360015 121 -----------------------------------------------------VHAG------------- 124

gi_146308213 121 -----------------------------------------------------VHAG------------- 124

gi_109896728 121 -----------------------------------------------------VHAG------------- 124

gi_77457477 126 -----------------------------------------------------VHAG------------- 129

gi_70728682 120 -----------------------------------------------------VHAG------------- 123

gi_161598655 66 L-----------------------------------------KGAARTPS-VVTDLF------------- 80

gi_208703287 66 L-----------------------------------------QGAARTPS-VVIDTF------------- 80

gi_169825600 70 K--------------------------------------------------------------------- 70

gi_227811490 70 YE-----------------------------------------------------Q-------------- 72

gi_225871538 70 YE-----------------------------------------------------Q-------------- 72

2vam_chainA_p001 53 KL---------------------------------------------------TRGL------------- 58

2vap_chainA_p002 71 KL---------------------------------------------------TRGL------------- 76

3cb2_chainA_p003 95 HG-----------------------------------------------------G-------------- 97

[Consensus_aa:](http://prodata.swmed.edu/promals3d/info/consensus.html) .....................................................s................

[Consensus_ss:](http://prodata.swmed.edu/promals3d/info/consensus_ss.html)

Conservation: 5

gi_14318481 97 ----------------AGNVWAKG-----HYTEGAELVDSVMD------------VIRREAEGC------ 127

gi_18568139 96 ---------------GAGNNWAKG-----HYTEGAELIDSVLD------------VCRKEAESC------ 127

gi_6323554 100 ----------------AANNYARG-----HYTVGREILGDVLD------------RIRKLADQC------ 130

gi_15241179 99 ----------------AANNFARG-----HYTVGKEIVDLCLD------------RVRKLADNC------ 129

gi_71397525 99 ----------------AANNYARG-----HYTIGKEIVDLCLD------------RIRKLADNC------ 129

gi_6323241 100 ---------------SAGNSWANG------YDIGTRNQDDILN------------KIDKEIDST------ 130

gi_71652627 99 ---------------GAGNNWAHG------YEMGDSVQETLFD------------MIEREAENS------ 129

gi_15233174 99 ---------------GAGNNWASG------YHQGKGVEEEIMD------------MIDREADGS------ 129

gi_190016319 99 ---------------GAGNNWASG------FSQGEKIHEDIFD------------IIDREADGS------ 129

gi_161528770 53 ---------------VVNPSMQ----------LIRGSTYNASE------------EIKSKIS-------- 77

gi_118194654 50 --------------GVINPSSR----------YIRGCTDSVSG------------GITERIS-------- 75

gi_20089831 79 ---------------GVGANRN----------VGKQVFEEKKE------------IIMRQIEDR------ 105

gi_14521022 59 ---------------GVNANPV----------LGREAMKRDLP------------MVMKKINSMVGY--- 88

gi_240102830 59 ---------------GVNANPV----------LGREAMKRDLP------------MVMRKISSMVGY--- 88

gi_15789554 63 ---------------GVGADNE----------LGAEVMTNDVR------------EVMGGLDGVV----- 90

gi_257052276 63 ---------------GVGGDNE----------LGAEIMQSAAT------------EVLDGLDGRI----- 90

gi_126178676 66 ---------------GVGTDNV----------TGARVTADEID------------AIINAVDSRG----- 93

gi_11498814 69 ---------------GVGTDNK----------LGAKVAQEDIE------------TILNAIDERG----- 96

gi_170290506 61 ---------------GAQGVPD----------LGREAMEEDIE------------SVLRALDE------- 86

gi_182414456 61 ---------------GAGGDPE----------LGREAAEADRE------------KITNVVK-------- 85

gi_41615257 65 ---------------GAGGKPE----------VAEQAARESAK------------EIKQLLE-------- 89

gi_14521492 61 ---------------GSGGDPR----------VGYRAAEASAS------------EIAEVVK-------- 85

gi_16082526 61 ---------------GAGADPT----------VGEMAAKESES------------EILRHID-------- 85

gi_170290956 61 ---------------GSGGDPH----------IGEEAARESAD------------EIEEFLS-------- 85

gi_41614929 61 ---------------GTGRDPE----------LGEQAARESEK------------VIKELLQ-------- 85

gi_11498178 61 ---------------GAGGYPE----------IGRKAAELARN------------VLEDLLC-------- 85

gi_15668803 61 ---------------GAGGYPE----------IGRKAAEMAKN------------ILEEQLK-------- 85

gi_15789500 61 ---------------GAGGDPS----------MGERATEMAQG------------TIKEVLG-------- 85

gi_20092672 61 ---------------GAGGYPE----------TGKKAAELARG------------TLEEVLK-------- 85

gi_126179395 61 ---------------GAGGFPD----------VGRRAAEMARP------------TLESLLC-------- 85

gi_15679670 61 ---------------GAGGVPE----------VGEECAEESED------------DIRRELE-------- 85

gi_11498146 61 ---------------GAGSLPQ----------VGEEAARESED------------EIKKLVE-------- 85

gi_15789633 61 ---------------GAGSLPQ----------VGEEAAIESQG------------EIRDSIA-------- 85

gi_126178359 61 ---------------GAGSIPQ----------VGEEAALENED------------DIKLAVQ-------- 85

gi_20093060 61 ---------------GAGSLPQ----------IGEDAAIESID------------EINKIVQ-------- 85

gi_14520222 61 ---------------GAGNDPK----------IGEEAAKESER------------ELREALE-------- 85

gi_15668546 61 ---------------GAGGNPK----------IGEEAAKESAE------------EIKAAIQ-------- 85

gi_53711593 61 ---------------GAGNRPE----------RARDAAEESIE------------DIKTLLND------- 86

gi_170291053 61 ---------------GAGGRPE----------VARACAEQDAH------------KISDALGN------- 86

gi_15605992 61 ---------------GAGAKPE----------VGEEAALEDID------------KIKEILR-------- 85

gi_16128088 61 ---------------GAGANPE----------VGRNAADEDRD------------ALRAALE-------- 85

gi_15643599 61 ---------------GAGGRPE----------IGEQAALESEE------------KIREVLQ-------- 85

gi_15639381 61 ---------------GAGGDPE----------IGEKAAMEDAE------------AIASALQ-------- 85

gi_55981058 61 ---------------GAGANPE----------IGEKAALEAED------------LIAEALE-------- 85

gi_16330088 61 ---------------GAGGNPA----------IGQKAAEESRD------------EIARSLE-------- 85

gi_15609287 61 ---------------GAGADPE----------VGRKAAEDAKD------------EIEELLR-------- 85

gi_255767353 61 ---------------GAGANPE----------VGKKAAEESKE------------QIEEALK-------- 85

gi_73669953 133 ---------FLIDDFGGGVHRR----------RAISKAIFYKV------------LSQGQASGFPT---- 167

gi_20091535 131 ---------YLKDDFGGGVHRR----------RAISKAILYKV------------LNEGQSNGFPS---- 165

gi_14521848 135 -----------TSGFDFGTLRR----------RAVTKAMLYKA------------IAEGIVADIF----- 166

gi_257053119 137 ---------------AKGVVRK----------RGLGKAIYYKS------------YAEDNSISSY----- 164

gi_284164354 137 ---------------ATGVVRK----------RGLGKAMYYKA------------YAEDDELSTY----- 164

gi_88602022 81 ---------VVMQKGGALQERV----------YGRFLLDLHKE------------EITRTITAHIHDLSN 119

gi_268325165 87 ---------------GCGQHRI----------YGRAMYNVHRE------------RIFSAIGAAAAELRN 119

gi_269956178 115 ---------------GAGQYRT----------LGRIITIAGLK------------RIDDAVQRARAALTG 147

gi_227497172 117 ---------------GAGQCRG----------IGRLLILDRLS------------AVSQAVQDALARMAS 149

gi_240169446 116 ---------------GAGQYRA----------IGRMIVLSKAG------------EIRSRLQAAWDQLFR 148

gi_19553917 123 ---------------GAGQYRS----------IGRMLILSRLQ------------EIQAELRKSWDVLFS 155

gi_182437626 129 --------------NGAGQLPT----------VGRAALFATLRHS--------LQPVLEPLLQAIDAIAK 166

gi_83312057 118 WLSTVMPPSVLNITAGSAQQRQ----------IGRFSMFTNAE------------KIIAQLSKSLRELSR 165

gi_160892492 106 ---------------GAHGNRQ----------IGRLMFSNAYE------------DIRMLLFDRLEEIHD 138

gi_163815161 106 ---------------GAKGNRQ----------IGRLMFSNAYT------------DIRMLLFDKLQAVYD 138

gi_189219502 112 -------------DAKGGQKRR----------LGRFLFASHAD------------EFCSKLTHLVNEMQK 146

gi_121606151 111 -------------ATLGGQKRR----------LGRFLFACKAD------------KYREQVQTQVKLLQQ 145

gi_154496732 107 ---------SGFDSSGTAAWRQPGRVRLSQPNTIAVLTTALTN------------AINSLLKG------- 148

gi_238059621 127 ------------LVEGAGQIRS----------FGRLAFFSDVLG---------PTPVTKRLEDALDQLTL 165

gi_15805565 103 --------------SGAGHIRM----------VGRLALLYPPNY----------NEIKNAISQRVARLRN 138

gi_226358106 103 --------------SGAGHIRM----------VGRLALLYPPNY----------SEIKNAITQRVARLRN 138

gi_149916637 110 ---------------GAGQIRI----------ESRLSLNRSIE------------TGDMVAK---LQALA 139

gi_32476397 99 ---------------TTEGMRP----------LGRLAMIDHAK------------TIDSGLRESIDHLAA 131

gi_168704658 108 ---------------LTLGVRL----------FGRLAFLDHYR------------FIMAKVQAEIDAAVA 140

gi_168700390 102 ---------------PAAGVRA----------FGRLALFDHYR------------AVAQRVRQEIETFLT 134

gi_87309437 100 ---------------ATEGLRP----------LGRIAYLDHID------------TILQSIGDALDNVAE 132

gi_283778279 97 ---------------QTEGLRP----------LGRLAMVDSSD------------LIWAKLRQVMKLAIS 129

gi_52141958 91 ---------------GAGQLRV----------ISRLALRAAMK------------EDKLTSFWQEIEKIF 123

gi_154500451 94 ---------------GAGQVRA----------ISRLALNATIK-----------TGRIRPLYDAIDDLF- 126

gi_153954291 114 ---------------GAGGVRQ----------VGRLLLFTKIN------------EIVDCIEKKIRLLQE 146

gi_226315300 111 ---------------GASGVRQ----------AGRLLLFTKIT------------QVVQTIEKKVKMLCE 143

gi_193212596 124 -------------EKGAGQIRA----------LSRLFFFDKYQ------------EIKGAIRTKCDSLLD 158

gi_75908069 122 ---------------GAKGIRP----------VGRLAFFHNYQ------------KIKIAIETAERLSRG 154

gi_37520551 106 ---------------GAGQIRA----------LGRFAFAVNYQ------------QVKGAFAAARGRLRG 138

gi_284051667 106 ---------------GAGQIRA----------CGRFAFFYNHE------------KIRNKCQQAITRITV 138

gi_22298564 106 ---------------GAGQIRA----------CGRFAFFCNYH------------GIQQKFQAASDRVKG 138

gi_254410688 106 ---------------GAGQIRA----------CGRFALWCNYH------------EIRDKFLAACQRVKG 138

gi_153807774 101 ---------------GAGQIRS----------NGRFAITVNHT------------EVENKIVSVLTDITR 133

gi_219848227 97 ---------------GARQRRL----------VGRLSLYARWG------------QFAHRLDTKLDKIRQ 129

gi_17229535 98 ---------------GAGQIRA----------RGRLAFFAKVG------------DINGLISQAINTVRE 130

gi_227993086 100 ---------------GAGQIRA----------LGRLALFANAS------------LVYENLRDLLALARD 132

gi_90409221 125 ---------------GSGNAPV----------LGQYLAKIILNKGAEKFLNKDWKSIHSYLIDSCG---- 165

gi_34499290 125 ---------------GAGNAPI----------LGQYLAKIILNKDTLSFSDPDWTLIHSYLVDSCG---- 165

gi_152997985 125 ---------------GAGNAPI----------LGQYLAKIILNKDATTFRNENWKQMHSYLVDSCG---- 165

gi_87119891 125 ---------------GAGNAPI----------LGQYLAKIILNKDTNSFNNSAWRHMHSYLVDSCG---- 165

gi_226941044 128 ---------------GAGNAPI----------LGQYLAKIILNKDTQRFSNADWKFIHSYLIDSCG---- 168

gi_192360015 125 ---------------GSGNAPI----------LGQYLAKIILNKDTQRFSNPDWKFVHSYLIDSCG---- 165

gi_146308213 125 ---------------GAGNAPI----------LGQYLAKIILNKDTQRFSNEDWKFIHSYLIDSCG---- 165

gi_109896728 125 ---------------GAGNAPI----------LGQYLAKIILNKDTQRFANKDWKYIHSYLIDSCG---- 165

gi_77457477 130 ---------------GAGNAPI----------LGQYLAKIILNKDTQRFSSPDWKMIHSYLIDSCG---- 170

gi_70728682 124 ---------------GAGNAPI----------LGQYLAKIILNKDTQRFSSPDWKMIHSYLIDSCG---- 164

gi_161598655 81 ---------------DPETNPN----------ANGYLDKLAQE------------LGRKFTNEEG----- 108

gi_208703287 81 ---------------DPERNPN----------AHDQLAKLYGA------------IERKFTDSEG----- 108

gi_169825600 71 ---------------GAGRDIL----------IGEQLYENDQE------------DIKQRVETRFK---- 99

gi_227811490 73 ---------------GAARNPQ----------VGEEAFVKHET------------KIFETVKQEF----- 100

gi_225871538 73 ---------------GAARNPQ----------VGEEAFVKHET------------KIFETVKQEF----- 100

2vam_chainA_p001 59 ---------------GAGANPE----------VGKKAAEESKE------------QIEEALK-------- 83

2vap_chainA_p002 77 ---------------GAGGNPK----------IGEEAAKESAE------------EIKAAIQ-------- 101

3cb2_chainA_p003 98 ---------------GAGNNWASG------FSQGEKIHEDIFD------------IIDREADGS------ 128

[Consensus_aa:](http://prodata.swmed.edu/promals3d/info/consensus.html) ...............t*h*s..............*h***G**p.*hh*....p.............*l*...*h*.........

[Consensus_ss:](http://prodata.swmed.edu/promals3d/info/consensus_ss.html) hh hhhhhhhhhhh hhhhhhhhh

Conservation: 5 5986757

gi_14318481 128 ------------------------------DSLQGFQITHSLGGGTGSGMGTLLISKIREEFP------- 160

gi_18568139 128 ------------------------------DCLQGFQICHSLGGGTGSGMGTLLISKLREEYP------- 160

gi_6323554 131 ------------------------------DGLQGFLFTHSLGGGTGSGLGSLLLEELSAEYG------- 163

gi_15241179 130 ------------------------------TGLQGFLVFNAVGGGTGSGLGSLLLERLSVDYG------- 162

gi_71397525 130 ------------------------------TGLQGFLVYHAVGGGTGSGLGALLLERLSVDYG------- 162

gi_6323241 131 ------------------------------DNFEGFQLLHSVAGGTGSGLGSNLLEALCDRYP------- 163

gi_71652627 130 ------------------------------DSLEGFVLTHSIAGGTGSGMGSYLLEHLNDRFP------- 162

gi_15233174 130 ------------------------------DSLEGFVLCHSIAGGTGSGMGSYLLETLNDRYS------- 162

gi_190016319 130 ------------------------------DSLEGFVLCHSIAGGTGSGLGSYLLERLNDRYP------- 162

gi_161528770 78 -------------------------------GYSTIVMMSNLAGKAGSAMAPVVSEMCKES--------- 107

gi_118194654 76 -------------------------------GCGTAVIFANLAGRSGSAIAPLVSRICRQL--------- 105

gi_20089831 106 ------------------------------GNFDMAFVITSASGGTGSSFTPLLVKEMKKRY-------- 137

gi_14521022 89 ------------------------------EDVDIFFLTFGFGGGTGAGGTPVLAEALKEEY-------- 120

gi_240102830 89 ------------------------------EDVDIFFLTFGFGGGTGAGGTPVLAEALKEEY-------- 120

gi_15789554 91 -----------------------------DPHTEAIFVIAGLGGGTGSGGAPVLVKELQRVYE------- 124

gi_257052276 91 -----------------------------TSQAEGIFVVAGLGGGTGSGGAPVLAKELKRIYD------- 124

gi_126178676 94 -----------------------------THDIDAFVIVAGLGGGTGSGGSPVLARHLKRIYR------- 127

gi_11498814 97 -----------------------------THDMDAFLIVAGLGGGTGSGGAPVLAKYLSEMYS------- 130

gi_170290506 87 -------------------------------NVGIVIGIAGMGGGTGSGGLPVLMREIGLRKR------- 118

gi_182414456 86 -------------------------------DCDLVFLIGGMGGGTGSGALPVVAEIASEQ--------- 115

gi_41615257 90 -------------------------------GADLVWVVAGMGGGTGTGAAPVVAEIAQNV--------- 119

gi_14521492 86 -------------------------------GYDLIFLTAGMGNGTGTGATPVIARIIKETARNN--GLP 122

gi_16082526 86 -------------------------------ETSIVFITAGFGGGTGTGAAPYVAKLAKDR--------- 115

gi_170290956 86 -------------------------------GSDLLFIMAGLGGGTGTGASPVIAEIGKRV--------- 115

gi_41614929 86 -------------------------------GTDMVFITFGLGGGTGTGAGPVIADIAKQM--------- 115

gi_11498178 86 -------------------------------DSDMVFVCAGMGGGTGTGSAPVVADVAKKQ--------- 115

gi_15668803 86 -------------------------------GADLVFVTAGMGGGTGTGSAPVVAEVAKEN--------- 115

gi_15789500 86 -------------------------------DADLVFVTAGMGGGTGTGAAPVVSKIAKEQ--------- 115

gi_20092672 86 -------------------------------NVDLVFITAGLGGGTGTGVAPVVAEVAKEQ--------- 115

gi_126179395 86 -------------------------------DADLVFITAGMGGGTGTGTAPVVAQIAKEQ--------- 115

gi_15679670 86 -------------------------------GADMVFVTCGLGGGTGTGSAPVISKLAKKA--------- 115

gi_11498146 86 -------------------------------GSDMVFVTCGLGGGTGTGAAPVVAEAAQEA--------- 115

gi_15789633 86 -------------------------------GSDMVFVTAGLGGGTGTGSAPVVAKAAREQ--------- 115

gi_126178359 86 -------------------------------GCDMVFITTGLGGGTGTGSAPVVAKAAREE--------- 115

gi_20093060 86 -------------------------------GSDMVFITAGLGGGTGTGSAPIVAEAARDA--------- 115

gi_14520222 86 -------------------------------GADMVFVTCGLGGGTGTGAAPVIAEMAKKM--------- 115

gi_15668546 86 -------------------------------DSDMVFITCGLGGGTGTGSAPVVAEISKKI--------- 115

gi_53711593 87 -------------------------------GTKMVFITAGMGGGTGTGAAPVIARIAKEM--------- 116

gi_170291053 87 -------------------------------RPDLVFIAAGMGGGTGTGAAPVVAKIAKDK--------- 116

gi_15605992 86 -------------------------------DTDMVFISAGLGGGTGTGAAPVIAKTAKEM--------- 115

gi_16128088 86 -------------------------------GADMVFIAAGMGGGTGTGAAPVVAEVAKDL--------- 115

gi_15643599 86 -------------------------------DTHMVFITAGFGGGTGTGASPVIAKIAKEM--------- 115

gi_15639381 86 -------------------------------GANMVFITAGMGGGTGTGAAPVIAKIAREL--------- 115

gi_55981058 86 -------------------------------GADLVFITAGMGGGTGTGSAPVVADIAKRL--------- 115

gi_16330088 86 -------------------------------GTDLVFITAGMGGGTGTGAAPIVAEVAKEM--------- 115

gi_15609287 86 -------------------------------GADMVFVTAGEGGGTGTGGAPVVASIARKL--------- 115

gi_255767353 86 -------------------------------GADMVFVTAGMGGGTGTGAAPVIAQIAKDL--------- 115

gi_73669953 168 -----------------------------FPSKGTTALIVGLGGGTGSGMFIDLARYIRALKG------- 201

gi_20091535 166 -----------------------------FSSPGDVAMIVGIGGGSGSGMFIDLARYIKKGRD------- 199

gi_14521848 167 ---------------------------QLRGKVSNVAMIVGLGGGTGSGLFIDLAKYLKDSKR------- 202

gi_257053119 165 ---------------------------IDLPSKGKVAVIAGLGGGTGSGILIDLARHLQQRQR------- 200

gi_284164354 165 ---------------------------IDLPDKGKVAVLAGLGGGTGSGIVIDLARHLQKKQR------- 200

gi_88602022 120 LWK-------------EKEGG------GEKRGHIAIWIIHSLGGGTGSGSFPALAIYLQKIVKEI--LGN 168

gi_268325165 120 RT---------------------------GGRDFLILMVCAFGGGTGSSMLLDIAIDIRDWISKQ--FGA 160

gi_269956178 148 TDVVGEMQ---EVSRLLGGRA------HASIGDPTVIVITSIAGGTGAGSAIDVCDVIRALPDKW----- 203

gi_227497172 150 TQSVSEAAE-VARRVPGVGEP------PATTAPPMVLVVSSMAGGSGASMTLDVCRVIAGTQTTP--AID 210

gi_240169446 149 VETISAMSTAQ--VPGIGQFD--------PHQPPLVLVVSSMAGGAGASMALDVCRLLTLVSGLD----- 203

gi_19553917 156 GETERELADLRSALYGTSVSS-----GETSKEQPIIFVVSSMAGGAGASMALDICRLLTGLEGNA----- 215

gi_182437626 167 SAG------------ELSELG------GGRVTGCDVFVAFSVAGGTGAGIFLDYLHLINQAFKMR--RFN 216

gi_83312057 166 ---------------------------AAAGGGINVWVIGSAAGGTGAGCMLDAGYLARLAAKQA--GNI 206

gi_160892492 139 ---------------------------KANGNWMDVIIVSSLSGGTGSGILSDLAYNIRAYGKAK--KWA 179

gi_163815161 139 ---------------------------KTEEGTVDVLIVSGVSGGTGSGILSDVAYNIRAYGKAK--KWN 179

gi_189219502 147 ----------------------------GGQQNVTFHVCCGLAGGTGSGTLIDCICQIRKNYPDP----- 183

gi_121606151 146 ----------------------------SGETDVTFHIVVGLAGGTGSGSVIDAVAQLRDLYPDS----- 182

gi_154496732 149 ---------------------------KPAGMRLNIIFLGGLAGGTSGGTMVDLPFLTRQIVRNI--SVA 189

gi_238059621 166 ---------------------------VDADSMPNIYVVSSTAGGTGAGILLDVLAYLQKLRQEH----- 203

gi_15805565 139 LTEAQAKAALNKDNAGLEMSVQFAVNQASGQTGVRVIVVGTLCGGTCSGTASDVGILLRTILSEE----- 203

gi_226358106 139 LTDAQAKAALNVNNAGLEMDVQFAVNASTGQTGVRVIVVGTLCGGTCSGTASDIGILLRTVLEDE----- 203

gi_149916637 140 NDLR-----------SHAHGM------RHQSASIQVFVYFSVAGGTGSGGFLPFAYLMRDILDDR----- 187

gi_32476397 132 ---------------------------VCGDRIPSVYIIGSLSGGTASGMVMDLAPRIRTILDEA--GLE 172

gi_168704658 141 PDALL--------LTEARTGL------KRRTNRPRVYVVAGTGGGTGGGMFLDLAYAVRSRLKRM--GYD 194

gi_168700390 135 DDPLH--------QADKATSL------GLRTNRPRAYVIAGLAGGTGSGMFLDVAYLLRHELRQV--GYL 188

gi_87309437 133 QSQ---------------LTP------NAVGGDPRIILMASINGGAGGGIVLDMAFAISHLLQQR--GQH 179

gi_283778279 130 SG-------------------------SSNQLSPRVIVVASIAGGTGSGMVIDIGYAARQVLAEL--GHA 172

gi_52141958 124 PVTS-----------------------DQTEYGVRVIIVTSLAGGTGSGMFLQIALYLREMLRKK--LQH 168

gi_154500451 127 RKDG-----------------------KALKQAMRIVIVSTASGGTGSGILLPLAMLVRNYVNTK--YTN 171

gi_153954291 147 ----------------------------DKEETLHVFILSGLSGGTGSGTFIDIAYIVRGIMNNI--YGS 186

gi_226315300 144 ----------------------------GTNKKLTVFLLSGLSGGTGSGCFLDIAYIVRGILERD--FGS 183

gi_193212596 159 NVKSG--------TAQKRLGL------NVQTGALKIVVVASTAGGTGSGSFLDLGYLSTIIGNDV--ANQ 212

gi_75908069 155 HDA------------LLLRKG------LRVEPGLNIFVIGSLCGGTGSGMFLDVAYSLRHLYGEQ----- 201

gi_37520551 139 HEKF-----------MLDTWK------VQLDQGINIFVVGSLSGGTGSGMLLDLAYNLRDWVPPS----- 186

gi_284051667 139 GRNQ----------LTIDGDV------LTVEPKLNIFVVGSISGGTGSGMLIDLGYCLRNWFQGQ----- 187

gi_22298564 139 HESF----------MQSRYGL------KVNNSSLNVFITGSLSGGTGSGMLIDLGYCVRHWLRGQ----- 187

gi_254410688 139 RENF----------MLDRYGI------KVSTNAINVFITGSLSGGTGSGMLIDMGYSIRKWLQGE----- 187

gi_153807774 134 ANI------------SNNEKY------ELLANEIEVHIVFSLCGGTGCGTFINMAYLLRKFAPN------ 179

gi_219848227 130 IAENE--------QVEKQGIR------TERTGQVRVYIVSSLCGGTGSGLLLDVAFRVRSKLGDD----- 180

gi_17229535 131 IRSSK--------QAFLDKFQ------VSNRDGVEVFIVGSLAGGTGSGTFLDVAFLARQYLNSF----- 181

gi_227993086 133 YKDERP-------SGERRYIY------EPYTPHLTVAVVGSLAGGTGSGTFLDVAFLLRQLMKDE----- 184

gi_90409221 166 ---------------------------IKANQSRLFFYIFSAGGGTGSGMASEFGLAQQYAYMSKTLHTE 208

gi_34499290 166 ---------------------------IKANQSRLYFYIFSAGGGTGSGMASEFGLAQQYAYMSKTFETR 208

gi_152997985 166 ---------------------------IKANQSRLYFYIFSAGGGTGSGMASEFGLAQQFSYLSKTFDYR 208

gi_87119891 166 ---------------------------IKANQSRLYFYIFSAGGGTGSGMASEFGLAQQYSYMSKTFDYH 208

gi_226941044 169 ---------------------------IKANQSRLYFYIFSAGGGTGSGMASEFGLAQQYSYMSKTFDTR 211

gi_192360015 166 ---------------------------IKANQSRLYFYIFSAGGGTGSGMASEFGLAQQYAYMSKTFETK 208

gi_146308213 166 ---------------------------IKANQSRLYFYIFSAGGGTGSGMASEFGLAQQFAYMSKTFDSK 208

gi_109896728 166 ---------------------------IKANQSRLYFYIFSAGGGTGSGMASEFGLAQQYAYMSKTFETR 208

gi_77457477 171 ---------------------------IKANQSRLYFSIFSAGGGTGSGMASEFGLAQQHSYMNKTFDTK 213

gi_70728682 165 ---------------------------IKANQSRLYFSIFSAGGGTGSGMASEFGLAQQYSYMNKTFDTK 207

gi_161598655 109 -----------------------------EVIVDQFLICLGAGGGVGTGWGSLVLQLIREQFF------- 142

gi_208703287 109 -----------------------------ELIIDHILLDIGAGGGVGTGFGSLFLKIISEGNF------- 142

gi_169825600 100 -------------------------------EVDYVWLVAGLGGGTGTGAVIQAIGTLLKSGFN------ 132

gi_227811490 101 ------------------------------EDRDFIWITCGLGGGTGTGALLKAIEMLYEH--------- 131

gi_225871538 101 ------------------------------EDRDFIWITCGLGGGTGTGALLKAIEMLYEH--------- 131

2vam_chainA_p001 84 -------------------------------GADMVFVTAGMGGGTGTGAAPVIAQIAKDL--------- 113

2vap_chainA_p002 102 -------------------------------DSDMVFITCGLGGGTGTGSAPVVAEISKKI--------- 131

3cb2_chainA_p003 129 ------------------------------DSLEGFVLCHSIAGGTGSGLGSYLLERLNDRYP------- 161

[Consensus_aa:](http://prodata.swmed.edu/promals3d/info/consensus.html) ...................................*hhlh*.t*h*t**GGTG**o**G***hh*..*h*...*h*p...........

[Consensus_ss:](http://prodata.swmed.edu/promals3d/info/consensus_ss.html) eeeeee hhhhhhhhhhhhh

Conservation: 7 5 5

gi_14318481 161 -----------------DRMMATFSVLPSPKT---------SDTVVEPYNATLSVHQ-LVEHSD------ 197

gi_18568139 161 -----------------DRIMMTFSIIPSPKV---------SDTVVEPYNTTLSVHQ-LVENSD------ 197

gi_6323554 164 -----------------KKSKLEFAVYPAPQV---------STSVVEPYNTVLTTHT-TLEHAD------ 200

gi_15241179 163 -----------------KKSKLGFTIYPSPQV---------STAVVEPYNSVLSTHS-LLEHTD------ 199

gi_71397525 163 -----------------KKSKLGYTVYPSPQV---------STAVVEPYNSVLSTHS-LLEHTD------ 199

gi_6323241 164 -----------------KKILTTYSVFPARSS----------EVVVQSYNTILALRR-LIEDSD------ 199

gi_71652627 163 -----------------KKLIQTYSVFPNQS---RGG---DSDVIVQPYNSLLAIKR-LTLHAD------ 202

gi_15233174 163 -----------------KKLVQTYSVFPNQMET--------SDVVVQPYNSLLTLKR-LTLNAD------ 200

gi_190016319 163 -----------------KKLVQTYSVFPNQDEM--------SDVVVQPYNSLLTLKR-LTQNAD------ 200

gi_161528770 108 -----------------DIGLVSFAIMPFKY-----------E-KDRIFNSGVSLKR-VRENSE------ 141

gi_118194654 106 -----------------GRPAVSFAMMPFGF-----------E-KDRIANSGTALKR-LREDSG------ 139

gi_20089831 138 -----------------NYPVYCLVVLPFRE-----------EGTLYLQNTAFSIQE-IRQNGA------ 172

gi_14521022 121 ----------------PDSLVVAIGALPLKE-----------EGIRPTINAAITIDK-LSKVVD------ 156

gi_240102830 121 ----------------PDSLVVAIGALPLKE-----------EGIRPTINAAITIDK-LSKVAD------ 156

gi_15789554 125 ------------------IPVYALGILPGRD-----------EGGIYQANAGRSLKT-LVREAD------ 158

gi_257052276 125 ------------------VPVYVLGILPGRS-----------EGSIYQANAGRSLKT-VAREAD------ 158

gi_126178676 128 ------------------EPVYAIGILPAPE-----------EGRLYSYNAARSLST-LVNEAD------ 161

gi_11498814 131 ------------------EPVYAVGILPAPE-----------EGKLYSLNAARSMIS-LLKYVD------ 164

gi_170290506 119 -----------------EVIKISVVTLPMRE-----------EGEERKRNAQFSLKE-TLEVSD------ 153

gi_182414456 116 -----------------GALVIAFVTMPFSF-----------EGGRRLKQAEEGLSA-LRRVCD------ 150

gi_41615257 120 -----------------GALVTSFAITPFRF-----------E-GRRLQIAWEGIRR-LTEFSN------ 153

gi_14521492 123 ----------------QEPLVISVVTFPFKM-----------EGRVRIEKAKAGIEM-LLEYSD------ 158

gi_16082526 116 -----------------GALTIAFATLPFSS-----------EGYVRMKNAAEGIRK-LVKNSD------ 150

gi_170290956 116 -----------------GAAVVSVVTLPFTA-----------EGAKKREIAMKGLAK-LASVSD------ 150

gi_41614929 116 -----------------GILTVAVVSWPFSS-----------EGNLTLRNAQWGLAR-LEETTD------ 150

gi_11498178 116 -----------------GAIVIGFAQMPFRV-----------E-RARIQKALDGLEE-MKEVCD------ 149

gi_15668803 116 -----------------GAIVVGVVTYPFKI-----------E-RARMKKADEGIAR-MSEVCD------ 149

gi_15789500 116 -----------------GAIVVGMVSTPFNV-----------E-RARTVKAEEGLEK-LREKAD------ 149

gi_20092672 116 -----------------GAIVVGMVSSPFRV-----------E-RARIFKAEEGLED-LRRAAD------ 149

gi_126179395 116 -----------------GAIVVGMVSYPFQV-----------E-KARLLRAEEGLEQ-LSASAD------ 149

gi_15679670 116 -----------------GALTIAVATMPFSA-----------EGLKRRENAERGLEK-LQSAAD------ 150

gi_11498146 116 -----------------GALTIAVVTFPFSA-----------EGAVRRANAEAGLER-LREVAD------ 150

gi_15789633 116 -----------------GALTIAIVTTPFTA-----------EGEVRRTNAEAGLER-LRDVAD------ 150

gi_126178359 116 -----------------GALTIAVVTLPFTV-----------EGAIRGQNAEAGLER-LREVAD------ 150

gi_20093060 116 -----------------GALTIAVVTLPFSV-----------EGHVRRTNAEAGLER-LRDVAD------ 150

gi_14520222 116 -----------------GALTVSVVTLPFTM-----------EGIRRAKNAEYGLKR-LAKASD------ 150

gi_15668546 116 -----------------GALTVAVVTLPFVM-----------EGKVRMKNAMEGLER-LKQHTD------ 150

gi_53711593 117 -----------------DILTVGIVTIPFIF-----------EGEKKIIQALDGVER-IAQHVD------ 151

gi_170291053 117 -----------------GAKIIAFVTLPFRT-----------EGRHKYKLAQEGIRQ-LRKWAD------ 151

gi_15605992 116 -----------------GILTVAVATLPFRF-----------EGPRKMEKALKGLEK-LKESSD------ 150

gi_16128088 116 -----------------GILTVAVVTKPFNF-----------EGKKRMAFAEQGITE-LSKHVD------ 150

gi_15643599 116 -----------------GILTVAIVTTPFYF-----------EGPERLKKAIEGLKK-LRKHVD------ 150

gi_15639381 116 -----------------GALTVAVVTKPFRF-----------EGRAKMMLAERGIEK-LRTHSD------ 150

gi_55981058 116 -----------------GALTVAVVTRPFSF-----------EGPKRMRAAEEGIKK-LKERVD------ 150

gi_16330088 116 -----------------GCLTVGIVTRPFTF-----------EGRRRAKQAEEGINA-LQSRVD------ 150

gi_15609287 116 -----------------GALTVGVVTRPFSF-----------EGKRRSNQAENGIAA-LRESCD------ 150

gi_255767353 116 -----------------GALTVGVVTRPFTF-----------EGRKRQLQAAGGISA-MKEAVD------ 150

gi_73669953 202 ----------------ESSQIWLFAVIPTTK-----------EGEKEQLNAAIALTE-LEYLNL------ 237

gi_20091535 200 ------------------NKIYLFAVLPTTK-----------EGVKEQLNAAISITE-LEYLNV------ 233

gi_14521848 203 -----------------DANITLFGILPSLK-----------EDEVAKANAFVAVSE-LEYLML------ 237

gi_257053119 201 -----------------TAEITLFGVMPNHT-----------EGIKENTNAFAALSE-LEYISL------ 235

gi_284164354 201 -----------------TAEITLFGILPNHT-----------EGIRENANAFAALSE-LEYLNL------ 235

gi_88602022 169 K-------------GIT-PHIYGVGILPSGTN-------------------------------------- 186

gi_268325165 161 -----------------DPVMFGIGILPSSK-----------ESVLPTGNALGTLKE-LHALMS------ 195

gi_269956178 204 -----------------ANDSVGFLYAPDVFDHLPD-----EARRGVRANALGTLAE-VLNGYWN----- 245

gi_227497172 211 -----------------PQLISVFLYTAEVFNEVPK-----DKKDGMPGNTLAMLGE-IIAAQS------ 251

gi_240169446 204 -----------------PRLMGLFLVTPDIFDSLPE-----SARTGVRANSLAMLGE-IVASQS------ 244

gi_19553917 216 -----------------VGLSSLFMVTPDIFSQLSP-----DQVAGTNPNALAMFAE-LAAAQM------ 256

gi_182437626 217 -----------------GVKIYPLVVMPSSFPAAT------GGGREAELNAARSLVD-LFRLVD------ 256

gi_83312057 207 -----------------PITLLTAVIFPEVYSG---------KFGISQARAYSLFRE-LDRLQE------ 243

gi_160892492 180 -----------------NLRIGGCLLMPDVIFGNKSVTQDPELMFRMMANGCAALKE-VDYYMK------ 225

gi_163815161 180 -----------------NLRIGGCLLMPDVLFGYKAVYEDTELVSRLNANGCATMKE-VDYYMK------ 225

gi_189219502 184 ----------------LGYRIILYTLLPEEHPKPN------WNTGNYHANGYAALVE-LNALSI------ 224

gi_121606151 183 ----------------KRFRILIYALLPDAYPHPN------WDTGNYHANGFAALTE-LNAMSV------ 223

gi_154496732 190 R-------------YKN-TGVSAYLMLPSACGSEPD----PVRKEKGNRNAYAALKE-IDYFMG------ 234

gi_238059621 204 --------------GGG-FTVTLFCVLSGAFRRRLE----GPQRTRSEANGYALLRE-LDRLMN------ 247

gi_15805565 204 ----------------EKTLGMFTLPHPDLGIAQK------PDAEIWKTNAYHALAE-LNQYHL------ 244

gi_226358106 204 ----------------EKTLAMFTLPHPNLSISQK------SDAEIWKTNAYHALAE-LNQYHL------ 244

gi_149916637 188 -----------------SARLVGFAILPEAFESVVG-----MNRDGVYANGYAALKE-CEHLMK------ 228

gi_32476397 173 -----------------SASVLPLLSTVSLQGNP--------HQPLTLHDSFAAISE-IGHYML------ 210

gi_168704658 195 -----------------RPDVRGLMVVPPADATL--------TSPQALGNTYAALTE-LNHYSR------ 232

gi_168700390 189 -----------------RPEVVGVFFVPRAEVTS--------PRNAALANTYAALAE-LHHFQS------ 226

gi_87309437 180 -----------------LSDLEVLLIHAGPRKAG--------EQDLARANSLACMTE-LHQIL------- 216

gi_283778279 173 -----------------HAGVSGVLLSGTNRNPQ--------SQQLAQVNSFALLEE-LAQLEH------ 210

gi_52141958 169 ----------------HNILIRGAFLMPDVLVKTRTV--SAKEFETVQANGYASLKE-LHAITL------ 213

gi_154500451 172 ----------------TSLIVRSVILLPETLDSVIDS---TAERESQRRNAYATIKE-INAFMM------ 215

gi_153954291 187 KG------------DDK-VNIMGYLFTPDVNLSRSAD--NQSAQSYIIKNGFAALKE-LDYLMG------ 234

gi_226315300 184 AG------------VDK-VNTLGYLFTPDVNLSNKSL--SSHTRDYIMKNGYAALKE-LDYWMN------ 231

gi_193212596 213 -----------------GVTTNLVLLLPSGYKGA--------GLTRTEANTYAALME-LETCMR------ 250

gi_75908069 202 ----------------GAQIVSYLVISPELYG----------NTPNMSANTYAALKE-LNYYST------ 238

gi_37520551 187 ----------------DLPQSSAYLVLPGAFSG---------LGDRVIANAYAALME-LDYYSR------ 224

gi_284051667 188 -----------------RLETTAIIPTPDAFLGIG-------GNIKTQENGYAALME-LNYFSD------ 226

gi_22298564 188 ----------------SSPLVTAIVPMPNAFAAIS-------VGDRVLANGYAALME-LSYFSD------ 227

gi_254410688 188 ----------------GSPLITAIVPMPNAFVGIS-------VGDRVLANGYAALME-LSYFSD------ 227

gi_153807774 180 ------------------CKLTGYGVLPDVFEAMSN-----AGMAKVKPNAYGAIQD-LDWLMH------ 219

gi_219848227 181 ------------------GEICGVFVMPSVFLQEIAS---YVQRLRIQGNAYAALKE-LNYYLS------ 222

gi_17229535 182 ------------------SNVTGLFVLPRVFANLP-------QTHLVKSNAYGALKE-IEHFWN------ 219

gi_227993086 185 ------------------DQLFGYFLLPDIYTNRP-------GTQNVEANAYGALKE-LDHFMN------ 222

gi_90409221 209 KKIQSERRSDAHSFVFEPIFSSGICILPNIACQSV------EISEALHINAGRLLCKYLSEEWD------ 266

gi_34499290 209 NEGKADQ-GDDYGFVFEPIFTSGICILPNISGQHA------EGSEALHINAGRLLCKYLSEEWD------ 265

gi_152997985 209 SDQSQMA-DKRHSFVFEPIFTSGICILPNISGKNV------EISEALHINAGRLLTK-YISEEW------ 264

gi_87119891 209 SAQSKVL-DKRHSFVFEPIFTSGICILPNISGDKI------EVSEALHINAGRLLCKYLSEEWD------ 265

gi_226941044 212 DESQIDD-GSGRSFVFEPIFTSGICILPNISDSTS------SGAEALHINAGRLLCKYLAEEWD------ 268

gi_192360015 209 SLNDNSA-NHGHSFVFEPIFTSGICILPNISDHGV------EMSEALHINAGRLLCKYLSEEWD------ 265

gi_146308213 209 APEDGEA-ERDRGFVFEPIFTSGICILPNISDQRS------EMSEALHINAGRLLCKYLAEEWD------ 265

gi_109896728 209 AVNESED-NRGHSFVFEPIFTSGICILPNISDHGV------EMSEALHINAGRLLCK-YMAEEW------ 264

gi_77457477 214 PMDEHDG-KSGHSFVFEPIFTSGICVLPNISDHRS------EMSEALHINAGRLLCKYLSEEWD------ 270

gi_70728682 208 PMDEHDS-KSGHSFVFEPIFTSGICVLPNISDHRS------EMSEALHINAGRLLCKYLSEEWD------ 264

gi_161598655 143 -----------------PCPVSMLISLPSGDP----------DEINNALVLLSEIDE-FMREQDRLFGNS 184

gi_208703287 143 -----------------PVPVTMMISLPHDNP----------EELENALVLINEINE-FFKQQNAYNMF- 183

gi_169825600 133 ------------------GRFGMILTLPRLK-----------EGRTVIENALQRLQK-ISQAMGK----- 167

gi_227811490 132 -----------------DYNFGLLLTLPRDA-----------EALKVLENATSRIRS-IAMNQEA----- 167

gi_225871538 132 -----------------DYNFGLLLTLPRDA-----------EALKVLENATSRIRS-IAMNQEA----- 167

2vam_chainA_p001 114 -----------------GALTVGVVTRPFTF-----------EGRKRQLQAAGGISA-MKEAVD------ 148

2vap_chainA_p002 132 -----------------GALTVAVVTLPFVM-----------EGKVRMKNAMEGLER-LKQHTD------ 166

3cb2_chainA_p003 162 -----------------KKLVQTYSVFPNQDEM--------SDVVVQPYNSLLTLKR-LTQNAD------ 199

[Consensus_aa:](http://prodata.swmed.edu/promals3d/info/consensus.html) ....................*h*..*hhhh***P**.....................**N**t...*l*.c.*l*...........

[Consensus_ss:](http://prodata.swmed.edu/promals3d/info/consensus_ss.html) eeeeeee hhhhhhhhhhhh hhhh

Conservation:

gi_14318481 198 ------ETFCIDNEALYDICQRT---LKLNQPSYGDLNNLVS-------SVMSGVT---TSLRYP----- 243

gi_18568139 198 ------ESMCIDNEALYDICFRT---LKLTTPTFGDLNHLVS-------AVVSGVT---CCLRFP----- 243

gi_6323554 201 ------CTFMVDNEAIYDMCKRN---LDIPRPSFANLNNLIA-------QVVSSVT---ASLRFD----- 246

gi_15241179 200 ------VAVLLDNEAIYDICRRS---LDIERPTYTNLNRLIS-------QIISSLT---TSLRFD----- 245

gi_71397525 200 ------VAAMLDNEAIYDLTRRN---LDIERPTYTNLNRLIG-------QVVSALT---ASLRFD----- 245

gi_6323241 200 ------ATVVFDNASLLNISGKV---FRNPNIDLQHTNQLIS-------TIISSVT---NSIRFP----- 245

gi_71652627 203 ------CVVVLDNTALNRIATDN---LHISSPTVEQMNGLVS-------TVMAAST---ATLRYP----- 248

gi_15233174 201 ------CVVVLDNTALGRIAVER---LHLTNPTFAQTNSLVS-------TVMSAST---TTLRYP----- 246

gi_190016319 201 ------CLVVLDNTALNRIATDR---LHIQNPSFSQINQLVS-------TIMSAST---TTLRYP----- 246

gi_161528770 142 ------CTVVLDNDSLLESNPD----LTPK-ACYDIANSAIM-------HVVESLG---TSEMSHD---- 186

gi_118194654 140 ------CTIVIDNDAFLGANPG----MSPA-ECHGMTDSAVL-------YMAGSLG---SLPEGT----- 183

gi_20089831 173 ------DGIILADNQYLKNIGG-----SIQ-EAYDGINDMIA-------ERILFLL---DALDSE----- 215

gi_14521022 157 ------SIIAIDNNKLKESD------EDIS-QAYERINYTIV-------ERIASLL---ALIDVP----- 198

gi_240102830 157 ------SIIAIDNNKLKEGD------LDIS-QAYEMINYTIV-------ERIASLL---ALIDVP----- 198

gi_15789554 159 ------STLLVDNDAWRSTG------DSVT-EAFDEINSRIA-------RRVGILLAAGENIEGV----- 203

gi_257052276 159 ------ATLLIDNDAWQSAD------ESVE-EGFETINQQIA-------QRVGLLLASGEVIDGV----- 203

gi_126178676 162 ------NTFIFDNSAWKNEG------ESVK-DAYNRLNDEIVRRFGVLFRAGEVG----KAGV------- 207

gi_11498814 165 ------NLILVDNGAWKFEG------TSLK-ESFAKINEEIVRRLALLARAGEPIE---EDVV------- 211

gi_170290506 154 ------VTVVNANDLAMEKAKS----VDLN-YAFSMVNRKIE-------RSIYALV---KMQSSET---G 199

gi_182414456 151 ------AVIPLPNDVLLQESAEN---ETVL-DSFARADEWIG-------RGVKSIW---AMLFKT----- 195

gi_41615257 154 ------TTVILDNNKLFEVARG----LNVQ-QAFALSNELVA-------QTVSGVV---EIVTGA----- 197

gi_14521492 159 ------TVIIIQNDKLKELVPK----LPIQ-IAFRFADEIIA-------RMVKGIV---ETIKLP----- 202

gi_16082526 151 ------AAIVIPNDKLIEKYND----VPVY-KAFKFEDEVIS-------TGIKGIT---DLIMNT----- 194

gi_170290956 151 ------TIVVVNNDKILEIAKE----LPLY-QAFFISDEIVA-------RAVKGVV---ELVVKP----- 194

gi_41614929 151 ------THIVIPNDKLLEIAPN----LPIA-VAFKLSDEVLA-------NTIKKTT---ELILKP----- 194

gi_11498178 150 ------TVVVLDNNKLLDYYPN----LPID-AAFSVMDQLIA-------ETIKGIS---DTITIP----- 193

gi_15668803 150 ------TVIIIDNNKLLDLVPN----LPIN-DAFKVADEIIA-------QAVKGIT---ETIAVP----- 193

gi_15789500 150 ------SIIVLDNNRLLDYVPN----LPIG-KAFSVMDQIIA-------ETVKGIS---ETITQP----- 193

gi_20092672 150 ------TVIVLDNNRLLNYVPN----LPID-QAFSVMDQLIA-------ETVKGIT---ETITVP----- 193

gi_126179395 150 ------SVIVLDNNRLIKYVPN----LPLG-QAFSVMDQLIA-------ETVKGIS---ETITEP----- 193

gi_15679670 151 ------TVIVIPNDKLLEVAPN----LPLN-KAFMVADEILG-------RAVKGIT---ELITKP----- 194

gi_11498146 151 ------TVIVIPNDRLLEVVPN----YPMQ-LAFKVADEILM-------RAVKGIT---ELITKP----- 194

gi_15789633 151 ------TVIVVPNDRLLDSVGK----LPVR-EAFKVSDEVLM-------RSVKGIT---ELITKP----- 194

gi_126178359 151 ------TVIVVPNDRLLEVVPR----LPLH-AAFKVSDEVLM-------RAVKGIT---ELITMP----- 194

gi_20093060 151 ------TVIVVPNDKLIEVVPR----LPLQ-AAFKVSDEVLM-------RAVKGIT---ELITKP----- 194

gi_14520222 151 ------TVIVIPNDKLLEVAPK----LPIQ-MAFKVADEILV-------QAVKGIT---ELITKP----- 194

gi_15668546 151 ------TLVVIPNEKLFEIVPN----MPLK-LAFKVADEVLI-------NAVKGLV---ELITKD----- 194

gi_53711593 152 ------ALLVINNERLREIYSD----LTFM-NAFGKADDTLS-------IAAKSIA---EIITMR----- 195

gi_170291053 152 ------TVVLISNDKLLKLAGD----RPLD-EAFMIADMTLA-------VMVKGIA---EIIRKR----- 195

gi_15605992 151 ------AYIVIHNDKIKELSNRT---LTIK-DAFKEVDSVLS-------KAVRGIT---SIVVTP----- 195

gi_16128088 151 ------SLITIPNDKLLKVLGRG---ISLL-DAFGAANDVLK-------GAVQGIA---ELITRP----- 195

gi_15643599 151 ------TLIKISNNKLMEELPRD---VKIK-DAFLKADETLH-------QGVKGIS---ELITKR----- 195

gi_15639381 151 ------TVIVIPNQNLLSVVDKR---CPIK-ETYLVADDLLR-------KSVQSIS---DLITLP----- 195

gi_55981058 151 ------AMVVVQNDRLLSAVDKK---MTLK-DAFLIADRVLY-------HGVKGIT---DVINLP----- 195

gi_16330088 151 ------TLIVIPNNQLLSVIPAE---TPLQ-EAFRVADDILR-------QGVQGIS---DIIIIP----- 195

gi_15609287 151 ------TLIVIPNDRLLQMGDAA---VSLM-DAFRSADEVLL-------NGVQGIT---DLITTP----- 195

gi_255767353 151 ------TLIVIPNDRILEIVDKN---TPML-EAFREADNVLR-------QGVQGIS---DLIATP----- 195

gi_73669953 ----------------------------------------------------------------------

gi_20091535 ----------------------------------------------------------------------

gi_14521848 ----------------------------------------------------------------------

gi_257053119 ----------------------------------------------------------------------

gi_284164354 ----------------------------------------------------------------------

gi_88602022 187 --------------------------ITDI-STATFTKRYFA-------NSFAALE---EVKVLA----- 214

gi_268325165 ----------------------------------------------------------------------

gi_269956178 ----------------------------------------------------------------------

gi_227497172 ----------------------------------------------------------------------

gi_240169446 ----------------------------------------------------------------------

gi_19553917 ----------------------------------------------------------------------

gi_182437626 ----------------------------------------------------------------------

gi_83312057 ----------------------------------------------------------------------

gi_160892492 ----------------------------------------------------------------------

gi_163815161 ----------------------------------------------------------------------

gi_189219502 ----------------------------------------------------------------------

gi_121606151 ----------------------------------------------------------------------

gi_154496732 ----------------------------------------------------------------------

gi_238059621 ----------------------------------------------------------------------

gi_15805565 ----------------------------------------------------------------------

gi_226358106 ----------------------------------------------------------------------

gi_149916637 ----------------------------------------------------------------------

gi_32476397 ----------------------------------------------------------------------

gi_168704658 ----------------------------------------------------------------------

gi_168700390 ----------------------------------------------------------------------

gi_87309437 ----------------------------------------------------------------------

gi_283778279 ----------------------------------------------------------------------

gi_52141958 ----------------------------------------------------------------------

gi_154500451 ----------------------------------------------------------------------

gi_153954291 ----------------------------------------------------------------------

gi_226315300 ----------------------------------------------------------------------

gi_193212596 ----------------------------------------------------------------------

gi_75908069 ----------------------------------------------------------------------

gi_37520551 ----------------------------------------------------------------------

gi_284051667 ----------------------------------------------------------------------

gi_22298564 ----------------------------------------------------------------------

gi_254410688 ----------------------------------------------------------------------

gi_153807774 ----------------------------------------------------------------------

gi_219848227 ----------------------------------------------------------------------

gi_17229535 ----------------------------------------------------------------------

gi_227993086 ----------------------------------------------------------------------

gi_90409221 ----------------------------------------------------------------------

gi_34499290 ----------------------------------------------------------------------

gi_152997985 ----------------------------------------------------------------------

gi_87119891 ----------------------------------------------------------------------

gi_226941044 ----------------------------------------------------------------------

gi_192360015 ----------------------------------------------------------------------

gi_146308213 ----------------------------------------------------------------------

gi_109896728 ----------------------------------------------------------------------

gi_77457477 ----------------------------------------------------------------------

gi_70728682 ----------------------------------------------------------------------

gi_161598655 185 DIKPLANVIVNDNTQMQRIIE------SQK-GTKDLKNRYVNWKE-VANDNVVSTL---HEINIIPENYG 243

gi_208703287 184 EAKPLANVILNYNPQMEKIVK------SQK-GTKDLKNQHMIWQN-VANDYVASTI---HEINIIPGNFG 242

gi_169825600 168 ----LGPILLVDNEKLFKQFSEEKPNSSVS-EYLRFSNKYVA-------EALHDLN---TVTASYL---P 219

gi_227811490 168 ----FGSIVLIDNAKLYRKFEEENPSALAN-EYTSYSNKYIA-------DALHEI----NLVTSSFT--P 219

gi_225871538 168 ----FGSIVLIDNAKLYRKFEEENPSALAN-EYTSYSNKYIA-------DALHEI----NLVTSSFT--P 219

2vam_chainA_p001 149 ------TLIVIPNDRILEIVDKN---TPML-EAFREADNVLR-------QGVQGIS---DLIATP----- 193

2vap_chainA_p002 167 ------TLVVIPNEKLFEIVPN----MPLK-LAFKVADEVLI-------NAVKGLV---ELITKD----- 210

3cb2_chainA_p003 200 ------CLVVLDNTALNRIATDR---LHIQNPSFSQINQLVS-------TIMSAST---TTLRYP----- 245

[Consensus_aa:](http://prodata.swmed.edu/promals3d/info/consensus.html) ......................................................................

[Consensus_ss:](http://prodata.swmed.edu/promals3d/info/consensus_ss.html)

Conservation:

gi_14318481 244 -GQLNSDLRKLAVNLVPFPRLHFFMVGYAPLTA------------------------------------- 275

gi_18568139 244 -GQLNSDLRKLAVNLVPFPRLHFFMMGFAPLTS------------------------------------- 275

gi_6323554 247 -GSLNVDLNEFQTNLVPYPRIHFPLVSYSPVLS------------------------------------- 278

gi_15241179 246 -GAINVDITEFQTNLVPYPRIHFMLSSYAPVIS------------------------------------- 277

gi_71397525 246 -GALNVDLTEFQTNLVPYPRIHFVLTSYAPVIS------------------------------------- 277

gi_6323241 246 -SYMYSSMSSIYSTLIPSPELHFLSPSFTPFTS------------------------------------- 277

gi_71652627 249 -GYMNNDLMSMLASLIPTPRCHFICTGYTPTTL------------------------------------- 280

gi_15233174 247 -GYMNNDLVGLLASLIPTPRCHFLMTGYTPLTV------------------------------------- 278

gi_190016319 247 -GYMNNDLIGLIASLIPTPRLHFLMTGYTPLTT------------------------------------- 278

gi_161528770 187 TNILTTSKE------------------------------------------------------------- 195

gi_118194654 184 -AVASTSRD------------------------------------------------------------- 191

gi_20089831 216 -MMMVTDLGDFQTVMSGGAGLATMGFFRA----------------------------------------- 243

gi_14521022 199 -GEQTLDASDLKFVLRAMGSFATVGYAKA----------------------------------------- 226

gi_240102830 199 -GEQTLDASDLKFVLKAFGSFATVGYAKA----------------------------------------- 226

gi_15789554 204 -GESVVDSSEVINTLQSGE-MSAIGFASAEA--------------------------------------- 232

gi_257052276 204 -GESVVDSSEVINTLRPGG-IAALGYASAKA--------------------------------------- 232

gi_126178676 208 -GEMVVDSSEVINTLRGGG-ISTVGYAISEKIS------------------------------------- 238

gi_11498814 212 -GEMVVDSSEVVNTLRGGG-ISSIGYATTLAES------------------------------------- 242

gi_170290506 200 PGYVNVDLSNFARISYQSG-LGFIGVGRG----------------------------------------- 227

gi_182414456 196 -GLINLDFAGLQQVFAQRGGKTLFGLGEG----------------------------------------- 223

gi_41615257 198 -ADINRDLADIKAIMEEGH-VAAIGIGES----------------------------------------- 224

gi_14521492 203 -SMVNIDYADIYSVMKGGG-PALIGIGES----------------------------------------- 229

gi_16082526 195 -GTINLDFNDLRKVMKDAG-YAAIGMGSS----------------------------------------- 221

gi_170290956 195 -GLVNVDLADLRNVIESGG-PAVLTFGES----------------------------------------- 221

gi_41614929 195 -GQVTRDFADLKVILENGG-LGMVGFGES----------------------------------------- 221

gi_11498178 194 -SLVNIDFADVKAIMGHGG-VAVMLVGEA----------------------------------------- 220

gi_15668803 194 -SLINIDFADVKAVMSGGG-VAMIGVGEVD---------------------------------------- 221

gi_15789500 194 -SLINLDYADMTAIMNQGG-VAVMLVGET----------------------------------------- 220

gi_20092672 194 -SLINLDYADIRTIMSCGG-VAVMLVGES----------------------------------------- 220

gi_126179395 194 -SLINIDYADVRAIMSKGG-VAVMLVGES----------------------------------------- 220

gi_15679670 195 -GLVSLDFADVRSIMKGSG-MAMIGMGEA----------------------------------------- 221

gi_11498146 195 -ALINLDFADVRTVMEKGG-VAMIGLGEA----------------------------------------- 221

gi_15789633 195 -GLVNLDFADVRTVMEKGG-VAMIGLGEA----------------------------------------- 221

gi_126178359 195 -GLVNLDFADVRTVMERGG-VAMIGMGES----------------------------------------- 221

gi_20093060 195 -GLVNLDFADIRTVMQNGG-VAMIGLGES----------------------------------------- 221

gi_14520222 195 -GLVNLDFNDVRAVMKDGG-VAMIGIGES----------------------------------------- 221

gi_15668546 195 -GLINVDFADVKAVMNNGG-LAMIGIGES----------------------------------------- 221

gi_53711593 196 -GTVNLDFADVKTILKDGG-VAIMSTGFG----------------------------------------- 222

gi_170291053 196 -TMVNVDLNDIRTLMSVGG-VAAVGIGE------------------------------------------ 221

gi_15605992 196 -AVINVDFADVRTTLEEGG-LSIIGMGEG----------------------------------------- 222

gi_16128088 196 -GLMNVDFADVRTVMSEMG-YAMMGSGVA----------------------------------------- 222

gi_15643599 196 -GYINLDFADIESVMKDAG-AAILGIGVG----------------------------------------- 222

gi_15639381 196 -GEVNLDFMDVKNTMEGQG-YALIGVGEG----------------------------------------- 222

gi_55981058 196 -GLINVDFADVKALLEGAG-QVLMGIGAG----------------------------------------- 222

gi_16330088 196 -GLVNVDFADVRAVMADAG-SALMGIGVG----------------------------------------- 222

gi_15609287 196 -GLINVDFADVKGIMSGAG-TALMGIGSA----------------------------------------- 222

gi_255767353 196 -GLINLDFADVKTIMSNKG-SALMGIGIA----------------------------------------- 222

gi_73669953 ----------------------------------------------------------------------

gi_20091535 ----------------------------------------------------------------------

gi_14521848 ----------------------------------------------------------------------

gi_257053119 ----------------------------------------------------------------------

gi_284164354 ----------------------------------------------------------------------

gi_88602022 215 -A-------------------------------------------------------------------- 215

gi_268325165 ----------------------------------------------------------------------

gi_269956178 ----------------------------------------------------------------------

gi_227497172 ----------------------------------------------------------------------

gi_240169446 ----------------------------------------------------------------------

gi_19553917 ----------------------------------------------------------------------

gi_182437626 ----------------------------------------------------------------------

gi_83312057 ----------------------------------------------------------------------

gi_160892492 ----------------------------------------------------------------------

gi_163815161 ----------------------------------------------------------------------

gi_189219502 ----------------------------------------------------------------------

gi_121606151 ----------------------------------------------------------------------

gi_154496732 ----------------------------------------------------------------------

gi_238059621 ----------------------------------------------------------------------

gi_15805565 ----------------------------------------------------------------------

gi_226358106 ----------------------------------------------------------------------

gi_149916637 ----------------------------------------------------------------------

gi_32476397 ----------------------------------------------------------------------

gi_168704658 ----------------------------------------------------------------------

gi_168700390 ----------------------------------------------------------------------

gi_87309437 ----------------------------------------------------------------------

gi_283778279 ----------------------------------------------------------------------

gi_52141958 ----------------------------------------------------------------------

gi_154500451 ----------------------------------------------------------------------

gi_153954291 ----------------------------------------------------------------------

gi_226315300 ----------------------------------------------------------------------

gi_193212596 ----------------------------------------------------------------------

gi_75908069 ----------------------------------------------------------------------

gi_37520551 ----------------------------------------------------------------------

gi_284051667 ----------------------------------------------------------------------

gi_22298564 ----------------------------------------------------------------------

gi_254410688 ----------------------------------------------------------------------

gi_153807774 ----------------------------------------------------------------------

gi_219848227 ----------------------------------------------------------------------

gi_17229535 ----------------------------------------------------------------------

gi_227993086 ----------------------------------------------------------------------

gi_90409221 267 -FSYNVDREDHES--------------------------------------------------------- 278

gi_34499290 266 -FSYNFDNEEAGA--------------------------------------------------------- 277

gi_152997985 265 NFSYNEEREDDEV--------------------------------------------------------- 277

gi_87119891 266 -FSYNVEREDHDH--------------------------------------------------------- 277

gi_226941044 269 -FSYNFDNEEANT--------------------------------------------------------- 280

gi_192360015 266 -FSYNFDNEDSSD--------------------------------------------------------- 277

gi_146308213 266 -FSYNFDNEQSSA--------------------------------------------------------- 277

gi_109896728 265 DFSYNFDKEDSSD--------------------------------------------------------- 277

gi_77457477 271 -FSYNFANEDSSE--------------------------------------------------------- 282

gi_70728682 265 -FSYNFDNEDSSE--------------------------------------------------------- 276

gi_161598655 244 SDNVTYDPSDLIKLLSIPGRFLTIGKARIAKFDLHSLENSIKRSLDEGFFSAEHQFETATMYGGFVLRPS 313

gi_208703287 243 SDQVTYDPSDLQKLFSISGKFLTIGKARIKKQDLHSLESSIQKSLNDNYFTCGHKFETAKTFANILLRPT 312

gi_169825600 220 TGEYHFDSSEFEKLLKTPG-LLHFARFTEKASSIDSSNNLSYAQKLKELIQKGVLSDGYNLEEAQRLAVS 288

gi_227811490 220 FSDTHFDASEFAQVINTPG-VLSLAKLELKSNQLDTENPLGYLTQLGNALEKGVLYDTEREELESAKKSA 288

gi_225871538 220 FSDTHFDASEFAQVINTPG-VLSLAKLELKSNQLDTENPLGYLTQLGNALEKGVLYDTEREELESAKKSA 288

2vam_chainA_p001 194 -GLINLDFADVKTIMSNKG-SALMGIGIATGENRAAEAAKKAISSPLLEAAIDGAQGVLMNITGGTNLSL 261

2vap_chainA_p002 211 -GLINVDFADVKAVMNNGG-LAMIGIGESDSEKRAKEAVSMALNSPLLDVDIDGATGALIHVMGPEDLTL 278

3cb2_chainA_p003 246 -GYMNNDLIGLIASLIPTPRLHFLMTGYTPLTSVRKTTVLDVMRRLLQPKNVMVSTGRDTNHCYIAILNI 314

[Consensus_aa:](http://prodata.swmed.edu/promals3d/info/consensus.html) ......................................................................

[Consensus_ss:](http://prodata.swmed.edu/promals3d/info/consensus_ss.html)

Conservation:

gi_14318481 ----------------------------------------------------------------------

gi_18568139 ----------------------------------------------------------------------

gi_6323554 ----------------------------------------------------------------------

gi_15241179 ----------------------------------------------------------------------

gi_71397525 ----------------------------------------------------------------------

gi_6323241 ----------------------------------------------------------------------

gi_71652627 ----------------------------------------------------------------------

gi_15233174 ----------------------------------------------------------------------

gi_190016319 ----------------------------------------------------------------------

gi_161528770 ----------------------------------------------------------------------

gi_118194654 ----------------------------------------------------------------------

gi_20089831 ----------------------------------------------------------------------

gi_14521022 ----------------------------------------------------------------------

gi_240102830 ----------------------------------------------------------------------

gi_15789554 ----------------------------------------------------------------------

gi_257052276 ----------------------------------------------------------------------

gi_126178676 ----------------------------------------------------------------------

gi_11498814 ----------------------------------------------------------------------

gi_170290506 ----------------------------------------------------------------------

gi_182414456 ----------------------------------------------------------------------

gi_41615257 ----------------------------------------------------------------------

gi_14521492 ----------------------------------------------------------------------

gi_16082526 ----------------------------------------------------------------------

gi_170290956 ----------------------------------------------------------------------

gi_41614929 ----------------------------------------------------------------------

gi_11498178 ----------------------------------------------------------------------

gi_15668803 ----------------------------------------------------------------------

gi_15789500 ----------------------------------------------------------------------

gi_20092672 ----------------------------------------------------------------------

gi_126179395 ----------------------------------------------------------------------

gi_15679670 ----------------------------------------------------------------------

gi_11498146 ----------------------------------------------------------------------

gi_15789633 ----------------------------------------------------------------------

gi_126178359 ----------------------------------------------------------------------

gi_20093060 ----------------------------------------------------------------------

gi_14520222 ----------------------------------------------------------------------

gi_15668546 ----------------------------------------------------------------------

gi_53711593 ----------------------------------------------------------------------

gi_170291053 ----------------------------------------------------------------------

gi_15605992 ----------------------------------------------------------------------

gi_16128088 ----------------------------------------------------------------------

gi_15643599 ----------------------------------------------------------------------

gi_15639381 ----------------------------------------------------------------------

gi_55981058 ----------------------------------------------------------------------

gi_16330088 ----------------------------------------------------------------------

gi_15609287 ----------------------------------------------------------------------

gi_255767353 ----------------------------------------------------------------------

gi_73669953 ----------------------------------------------------------------------

gi_20091535 ----------------------------------------------------------------------

gi_14521848 ----------------------------------------------------------------------

gi_257053119 ----------------------------------------------------------------------

gi_284164354 ----------------------------------------------------------------------

gi_88602022 ----------------------------------------------------------------------

gi_268325165 ----------------------------------------------------------------------

gi_269956178 ----------------------------------------------------------------------

gi_227497172 ----------------------------------------------------------------------

gi_240169446 ----------------------------------------------------------------------

gi_19553917 ----------------------------------------------------------------------

gi_182437626 ----------------------------------------------------------------------

gi_83312057 ----------------------------------------------------------------------

gi_160892492 ----------------------------------------------------------------------

gi_163815161 ----------------------------------------------------------------------

gi_189219502 ----------------------------------------------------------------------

gi_121606151 ----------------------------------------------------------------------

gi_154496732 ----------------------------------------------------------------------

gi_238059621 ----------------------------------------------------------------------

gi_15805565 ----------------------------------------------------------------------

gi_226358106 ----------------------------------------------------------------------

gi_149916637 ----------------------------------------------------------------------

gi_32476397 ----------------------------------------------------------------------

gi_168704658 ----------------------------------------------------------------------

gi_168700390 ----------------------------------------------------------------------

gi_87309437 ----------------------------------------------------------------------

gi_283778279 ----------------------------------------------------------------------

gi_52141958 ----------------------------------------------------------------------

gi_154500451 ----------------------------------------------------------------------

gi_153954291 ----------------------------------------------------------------------

gi_226315300 ----------------------------------------------------------------------

gi_193212596 ----------------------------------------------------------------------

gi_75908069 ----------------------------------------------------------------------

gi_37520551 ----------------------------------------------------------------------

gi_284051667 ----------------------------------------------------------------------

gi_22298564 ----------------------------------------------------------------------

gi_254410688 ----------------------------------------------------------------------

gi_153807774 ----------------------------------------------------------------------

gi_219848227 ----------------------------------------------------------------------

gi_17229535 ----------------------------------------------------------------------

gi_227993086 ----------------------------------------------------------------------

gi_90409221 ----------------------------------------------------------------------

gi_34499290 ----------------------------------------------------------------------

gi_152997985 ----------------------------------------------------------------------

gi_87119891 ----------------------------------------------------------------------

gi_226941044 ----------------------------------------------------------------------

gi_192360015 ----------------------------------------------------------------------

gi_146308213 ----------------------------------------------------------------------

gi_109896728 ----------------------------------------------------------------------

gi_77457477 ----------------------------------------------------------------------

gi_70728682 ----------------------------------------------------------------------

gi_161598655 314 NADFFKDVNTENRIRNTLGEYKRLDEIAGKFGDPIWDNEYAVCYTIFAGMTMPK---------------- 367

gi_208703287 313 NAGFFQDIETESKINKVLNEYTDIKRLSGKMANPMWDSEHAVNYAIFGGMNLPE---------------- 366

gi_169825600 289 VLTDNATAKRMFTFEFTKRMEDLVNELSPTALEKPIATYQSKDIKGPAEVSFYAVFAGLGLPQ------- 351

gi_227811490 289 LSIVTSPLRASRLYNFSFLNQMENFLKDRTPYVDERPIAPYVNKHTAKKEEDIVKFYSVVAGLPLPK--- 355

gi_225871538 289 LSIVTSPLRAGRLYNFSFLNQMENFLKDRTPYVDERPIAPYVNKHTAKKEEDIVKFYSVVAGLPLPK--- 355

2vam_chainA_p001 262 YEVQEAADIVASASDQDVNMIFGSVINENLKDEIVVTVIATGF--------------------------- 304

2vap_chainA_p002 279 EEAREVVATVSSRLDPNATIIWGATIDENLENTVRVLLVITGVQSRIEFTDTGLKRK------------- 335

3cb2_chainA_p003 315 IQGEVDPTQVHKSLQRIRERKLANFIPWGPASIQVALSRKSPYRVSGLMMANHTSISSLFERTCRQYDKL 384

[Consensus_aa:](http://prodata.swmed.edu/promals3d/info/consensus.html) ......................................................................

[Consensus_ss:](http://prodata.swmed.edu/promals3d/info/consensus_ss.html)

**B. FtsZ-like 1**

Conservation: 9 5 67 97 9

gi_160892492_ref_ZP_02073 1 MILIDTQKEE----FSKLRDIRVF----GRISHDDRF------NGNLLVIGLGGIGSRTVCNLKGM---- 52

gi_163815161_ref_ZP_02206 1 MILIDTQKEE----FSKLKDIRVF----GKISHENRF------NKNFLVIGLGGLGSRAVCALKGM---- 52

gi_153954291_ref_YP_00139 1 MN---SRIRE----HLQDLEVSRGGGIISDKIRIETI------PNPMLIIGLGGTGIDAMLRLKYQINKR 57

gi_226315300_ref_YP_00277 1 MK---AVVRE----HIQQLDVSLGGGIVSDKIRVDTI------DNPMLVIGLGGTGIDALLRLKYQVNRR 57

gi_219848227_ref_YP_00246 1 MT---TTLQ---PTQVY----------IPSALFIR----------PTLVIGLGGTGVDVIRQLKRR---- 40

gi_22298564_ref_NP_681811 1 ME---RATM---PAQVE-------------EKSIV----------PTVIVGVGGTGIEVLSRVRRL---- 37

gi_254410688_ref_ZP_05024 1 -----------MATPVE-------------EKSMV----------PTILIGIGGTGAEIVSRVRRL---- 32

gi_284051667_ref_ZP_06381 1 MV---NANVGQDNNNIQ-------------ENTIV----------PTLIIGVGGTGLEAMTRVRRL---- 40

gi_37520551_ref_NP_923928 1 MA------------MAE-------------YTGMT----------PTVVVGLGGTGKEILIKIRRM---- 31

gi_15805565_ref_NP_294261 1 MA---ENM------------------------RVF----------KTLVIGLGSTGTEILESLADR---- 29

gi_238059621_ref_ZP_04604 1 MWCAGPGSRCRRRTARPSPINPDHSPTADMEVTVL----------PALVIGLGGTGSIAAAHLKHR---- 56

gi_240169446_ref_ZP_04748 1 -------------------------------------------MRRFLIVGCGGSGGATLAYMMDQ---- 23

gi_227497172_ref_ZP_03927 1 -------------------------------------------MRKVLVVGCGGSGAKTLAYMMDQ---- 23

gi_269956178_ref_YP_00332 1 MLR------------------------------------------PFLLVGVGGSGGKTLRIIRED---- 24

gi_268325165_emb_CBH38753 1 MG---------------------------TGEDTV----------NRVAIGIGGQGSSIVNNILRT---- 29

gi_83312057_ref_YP_422321 1 MAA------------------------------------------TNLFLGFGGTGAHILTFLKEF---- 24

gi_17229535_ref_NP_486083 1 MS---RPT-----------------------VVFR----------PTVVIGLGGTGYEVALKLKKR---- 30

gi_154496732_ref_ZP_02035 1 MN---DTH----KKRVKDNLLTLD---YLEGTGVISIHKRKLVEELFIFISSGGNGHKSLCAVRKE---- 56

gi_189219502_ref_YP_00194 1 MNA------------------------------------------NHFIIGLGGTGGKVIRAFRKT---- 24

gi_121606151_ref_YP_98348 1 M--------------------------------------------NHFIIGLGGTGGKIIRALRKS---- 22

gi_75908069_ref_YP_322365 1 MN---QVSA----NELQ-------------YRGIN----------RTICIGLGGTGRDVLMRIRRL---- 36

gi_193212596_ref_YP_00199 1 MV---EELKVGKKTEVT-------------LTEVV----------PTLFIALGGTGAQVLWRIRRR---- 40

gi_149916637_ref_ZP_01905 1 MP-----------------------------GEVT----------PTLFVGLGGSGGRAVGRIAKR---- 27

gi_88602022_ref_YP_502200 1 MS---------DKDLFE-------------ESGII----------PTLAIGVGMGGVSVVKEFISF---- 34

gi_153807774_ref_ZP_01960 1 MA-----------------------------TKIK----------RCLYIGLGGTGMKSLLHTKKM---- 27

[Consensus_aa:](http://prodata.swmed.edu/promals3d/info/consensus.html) **M**................................*h*............*hlhl***G***l***GG**o**G**.p*hl*..*h*.c.....

[Consensus_ss:](http://prodata.swmed.edu/promals3d/info/consensus_ss.html) eeeeee hhhhhhhhhhh

Conservation: 66 58

gi_160892492_ref_ZP_02073 53 -------MVDDITP--------------------EDNIHFLMVDSD----IPEMEQTIEDS-------KE 84

gi_163815161_ref_ZP_02206 53 -------LVNDITP--------------------EDNINFLMIDSD----ISEMERTIEDS-------KE 84

gi_153954291_ref_YP_00139 58 FILEEDIISNTRKD-------------------KPKKVEFLGFETN----QGEKNKR-YPG-------NG 96

gi_226315300_ref_YP_00277 58 FKLPVDQLSKKRKE-------------------KPDNIEFIAFETN----EHDRNKKYK----------- 93

gi_219848227_ref_YP_00246 41 -------IRQSMQP-------------------MPGVLEFLVVDTE----MPQNMPGEE----------- 69

gi_22298564_ref_NP_681811 38 -------VEETYGS-----------------LKQFPVISFLAIDTD----RDYKVSNPLAA--------- 70

gi_254410688_ref_ZP_05024 33 -------VAETYGN-----------------LTNFPILSFLVIDTD----KDYKINNPDAA--------- 65

gi_284051667_ref_ZP_06381 41 -------IVESYGS-----------------LEKLPVVGFLHIDTD----EKPQVKKPEMA--------- 73

gi_37520551_ref_NP_923928 32 -------IVESYGT-----------------LDALPIVSFLHLDTE----QNAKVSEPQTV------LKQ 67

gi_15805565_ref_NP_294261 30 -------IDWEVGG-----------------LSRAPWVEFLAVETD----VAKPNRFNG----------- 60

gi_238059621_ref_ZP_04604 57 -------LATEQRWQDLRRDPGAPERTAGRPYDWPVLLRALDVDRR----TRPRVDGVSLD-VDTE---- 110

gi_240169446_ref_ZP_04748 24 -------LRSELHAAGIES--------------LLPGWQFVVIDVP----SGAEDGPEGLS-NVPAQGGT 67

gi_227497172_ref_ZP_03927 24 -------LHADLAAYGIEK--------------IPGCWQFLSVDTP----LQEEKPG-GLG-SVTQQGGA 66

gi_269956178_ref_YP_00332 25 -------LLRRLKQAGWEH------------DDLPAAWQMIHIDVP----NRADGDDVDLPAQLPDRQYK 71

gi_268325165_emb_CBH38753 30 -------LKHKTGK-------------------APRNEEFLIIDTD----PASA---------------- 53

gi_83312057_ref_YP_422321 25 -------TVYKHGS-------------------KPDGVMFLEFDTI----AGWK-PGQTV--DIAGGGGG 61

gi_17229535_ref_NP_486083 31 -------FIDVYGD-------------------VPDIIRFLSIDTT----ENIQSREKSP-------DGT 63

gi_154496732_ref_ZP_02035 57 -------LEWRVALQE-----------------LKDKVRFLAVDAA----YKELDELLE----------- 87

gi_189219502_ref_YP_00194 25 -------IFQEFRKVD----------------PPDIRLSYLYVDSS----DELMRLDDPS----WKVLGQ 63

gi_121606151_ref_YP_98348 23 -------LYQEFHGGP----------------PAGVGIGYLYVDSS----SEMMAMDDPT----WKTLGT 61

gi_75908069_ref_YP_322365 37 -------IVDRYGD-----------------LSNLPIVSFVHLDTD----KAATQVTGIRT--GSTYHGV 76

gi_193212596_ref_YP_00199 41 -------IINNLWGS-GTGQAV-----RIDNLTEFPFAEFLQIDLSAFETEQGKAEKNDIL-------SN 90

gi_149916637_ref_ZP_01905 28 -------LRAQQDFD----------------LQYRSLVRFVAIDTN----DADLARLRKGT--------- 61

gi_88602022_ref_YP_502200 35 --------VEKNGI--------------------IDNYRFVAIDSN----IDDLNRII------------ 60

gi_153807774_ref_ZP_01960 28 -------FIETYGE-------------------VPPMIGFLGIDTD----GGEYKKELDS-------KYG 60

[Consensus_aa:](http://prodata.swmed.edu/promals3d/info/consensus.html) .......*l*..p*h*.......................s.*h*p**F***l*.*l***D**ss........................

[Consensus_ss:](http://prodata.swmed.edu/promals3d/info/consensus_ss.html) hhhhh eeeeee

Conservation: 9

gi_160892492_ref_ZP_02073 85 --HIGF---------NALEV-LSIYRPN-IENILADGIK-----KNPVH--------PNLANWMDADFPD 128

gi_163815161_ref_ZP_02206 85 --GVGF---------NALEV-ISIYRPD-IENVLENGIQ-----NNKIH--------KNLANWMSPEFPE 128

gi_153954291_ref_YP_00139 97 --GVGL--------DPQSEL-VMLSNAE-IRSILKDR--------KILD--------DCIKEWLSPELSS 138

gi_226315300_ref_YP_00277 94 --GIGL--------DPVTEF-VLLSNPE-IGGVLQNR--------SILE--------PYITDWLSPELTI 135

gi_219848227_ref_YP_00246 70 --------------RILDREIAYIGDFN-AGKVLDH---------LDQH--------PHIKDWWPNGQV- 106

gi_22298564_ref_NP_681811 71 --GSPL--------KDNEKHWASVSGKN-VQQIIQN---------LDNY--------PWIASWFPRELER 112

gi_254410688_ref_ZP_05024 66 --GSPL--------KDNEKHWASVSGKQ-VRDMVSN---------MENY--------PWINRWFPRELER 107

gi_284051667_ref_ZP_06381 74 --GPPL--------EDYEKFWASVTFDE-AKKVKDN---------PTTY--------SWYYDWLPPELTP 115

gi_37520551_ref_NP_923928 68 --DISL--------RPVEQVWTKVED---AKAILSR---------IGSY--------PYLAEWFPSQLK- 106

gi_15805565_ref_NP_294261 61 ---------------TDDFKTLGVPATA-WRDMLNRPEL------YDAS--------IALNTWADLETLG 100

gi_238059621_ref_ZP_04604 111 --DLYL----------DGSVRSMIDKV--RQGRDVA---------QEFY--------PTVVPWFGPEDAR 149

gi_240169446_ref_ZP_04748 68 --YIGC--------GPQGSSYAILDAA--LSQRLAA---------NAAL--------DTIATWAPRHPEE 108

gi_227497172_ref_ZP_03927 67 --YVAC--------GVSAGSYSVVDDS--LTHQVQS---------KGPAG------LRQLATWMPGTVQD 109

gi_269956178_ref_YP_00332 72 --GLVA----------TGVDYTTIDTA--MKAKAG----------PSFV--------DAAATWRP-DPNS 108

gi_268325165_emb_CBH38753 54 ------------------NACSEIEER--KKIILNR---------PDKIL------MKNTNRWLPDP--- 85

gi_83312057_ref_YP_422321 62 EEVVAKGYEEANSLQPQAEYFQLMDRHPSLRDLVTHHLSPA--GNPGDY--------PQYRDWLHSQWLS 121

gi_17229535_ref_NP_486083 64 --KVVL----------EPNELYAISVAN-PLPL-------------TRN--------DHIDEWWPRNIPT 99

gi_154496732_ref_ZP_02035 88 --KYGF----------DSTEVLKLPFEG-AHESINP---------DTIS--------PQMKEWVDPELY- 126

gi_189219502_ref_YP_00194 64 --SVQI--------PPTSQLLIKSSN---LAAILDD---------LPSY--------PGIQPWIGDKNVW 103

gi_121606151_ref_YP_98348 62 --SVQL--------SKASQLLITDAN---LTSRLDN---------LDSY--------PGLKHWLGSPQEW 101

gi_75908069_ref_YP_322365 77 --DLSF--------REAEKVSATMSAKE-VTMFVEGLERRSEYTRYGPY--------DHIARWFPPQLLR 127

gi_193212596_ref_YP_00199 91 --KIKF--------KESE---RLVKKLD-LNQYIKSEEA------LDCY--------PLIREWFPLSRKT 132

gi_149916637_ref_ZP_01905 62 --QEFG--------KVDETVLISDFDKV-AYSKLRR---------GESFADADDYFTQWVHDWYNFRE-- 109

gi_88602022_ref_YP_502200 61 ---EFA--------PNTSKIAITDHQ----YDVMNL---------KKNC--------PYLHKWVVMQKG- 97

gi_153807774_ref_ZP_01960 61 --NVVL--------TPNEQLPIRVDE---ARPIYEV---------N-----------KEHFAWLPEENIY 97

[Consensus_aa:](http://prodata.swmed.edu/promals3d/info/consensus.html) .....*h*............p.*h*..*h*.....*h*...*h*p........................*h*.p**W***h*s.p...

[Consensus_ss:](http://prodata.swmed.edu/promals3d/info/consensus_ss.html) ee h hhhhhh hhhhhh

Conservation: 76 6 9 89

gi_160892492_ref_ZP_02073 129 V-T---VTKD---------GAHGNRQIGRLMFSN--AYE-DIRMLLFDRLEEIHDKAN------------ 170

gi_163815161_ref_ZP_02206 129 I-I---IGRD---------GAKGNRQIGRLMFSN--AYT-DIRMLLFDKLQAVYDKTE------------ 170

gi_153954291_ref_YP_00139 139 E-----SGTD---------GAGGVRQVGRLLLFT--KIN-EIVDCIEKKIRLLQEDK------------- 178

gi_226315300_ref_YP_00277 136 T-----DGIS---------GASGVRQAGRLLLFT--KIT-QVVQTIEKKVKMLCEGT------------- 175

gi_219848227_ref_YP_00246 107 --VTG-SIFR---------GARQRRLVGRLSLYA--RWG-QFAHRLDTKLDKIRQIAE-NEQVEK----- 155

gi_22298564_ref_NP_681811 113 --NMT-SLEA---------GAGQIRACGRFAFFC--NYH-GIQQKFQAASDRVKGHES--FMQSR----- 160

gi_254410688_ref_ZP_05024 108 --NIT-SLEA---------GAGQIRACGRFALWC--NYH-EIRDKFLAACQRVKGREN--FMLDR----- 155

gi_284051667_ref_ZP_06381 116 --QNL-VSEQ---------GAGQIRACGRFAFFY--NHE-KIRNKCQQAITRITVGRN--QLTID----- 163

gi_37520551_ref_NP_923928 107 --GTD-SILA---------GAGQIRALGRFAFAV--NYQ-QVKGAFAAARGRLRGHEK--FMLDT----- 154

gi_15805565_ref_NP_294261 101 Q-LPAQSIDS---------GAGHIRMVGRLALLYPPNYN-EIKNAISQRVARLRNLTE--AQAKAALNKD 157

gi_238059621_ref_ZP_04604 150 EIRDDEALSFL------VEGAGQIRSFGRLAFFSDVLGPTPVTKRLEDALDQLTLVDA------------ 201

gi_240169446_ref_ZP_04748 109 --VTN-PIST---------GAGQYRAIGRMIVLS--KAG-EIRSRLQAAWDQLFRVET--ISAMSTAQ-- 159

gi_227497172_ref_ZP_03927 110 --VPF-PVTV---------GAGQCRGIGRLLILD--RLS-AVSQAVQDALARMASTQS--VSEAAEVA-- 160

gi_269956178_ref_YP_00332 109 --VNV-SPSK---------GAGQYRTLGRIITIA--GLK-RIDDAVQRARAALTGTDV--VGEMQEVS-- 159

gi_268325165_emb_CBH38753 86 --YLT-AAGA---------GCGQHRIYGRAMYNV--HRE-RIFSAIGAAAAELRNRTG------------ 128

gi_83312057_ref_YP_422321 122 T-VMPPSVLNI------TAGSAQQRQIGRFSMFT--NAE-KIIAQLSKSLRELSRAAA------------ 169

gi_17229535_ref_NP_486083 100 S-----SLIS---------GAGQIRARGRLAFFA--KVG-DINGLISQAINTVREIRS--SKQAFLDK-- 148

gi_154496732_ref_ZP_02035 127 EVTGGKAITMSSQSGFDSSGTAAWRQPGRVRLSQPNTIA-VLTTALTNAINSLLKGKP------------ 183

gi_189219502_ref_YP_00194 104 K-DIL-NSIVG------DAKGGQKRRLGRFLFAS--HAD-EFCSKLTHLVNEMQKGG------------- 149

gi_121606151_ref_YP_98348 102 R-DIL-NSIVG------ATLGGQKRRLGRFLFAC--KAD-KYREQVQTQVKLLQQSG------------- 147

gi_75908069_ref_YP_322365 128 --NIK-AVEE---------GAKGIRPVGRLAFFH--NYQ-KIKIAIETAERLSRGHDA---LLLR----- 174

gi_193212596_ref_YP_00199 133 I-NELNIDPE--------KGAGQIRALSRLFFFD--KYQ-EIKGAIRTKCDSLLDNVKSGTAQKR----- 185

gi_149916637_ref_ZP_01905 110 ------ESGA---------GAGQIRIESRLSLNRSIETG-DMVAKLQALANDLRSHA------------- 150

gi_88602022_ref_YP_502200 98 -------------------GALQERVYGRFLLDL--HKE-EITRTITAHIHDLSNLWK------------ 133

gi_153807774_ref_ZP_01960 98 --ALT-SMTL---------GAGQIRSNGRFAITV--NHT-EVENKIVSVLTDITRANI--SNN------- 143

[Consensus_aa:](http://prodata.swmed.edu/promals3d/info/consensus.html) ......s............**G**tt..**R**.*h***GR***hhh*....p*h*..p*l*...*l*...*h*..*l*.................

[Consensus_ss:](http://prodata.swmed.edu/promals3d/info/consensus_ss.html) h hhhhhhh hhh hhhhhhhhhhhhhhhhhh

Conservation: 6 5 6 5997768 57 6

gi_160892492_ref_ZP_02073 171 --------------------GNWMDVIIVSSLSGGTGSGILSDLAYNIRAYGKAK-KWAN---LRIGGCL 216

gi_163815161_ref_ZP_02206 171 --------------------EGTVDVLIVSGVSGGTGSGILSDVAYNIRAYGKAK-KWNN---LRIGGCL 216

gi_153954291_ref_YP_00139 179 --------------------EETLHVFILSGLSGGTGSGTFIDIAYIVRGIMNNIYGSKGDDKVNIMGYL 228

gi_226315300_ref_YP_00277 176 --------------------NKKLTVFLLSGLSGGTGSGCFLDIAYIVRGILERDFGSAGVDKVNTLGYL 225

gi_219848227_ref_YP_00246 156 -----------QGIRTE--RTGQVRVYIVSSLCGGTGSGLLLDVAFRVRSKLGDD--------GEICGVF 204

gi_22298564_ref_NP_681811 161 -----------YGLKVN---NSSLNVFITGSLSGGTGSGMLIDLGYCVRHWLRGQ-SS-----PLVTAIV 210

gi_254410688_ref_ZP_05024 156 -----------YGIKVS---TNAINVFITGSLSGGTGSGMLIDMGYSIRKWLQGE-GS-----PLITAIV 205

gi_284051667_ref_ZP_06381 164 -----------GDVLTV---EPKLNIFVVGSISGGTGSGMLIDLGYCLRNWFQGQ-R------LETTAII 212

gi_37520551_ref_NP_923928 155 -----------WKVQL----DQGINIFVVGSLSGGTGSGMLLDLAYNLRDWVPPS-DL-----PQSSAYL 203

gi_15805565_ref_NP_294261 158 NAGLEMSVQFAVNQASG---QTGVRVIVVGTLCGGTCSGTASDVGILLRTILSEE-EK-----TLGMFTL 218

gi_238059621_ref_ZP_04604 202 --------------------DSMPNIYVVSSTAGGTGAGILLDVLAYLQKLRQEH-GG----GFTVTLFC 246

gi_240169446_ref_ZP_04748 160 --------VPGIGQFDP---HQPPLVLVVSSMAGGAGASMALDVCRLLTLVSGLD-P------RLMGLFL 211

gi_227497172_ref_ZP_03927 161 --------RRVPGVGEPPATTAPPMVLVVSSMAGGSGASMTLDVCRVIAGTQTTP-AIDP---QLISVFL 218

gi_269956178_ref_YP_00332 160 --------RLLGGRAHA--SIGDPTVIVITSIAGGTGAGSAIDVCDVIRALPDKW-A------NDSVGFL 212

gi_268325165_emb_CBH38753 129 --------------------GRDFLILMVCAFGGGTGSSMLLDIAIDIRDWISKQ-FGA---DPVMFGIG 174

gi_83312057_ref_YP_422321 170 --------------------GGGINVWVIGSAAGGTGAGCMLDAGYLARLAAKQA-GNI---PITLLTAV 215

gi_17229535_ref_NP_486083 149 -----------FQVSN----RDGVEVFIVGSLAGGTGSGTFLDVAFLARQYLNSF--------SNVTGLF 195

gi_154496732_ref_ZP_02035 184 -------------------AGMRLNIIFLGGLAGGTSGGTMVDLPFLTRQIVRNI-SVARYKNTGVSAYL 233

gi_189219502_ref_YP_00194 150 --------------------QQNVTFHVCCGLAGGTGSGTLIDCICQIRKNYPDP-LG-----YRIILYT 193

gi_121606151_ref_YP_98348 148 --------------------ETDVTFHIVVGLAGGTGSGSVIDAVAQLRDLYPDS-KR-----FRILIYA 191

gi_75908069_ref_YP_322365 175 -----------KGLRV----EPGLNIFVIGSLCGGTGSGMFLDVAYSLRHLYGEQ-GA-----QIVSYLV 223

gi_193212596_ref_YP_00199 186 -----------LGLNVQ---TGALKIVVVASTAGGTGSGSFLDLGYLSTIIGNDV-ANQ---GVTTNLVL 237

gi_149916637_ref_ZP_01905 151 -----------HGMRHQ---SASIQVFVYFSVAGGTGSGGFLPFAYLMRDILDDR-S------ARLVGFA 199

gi_88602022_ref_YP_502200 134 ---------EKEGGGEK---RGHIAIWIIHSLGGGTGSGSFPALAIYLQKIVKEI-LGNKGITPHIYGVG 190

gi_153807774_ref_ZP_01960 144 -----------EKYELL---ANEIEVHIVFSLCGGTGCGTFINMAYLLRKFAPN---------CKLTGYG 190

[Consensus_aa:](http://prodata.swmed.edu/promals3d/info/consensus.html) .......................*h*p*lhll*st*h*t**GGTG**t**G**.*hl***D***h*t*h*.*h*p.*h*..............*h*..*hh*

[Consensus_ss:](http://prodata.swmed.edu/promals3d/info/consensus_ss.html) eeeeeeee hhhhhhhhhhhh eeeeee

Conservation: 7 855657 85

gi_160892492_ref_ZP_02073 217 LMPDVIFGNKSVTQDPE--LMFRMMANGCAALKEVDYY-MKLSEKD-----------DAYIFES------ 266

gi_163815161_ref_ZP_02206 217 LMPDVLFGYKAVYEDTE--LVSRLNANGCATMKEVDYY-MKLSEKD-----------DNYTFIS------ 266

gi_153954291_ref_YP_00139 229 FTPDVNLSRSADNQSA----QSYIIKNGFAALKELDYL-MGIGDRH-----------ERFTQKY------ 276

gi_226315300_ref_YP_00277 226 FTPDVNLSNKSLSSHT----RDYIMKNGYAALKELDYW-MNADERN-----------ERFRQQY------ 273

gi_219848227_ref_YP_00246 205 VMPSVFLQEI--ASYV---QRLRIQGNAYAALKELNYY-LSGQQQF-----------EATFPDYAYQTPD 257

gi_22298564_ref_NP_681811 211 PMPNAFAAISV---------GDRVLANGYAALMELSYF-SDYRTEY-----------LAQFSSS------ 253

gi_254410688_ref_ZP_05024 206 PMPNAFVGISV---------GDRVLANGYAALMELSYF-SDYRTEY-----------VAQFSNS------ 248

gi_284051667_ref_ZP_06381 213 PTPDAFLGIGG---------NIKTQENGYAALMELNYF-SDQNTNY-----------SVRYGLS------ 255

gi_37520551_ref_NP_923928 204 VLPGAFSGL-----------GDRVIANAYAALMELDYY-SRTDTRF-----------EAQYSTA------ 244

gi_15805565_ref_NP_294261 219 PHPDLGIAQKPD--------AEIWKTNAYHALAELNQY-HLHNDRE------------RYQSIKYPDKPE 267

gi_238059621_ref_ZP_04604 247 VLSGAFRRRL--EGPQ----RTRSEANGYALLRELDRL-MNTDRRH-----------PAEFRWS------ 292

gi_240169446_ref_ZP_04748 212 VTPDIFDSL---PESA----RTGVRANSLAMLGEIVAS-QSGAA--------------REHDVR------ 253

gi_227497172_ref_ZP_03927 219 YTAEVFNEV---PKDK----KDGMPGNTLAMLGEIIAA-QSSNGGK-----------AARLDEE------ 263

gi_269956178_ref_YP_00332 213 YAPDVFDHL---PDEA----RRGVRANALGTLAEVLNGYWNTDG-------------PSQATTA------ 256

gi_268325165_emb_CBH38753 175 ILPSSKESV-------------LPTGNALGTLKELHAL-MSHTEDL-----------IIE---------- 209

gi_83312057_ref_YP_422321 216 IFPEVYSGK-----------FGISQARAYSLFRELDRL-QEKHIPTYERYLRDGDQCSSEVQYD------ 267

gi_17229535_ref_NP_486083 196 VLPRVFANLPQ---------THLVKSNAYGALKEIEHF-WNLSPSN-----------PLEIDYG------ 238

gi_154496732_ref_ZP_02035 234 MLPSACGSE--PDPVR----KEKGNRNAYAALKEIDYF-MGLQSRGE--------VFRQQYG-------- 280

gi_189219502_ref_YP_00194 194 LLPEEHPKP----NWN----TGNYHANGYAALVELNAL-SIGKFS------------PCDIAG------- 235

gi_121606151_ref_YP_98348 192 LLPDAYPHP----NWD----TGNYHANGFAALTELNAM-SVGAYQ------------PYDVTG------- 233

gi_75908069_ref_YP_322365 224 ISPELYGNT------------PNMSANTYAALKELNYY-STPGTKF-----------AACYDIE------ 263

gi_193212596_ref_YP_00199 238 LLPSGYKGAG----------LTRTEANTYAALMELETC-MRQGSRY-----------IKQWAA------- 278

gi_149916637_ref_ZP_01905 200 ILPEAFESV---VGMN----RDGVYANGYAALKECEHL-MKLDTQHPI------DELTFHYDPR------ 249

gi_88602022_ref_YP_502200 191 ILPSGTNI----TDISTATFTKRYFANSFAALEEVKVL-AAASDVAP---------VTLKLPF------- 239

gi_153807774_ref_ZP_01960 191 VLPDVFEAM---SNAG----MAKVKPNAYGAIQDLDWL-MHLHMKS-----------DKIAFDY------ 235

[Consensus_aa:](http://prodata.swmed.edu/promals3d/info/consensus.html) *hh***P**p*hh*.................*h*.s**N**t*h*t*h***L**.**E***l*p*hh*......p...............*h*.........

[Consensus_ss:](http://prodata.swmed.edu/promals3d/info/consensus_ss.html) e hhh hhhhhhhhhhhhh h hh

Conservation: 56 5 5

gi_160892492_ref_ZP_02073 267 TTHKMVIRE------NLFDACMLVSGKKDSQGYLPEGTILMDTASFL-YKLAC------NKYIG-NND-- 320

gi_163815161_ref_ZP_02206 267 KDNKMVIRE------NLFDACMLVSGRKDSQGYIPEGFVLKDVASFI-YRLAS------NKYIG-NND-- 320

gi_153954291_ref_YP_00139 277 -RNRLTVD----SPMPPFNLCHLISATNI------DGRPMSNAYDYCMNVTAE------NIVNFMSSE-- 327

gi_226315300_ref_YP_00277 274 -GNVLTVQ----SPMPPFNLCHLISATNL------EGKALENAYDYCMNVTAE------NITNFMASE-- 324

gi_219848227_ref_YP_00246 258 GAKQVMYVR------RPFDTVFLVDRDNG-------SEGLSSIDEVK-QMIGQ------VIYLDTVTP-- 305

gi_22298564_ref_NP_681811 254 LADEVRYSC------PPFDFTYLVGTKNG--------ESEFKLDEIR-EMIAQ------NIFLDLTSD-- 300

gi_254410688_ref_ZP_05024 249 LTDEVRSKR------PPFDFTYLVGTKNG--------ESDFKLDQLR-EMIAQ------NIFLDLTSD-- 295

gi_284051667_ref_ZP_06381 256 ENTRIIENR------SPYDYLYLTGTSNQ--------EVSLKIETIQ-EMMAQ------QIFLDLVSD-- 302

gi_37520551_ref_NP_923928 245 ASDRISDQ---SGRDVPFNFCYLVGNSNN-------KVTFASLEAVL-EMVSQ------NIFLDFSSG-- 295

gi_15805565_ref_NP_294261 268 GTAILPGDA------MPYDLVYLLRPNST---------ETMDLLRLS-NAIAD------RMFLNVFVP-- 313

gi_238059621_ref_ZP_04604 293 ARNRHEMTA------APAQYTYFIDGRRSR--SSGRHLPAYDAESVCPVAVAD------AVYAHLLPT-- 346

gi_240169446_ref_ZP_04748 254 ILRALGQQHG-EGEPIPFARVFPVGRYVGA---DRTLFGDGSPFAVY-RGLAR------GLAGLMMSG-- 310

gi_227497172_ref_ZP_03927 264 LYSLMGGA---TKAGHAFKRVIPIGLKAGG---TGAVFGDGTTSGVF-RGMGR------GLARYIASP-- 318

gi_269956178_ref_YP_00332 257 LFETYGVTLSSTSRRSGPRYPFLVGAKNE-------HVTYKSQNDIY-RAMGR------SIASWVASE-- 310

gi_268325165_emb_CBH38753 210 ----DKNYS------NPFKLFFLLGRDLQ----------GQNRDEEL-ERAIT------RFLLDLGFMPG 252

gi_83312057_ref_YP_422321 268 DRGALRSI----LPGRLFDYQIFLGRQCH---------DEAERVAFF-SSVAN------ALDPYVDPN-- 315

gi_17229535_ref_NP_486083 239 -ITKVKADR------PPFDAVFLMDGVNK------NGTVVSRPNDLQ-NLIAD------GLYIQIGSQ-- 286

gi_154496732_ref_ZP_02035 281 -TFNVEISE------NIFDFCTLVEGVAD------GGVFFGDPADTARKVVAN------SILNMICTT-- 329

gi_189219502_ref_YP_00194 236 DGKKLEGLK------DPFNGCYLFTNFNE------NGLAVDVAEDIP-NILAD------FLYQKIV---- 282

gi_121606151_ref_YP_98348 234 VKQRLTLS-------DPFNGCYIFGNENE------NGLTVDVDKDLP-GIVAD------FLYQKIV---- 279

gi_75908069_ref_YP_322365 264 NLEFLQEKR------PPFDYTYLVSHQTG------GEYQILDQGKLC-NVIAH------KIALDFSGE-- 312

gi_193212596_ref_YP_00199 279 --GEVPRN----MPNSPYSDVYLIDTTNL------AGAKTGEVMDLY-DMVAD------TLFEDYSTS-- 327

gi_149916637_ref_ZP_01905 250 NKHKKLATR------RPYDLVYVVDRPQS-----------FSLEDVG-AALAD------ATYVQIFSP-- 293

gi_88602022_ref_YP_502200 240 HGEPIQVTE------RPFERYFLFGIDEE---------LTTKLRKEKGEMVDDYLSHANKIIVTMMFA-- 292

gi_153807774_ref_ZP_01960 236 INYIQETND------RPFNAFIFIDNKNP------NNDTYTHIDQIA-EMISL------ALITSAGE--- 283

[Consensus_aa:](http://prodata.swmed.edu/promals3d/info/consensus.html) ....*h*...........s*@*p.*hhhh*s................p..p*h*..p.*h*tp.......*lh*..*h*.s...

[Consensus_ss:](http://prodata.swmed.edu/promals3d/info/consensus_ss.html) h eeeee hhhhh hhhhh hhhhhhh

Conservation: 8

gi_160892492_ref_ZP_02073 321 --VNDD---------RKLLRDV-FFDNEKYSVTNWKMEHSDSEI---------------ATANCYYKVVS 363

gi_163815161_ref_ZP_02206 321 --VDND---------RKLLRDV-FFENEGYAITNWKMEHNDEDI---------------KKSNCYYKVVS 363

gi_153954291_ref_YP_00139 328 --VRESGG----VFAIQDYISN-LKQNT-------DNMAKP------------------YMSNYKYVIIG 365

gi_226315300_ref_YP_00277 325 --EKRSGEEFAIHDYISNIRTN-INQM-----------PKA------------------YAANYQYNVIG 362

gi_219848227_ref_YP_00246 306 --IGRE---------AASKREN-LNDLA-------SEQQN--------------------KFALAIAGVS 336

gi_22298564_ref_NP_681811 301 --FAPH---------KRSIRDN-IKAAW-------ASQDA-----GGR------------SYPKSFMAFG 334

gi_254410688_ref_ZP_05024 296 --FAPH---------KRSIRDN-IKGAW-------AQADP-----GGR------------GYPKNFMSFG 329

gi_284051667_ref_ZP_06381 303 --FSSY---------KRSIRDN-IKRDI-------GSSSDQAKN-SQQKAMGR-------SYSLNFFSFG 345

gi_37520551_ref_NP_923928 296 --FSQF---------KKLVRDN-IRKHW-------AGPDPL-------------------GHPQSFITFG 327

gi_15805565_ref_NP_294261 314 -------------------ETD-PMAYM-------VNAGPVT---VQQ------------GRAFAFSTFG 341

gi_238059621_ref_ZP_04604 347 --VGPA---------FSSYRVN-AKKYT-------------------------------RGSTDIYSTFG 373

gi_240169446_ref_ZP_04748 311 -----------------TASDQ-FVSYD-------LGNTAS-----PAGDRDLLGWGNSVWDPLPWGTYG 350

gi_227497172_ref_ZP_03927 319 ------------------AFES-YVQFD-------IANKVD------IPNHNAVSWGVDPT-ETAWGSFG 355

gi_269956178_ref_YP_00332 311 -----------------KLQDS-FTAYL-------EAQWPA-----TNNSIPDRLPLHAHGTETPFCAIG 350

gi_268325165_emb_CBH38753 253 GAVETK---------GKWLDLN-DLQNR------------------------------ARGYEDRFDTLG 282

gi_83312057_ref_YP_422321 316 ------------------IGPQ-MMEEL-------------------------------VNLNGLPFTVG 335

gi_17229535_ref_NP_486083 287 --IGLD---------AANVADN-IRAYL-------AVGEKVR------------------GRNINYCSFG 319

gi_154496732_ref_ZP_02035 330 --EAKAG-------KEPFMVDS-FLSNRTAISGVAVSRQSHRV--FPR------------DANYCYNVIG 375

gi_189219502_ref_YP_00194 283 -------------------TVR--NINW-------ESLGRSENAENGDNSPETLPGLNEPVRSKRFLTFG 324

gi_121606151_ref_YP_98348 280 -------------------AVN--NVNW-------ASLGRMENAENGDGSPETAPQGRMPERSKRFLAFG 321

gi_75908069_ref_YP_322365 313 --LAPV---------IKGHRDN-FLQHI-------IQWDK-----HPR------------PNGQRYLTFG 346

gi_193212596_ref_YP_00199 328 -VFANR---------KRSVSVNQNQYKIIPYEMLLPRETY-----GDM----------SITFSRAYSTFG 372

gi_149916637_ref_ZP_01905 294 --IIGD---------QQADYDNYTKENR-------RLFPDALWD-RKD------------GYSAFYGTLG 332

gi_88602022_ref_YP_502200 293 ----------------LPQYPK-GLENL------------------------------WKDVPSPFASFG 315

gi_153807774_ref_ZP_01960 284 --LSTT---------SASVSDN-LEKNI-------REGSM-----DIE------------NKKAWAAGLG 317

[Consensus_aa:](http://prodata.swmed.edu/promals3d/info/consensus.html) ..................*h*.ss.*h*....................................s.s..*@*.s*h***G**

[Consensus_ss:](http://prodata.swmed.edu/promals3d/info/consensus_ss.html) hhhh hhh h hhhhhh

Conservation: 5

gi_160892492_ref_ZP_02073 364 EADYKIPIREIEN-------------------ICES-----DLFNKAYKRL--------------FVSPF 395

gi_163815161_ref_ZP_02206 364 EADYHIPIKEIEN-------------------MCEN-----DIFSQAYKGL--------------YRSPF 395

gi_153954291_ref_YP_00139 366 ASSAVLPLEEITT-------------------YLAY-----KLFEKMQYMF--------------HNIP- 396

gi_226315300_ref_YP_00277 363 ASSAVLPIEEMTT-------------------YLAY-----RLFKKMEKMF--------------TVAP- 393

gi_219848227_ref_YP_00246 337 TASLVLPVRPVQV-------------------HVEMM--GMRILQEHILQE--------------QNSTV 371

gi_22298564_ref_NP_681811 335 LSSIEIPIAQIRA-------------------SLTY-----RLCQDFIHWW--------------LNESV 366

gi_254410688_ref_ZP_05024 330 LSTIEIPIAQIRT-------------------SLSN-----RLAKDLIGWW--------------LNESV 361

gi_284051667_ref_ZP_06381 346 IASIEIPVHAIRK-------------------ALAV-----KLAADLYQWW--------------LNANV 377

gi_37520551_ref_NP_923928 328 LSSIQFPIERVIN-------------------ACAS-----RLAGRVVRWW--------------MNPTP 359

gi_15805565_ref_NP_294261 342 LSTIEYPMRRILE-------------------ALKY-----RTLVHAVDRW--------------KDRKY 373

gi_238059621_ref_ZP_04604 374 VYLVEYAWEPVIR-------------------GLVD-----RAAADVLAALPTAVGDLGPEVDQFLSGAT 419

gi_240169446_ref_ZP_04748 351 FSSLRMGRDRYAE-------------------YAAQ-----RLARSCADKL--------------VSGHM 382

gi_227497172_ref_ZP_03927 356 YASLSTGRDRYAE-------------------YASQ-----RMARRSIDHL--------------LDGFR 387

gi_269956178_ref_YP_00332 351 SARVGLGRDRFVE-------------------YASQ-----HLARTVVMRI--------------VEEHE 382

gi_268325165_emb_CBH38753 283 YYECVFPTERLFL-------------------YYDIEDEIPRVRQKLVEIE------------------- 314

gi_83312057_ref_YP_422321 336 GARLTMPLTTYAE-------------------LFTW-----EMVDDVLVRL--------------FAPRQ 367

gi_17229535_ref_NP_486083 320 FATLALPVQQYER-------------------MKLE-----DTQNLLKNEL--------------MAATA 351

gi_154496732_ref_ZP_02035 376 YSSCVVPIDLMTV-------------------YVAK-----KVFDKVWEQF--------------ERCGE 407

gi_189219502_ref_YP_00194 325 VKRIAVPEPEIAE-------------------YLTF-----QFARQAALQL--------------RYNNW 356

gi_121606151_ref_YP_98348 322 IKRLAIPEEEISE-------------------YLTY-----SFARQAALQL--------------RFNHW 353

gi_75908069_ref_YP_322365 347 LAAIYFPRDTIVE-------------------IALI-----RVSLALVKFW--------------LNGKG 378

gi_193212596_ref_YP_00199 373 QAIIDTQLEQKKN-------------------VVLY-----RQVNGMLKAF--------------FGIAL 404

gi_149916637_ref_ZP_01905 333 SSVMILPRHDVLE-------------------YCAR-----RYAATAVRRY--------------VLLDD 364

gi_88602022_ref_YP_502200 316 ESELNIPIRLVKYLAGENDLLGPVIDENESVKAELR-----KLVIDAMKEF--------------L---- 362

gi_153807774_ref_ZP_01960 318 VCEILFRGRDLSE-------------------IYSI-----KSAKRLIELL--------------LNSCV 349

[Consensus_aa:](http://prodata.swmed.edu/promals3d/info/consensus.html) .tp*h*.*h*s.c.*h*.....................*hh*.......c*hh*.p*hh*..*h*...................

[Consensus_ss:](http://prodata.swmed.edu/promals3d/info/consensus_ss.html) hhhh hhhhhh hhhh hhhhhhhhhh h

Conservation:

gi_160892492_ref_ZP_02073 396 DNPQ-----------IETDIK-DALKEL-------------------DAFLHAEPGDEIN------LSVN 428

gi_163815161_ref_ZP_02206 396 KEGN-----------AEADIK-GVLTEL-------------------TEFLGEDAGENIK------LNVG 428

gi_153954291_ref_YP_00139 397 ---------------EENEVD-KFIRRLKLD-----------EDGVIERFEEQVQNRKP---------IT 430

gi_226315300_ref_YP_00277 394 ---------------TQEDAE-KFARKLGID-----------IDSISRKFEERV--PEP---------LP 425

gi_219848227_ref_YP_00246 372 K-------------DSLK---------------------------------------------------- 376

gi_22298564_ref_NP_681811 367 QLP----------PQLLEVTQ-SLLKPMNLL----------DMDLVLALA-------------------- 395

gi_254410688_ref_ZP_05024 362 QLP----------PQLLELVRGDILKRMRLT----------EAELIADLS-------------------- 391

gi_284051667_ref_ZP_06381 378 QLP----------SDVQQEAE-AELKELKLS----------SKELLNEIL-------------------- 406

gi_37520551_ref_NP_923928 360 SP-----------TAMRDLIR-TEILPGLVLAESDNQ------HQLLDSI-------------------- 391

gi_15805565_ref_NP_294261 374 EG------------RLEEDLD--TMGLTIP-------------NLTES---------------------- 394

gi_238059621_ref_ZP_04604 420 DARD------AEGAQPPPVLQ--ELDSDP--------------AREG---LLAP---------------S 449

gi_240169446_ref_ZP_04748 383 QPG--------NPASSNEQLE-SLLTSQWA-------------AICNELGLLA---------------AA 415

gi_227497172_ref_ZP_03927 388 SAG--------DFSGDAQRLA-ALWQARMP-------------EELAKMHLPQPTGQTVMAGGQAVDAAA 435

gi_269956178_ref_YP_00332 383 RSRRG----PSDDRTSKQVIR-DQATAIFP-------------AFLE-RSKLDERGL-----------ER 422

gi_268325165_emb_CBH38753 315 ----------------------ARLSD-----------------IRGKT-------------------ES 326

gi_83312057_ref_YP_422321 368 EGNQIVSLAWGSDTDRKTQAK-AKVAGLLP--------------LFGELLAVA---------------AG 407

gi_17229535_ref_NP_486083 352 NI------------DIASEIE-RFLQDCKLA----------EATTVLDSLTES---------------DR 383

gi_154496732_ref_ZP_02035 408 --------------PDSALAE-RFLLEANLS----------PKEVKAAMRLNPMRERFN---------AK 443

gi_189219502_ref_YP_00194 357 SETDGF-LADPRNLDMVSYVS-SPEQLERW-------------MLTDEHFILS---------------AP 396

gi_121606151_ref_YP_98348 354 QPASGF-IDEPRKLDFNEFVQ-QKETQLRW-------------LLSDDHLTLA---------------LG 393

gi_75908069_ref_YP_322365 379 QSP----------DPQ-KLLD-QFLIQSRWHNDLAKKD--GLTTKIAESV-------------------- 414

gi_193212596_ref_YP_00199 405 DDP--------KSNMPTDGER-DELLASRMHLSVENEIIDYDFSLKNEMY-------------------- 445

gi_149916637_ref_ZP_01905 365 P----------ALISPRQRAQ-FRRFAINPEEFAQLSD-DAKARRIDDAF-------------------- 402

gi_88602022_ref_YP_502200 363 -------------------------------------------RNLNESF-------------------- 369

gi_153807774_ref_ZP_01960 350 D--------------ADTIVN-QWIDSKDVNIRENNG-----FDNVIDYI-------------------- 379

[Consensus_aa:](http://prodata.swmed.edu/promals3d/info/consensus.html) p................p.*h*p...*h*......................c.*h*....................

[Consensus_ss:](http://prodata.swmed.edu/promals3d/info/consensus_ss.html) hhhhhh hhhhh hhhhhhh

Conservation:

gi_160892492_ref_ZP_02073 429 GLIQFGQF----TKPTYKMIKKHTD---GLREH--------------------MG--------------- 456

gi_163815161_ref_ZP_02206 429 GLIQYSQI----TKPTYKMIKKHTD---GLREG--------------------FA--------------- 456

gi_153954291_ref_YP_00139 431 GYK--DRE----KYNYSNVIKKQSV---NIDEE--------------------MK--------------- 456

gi_226315300_ref_YP_00277 426 GYE--NSE----RLNYSNVITQQVV---SIDHE-------------------LEQ--------------- 452

gi_219848227_ref_YP_00246 377 --------------NIQESINKWLQ---EELT-------------------DEN---------------- 394

gi_22298564_ref_NP_681811 396 -----AAG----DRPYMQEISRWVN---DLRN-------------------QISRENLLECTQQGLGGM- 433

gi_254410688_ref_ZP_05024 392 -----AAA----DRSYLAVISEWVN---SIRN-------------------EIATDNWLQCTQQGVKM-- 428

gi_284051667_ref_ZP_06381 407 ----LNQE----GKRYEVVIQQWIK---QLEN-------------------NINVEQRLKCTAQL-PNP- 444

gi_37520551_ref_NP_923928 392 ---AMGDN----AKPYTKEVADWAA---SLRK-------------------RRN---DLQIPFE------ 423

gi_15805565_ref_NP_294261 395 --LLLDAS----GASVRASLDAKKN---EVMR-------------------AVRNGNVQGA--------- 427

gi_238059621_ref_ZP_04604 450 STWLTAPG----GGPRLPDLPNLRL---PFEDIRPMRTDYQGQVVVDATQETLRVFWGGQNVPLTLKTQ- 511

gi_240169446_ref_ZP_04748 416 GSEDIN--------ALGNWVANVAF---PAQSVAPVVN--------TVIDRQLRSHLPSP---------- 456

gi_227497172_ref_ZP_03927 436 QAWLMNDS----ATANASLIWARAS---EMAQ-------------------QVMSTRPQA---------- 469

gi_269956178_ref_YP_00332 423 NDIIDE--------LKAKNLRDQSA---EFRA-------------------RVTQDIAGFINARSKGGK- 461

gi_268325165_emb_CBH38753 327 GR------------SELERFEGRIK---DIQR-------------------EINAYETKT---------- 352

gi_83312057_ref_YP_422321 408 PAEQVHGFA-VNRLNPREIVEKWYQ---FASA-------------------EVANRGLRQDELDYVIPLV 454

gi_17229535_ref_NP_486083 384 GGQVKPEFR-IGEMKYDRASLPTIK---ELYK-------------------RQLD--------------- 415

gi_154496732_ref_ZP_02035 444 ADEVFR-------VKGPYYMVNMMN---EIRR-------------------VLHADGKFASYAAAKSHG- 483

gi_189219502_ref_YP_00194 397 I-LESDGS----GTKWQPISRYWMN---ATTN-------------------FKQYTQEK----------- 428

gi_121606151_ref_YP_98348 394 I-LPEDAA----NKRWKSFTGEWEA---VIPN-------------------FKSLVRER----------- 425

gi_75908069_ref_YP_322365 415 -----EDT----NKNFSSNISTWRS---KLER-------------------SISECQNKDD--------- 444

gi_193212596_ref_YP_00199 446 -----RKG---IERTSYPIVNELLRV-NGISR-------------------------------------- 468

gi_149916637_ref_ZP_01905 403 ---TSKIRLLASQQDVDGGIWQRMA---ELPE-------------------LAR---------------- 431

gi_88602022_ref_YP_502200 370 ---LEDRA------------LAVFQ---EYRLL------------------------------------- 384

gi_153807774_ref_ZP_01960 380 ----LNKS----PRSPFSAIDNRANAQTEVDL-------------------FINSAMPKE---------- 412

[Consensus_aa:](http://prodata.swmed.edu/promals3d/info/consensus.html) ...................*h*.........*h*.p......................................

[Consensus_ss:](http://prodata.swmed.edu/promals3d/info/consensus_ss.html) hhhhhhhhhh hhhh hhhh

Conservation:

gi_160892492_ref_ZP_02073 457 -------------------------------------------EKLDN-MDKELPV-------------- 468

gi_163815161_ref_ZP_02206 457 -------------------------------------------TKMEN-FEKELPV-------------- 468

gi_153954291_ref_YP_00139 457 -------------------------------------------EYLRD-CINEYNK-------------- 468

gi_226315300_ref_YP_00277 453 -------------------------------------------GYLAK-AREAYIK-------------- 464

gi_219848227_ref_YP_00246 ----------------------------------------------------------------------

gi_22298564_ref_NP_681811 434 -----------MGTERG-----KILQFV---------------PWLTE-QVDNYRAAHLRELS--PDERL 469

gi_254410688_ref_ZP_05024 429 -----------LASEKG-----NIRQFV--------------DGYLKP-KVDNYRADHFREIS--PDERL 465

gi_284051667_ref_ZP_06381 445 -----------FGKETG-----KILEFV--------------SRYLNP-TVEEYRLDH--LRD---DQRR 478

gi_37520551_ref_NP_923928 424 --------------------------NL--------------QRFVLV-EQEKYAVHFSDGDT---DPRR 449

gi_15805565_ref_NP_294261 428 ---------------------RDAVKDL--------------RSAFDRDTGEGLRGLVH----------- 451

gi_238059621_ref_ZP_04604 512 ---------------------QQWHPVA--------------DHHARE-TDRQ----------------- 528

gi_240169446_ref_ZP_04748 457 -----------EGMTA-----AQWVPVF--------------RQAITN-RRDALAHACS----------- 484

gi_227497172_ref_ZP_03927 470 -----------AEMPL-----SDWIVGM--------------NQWLGS-VEPQIM--------------- 493

gi_269956178_ref_YP_00332 462 -----------KGVRL-----TELSMQV--------------QGSVND-KQQAVLSTLH----------- 489

gi_268325165_emb_CBH38753 353 ---------------------GLFHHVN--------------KAEISD-AKSKLDR-------------- 372

gi_83312057_ref_YP_422321 455 YRNPLISLERPEEETSPKDIAVKTYEDRRKAKEAKEDQEQSRNRFAGD-LRSAVELYL--DPK-----GG 516

gi_17229535_ref_NP_486083 416 -------------------------QME--------------QRTAKD-LAMNFYR-------------- 431

gi_154496732_ref_ZP_02035 484 -----------LLGRN-----EDW-ARV--------------EQLYGQ-LEQQVVVP------------- 508

gi_189219502_ref_YP_00194 429 --------------DPA----DKWLDRM--------------KGFLDARFLEQYRGQGV----------- 455

gi_121606151_ref_YP_98348 426 --------------ERA-----TWLDEL--------------TKLCEKRFQDDYRTLGV----------- 451

gi_75908069_ref_YP_322365 445 ------------------------RNGI--------------RQQLPREFREQFRKVQ--PGE---TENV 471

gi_193212596_ref_YP_00199 469 ------------------------FDDI--------------EKRIADRFEEIRVG------------GN 488

gi_149916637_ref_ZP_01905 432 -------------------------GKF--------------EAALDK-TQEELRDQTAGVREISADRIL 461

gi_88602022_ref_YP_502200 385 -----------------------------------------------------------GLAY------- 388

gi_153807774_ref_ZP_01960 413 -------------------------GEV--------------NGKIQS---------------------- 421

[Consensus_aa:](http://prodata.swmed.edu/promals3d/info/consensus.html) ...........................*h*.................*h*.p...p.*h*................

[Consensus_ss:](http://prodata.swmed.edu/promals3d/info/consensus_ss.html) hhhh hhhhhh hhhhhhh

Conservation: 6

gi_160892492_ref_ZP_02073 469 ---------------MIKSIKNK-------------------LWNSLDAV-----IARYIEKC--GP--- 494

gi_163815161_ref_ZP_02206 469 ---------------IIKSMKNK-------------------LWGALDDV-----IVKYMKEF--GP--- 494

gi_153954291_ref_YP_00139 469 ---------------VKTQYPGQ-------------------VEKEAREL-----VDGIFKDPKRGP--- 496

gi_226315300_ref_YP_00277 465 ---------------SKKQMPGD-------------------MISVFGEM-----ITRVFLRPQQGP--- 492

gi_219848227_ref_YP_00246 395 --------------------VEK-----------LYK----ELQQKLKDL-----YTEK------GL--- 415

gi_22298564_ref_NP_681811 470 H---------GDFFQRMYDNRNQ-----------ILQ----QARQSLEEE-----FYRIVEDRNRGP--- 507

gi_254410688_ref_ZP_05024 466 H---------GDFLKKMYDNRDQ-----------LIK----RGRKALEDE-----FYQILENRRQGP--- 503

gi_284051667_ref_ZP_06381 479 R---------GDFLQRMQDNGEK-----------LFQ----ETARTFKEK-----IYDYLEDKSQGA--- 516

gi_37520551_ref_NP_923928 450 W---------SDFYQKMFDNLER-----------LSV----QKCTELRAT-----VAKMIEDRFRGP--- 487

gi_15805565_ref_NP_294261 452 ----------RTVNDNRRVAAER-------------------VMENVRGL-----VSSRLLDYDQGP--- 484

gi_238059621_ref_ZP_04604 529 -------------------WGVA-------------------LRGAVARI-----MSRRD----GGP--- 548

gi_240169446_ref_ZP_04748 485 ----------DAGYRLAFGWQRA-------------------FADRLDDV-----VGNAIAD--FGL--- 515

gi_227497172_ref_ZP_03927 494 ----------PQLATMAVEYTRA-----------RAQ----EVHTAIVEQ-----VRTDLAD--LGL--- 528

gi_269956178_ref_YP_00332 490 ----------QATTEQAKHWVGT-------------------IQNHLQAR-----TAALVGQ--CGG--- 520

gi_268325165_emb_CBH38753 373 ------------------------------------------AKRKAAE------LKEEVFD--LDIRV- 391

gi_83312057_ref_YP_422321 517 E---------GSFEHGRRTMRKA-----------MTE----VMRRAVDET-----IAAEFGR--DGWVGT 555

gi_17229535_ref_NP_486083 432 ----------------------------------LEQ----TATTTIEAW-----LERSLNRP-NGL--- 454

gi_154496732_ref_ZP_02035 509 ------------MGSGLYD---------------VYT--------FVIKE-----LKRIIERN-AGLLT- 536

gi_189219502_ref_YP_00194 456 ----------KAFYETVGQSTRE-----------RAR----EIRQRLERE-----LFSEWI---NGT--- 489

gi_121606151_ref_YP_98348 452 ----------AGFYRTKLKARKD-----------MAR----EIRTRIEQE-----LINEWK---VGA--- 485

gi_75908069_ref_YP_322365 472 R---------GIWLTKLLQSSPN-----------ITK----ELKTNIDDY-----LIQLLTPSEPIFS-- 510

gi_193212596_ref_YP_00199 489 Y---------KGWAEKISEAITNINHDTFKDVETGSGLHVDAIQKRRREL-----LAELLDPNREHGL-- 542

gi_149916637_ref_ZP_01905 462 DGSWTPAASLNALQRQVAEAREN-----------IQR----HLQLQLDRVATGDWWTEFLAQ--AGPDES 514

gi_88602022_ref_YP_502200 389 --------FVGKLQNQINKLQIN-----------IQS----EYEEELDTW-----WETLRSE--SWS--- 425

gi_153807774_ref_ZP_01960 422 -------------------LSER-----------VSD----ELRKLIVKE-----INQE-----CGI--- 444

[Consensus_aa:](http://prodata.swmed.edu/promals3d/info/consensus.html) ..................p.......................*h*.p.*l*.p......*h*.p.*h*.p...**G**....

[Consensus_ss:](http://prodata.swmed.edu/promals3d/info/consensus_ss.html) hhhhhhhhhhhh hhh hhhhhhhhh hhhhhh

Conservation:

gi_160892492_ref_ZP_02073 495 ---------------------YAAIDIIGA-PTAGVA------DSTRGMMAEVK-----KLQ-ELHSKYT 530

gi_163815161_ref_ZP_02206 495 ---------------------FGAIDIIGA-PSAGVT------DSTKGMMAEVK-----KLE-KMMKDYT 530

gi_153954291_ref_YP_00139 497 ---------------------FYASRILFS-KGFCV---------IKTLEVEIT-----SLK-ERLSNM- 528

gi_226315300_ref_YP_00277 493 ---------------------FYASRLIHSDKGYCL---------MKMILSYIE-----SLK-ANLESF- 525

gi_219848227_ref_YP_00246 416 ---------------------DHVASVI------------------SEIQLEPD---------------- 430

gi_22298564_ref_NP_681811 508 ---------------------KFAQAFL------------------LQIRQIFL-----SQR-EKYDRE- 531

gi_254410688_ref_ZP_05024 504 ---------------------KFADAFL------------------ATVRQIFE-----DAS-DKFRRE- 527

gi_284051667_ref_ZP_06381 517 ---------------------RAIKALL------------------EQMRTSLQ-----TEI-EKLQRE- 540

gi_37520551_ref_NP_923928 488 ---------------------KFARQFL------------------EVLLEVLA-----DYK-SRFDQD- 511

gi_15805565_ref_NP_294261 485 ---------------------NVL-------------------------LEVMQ-----GVQ-PRVGEL- 501

gi_238059621_ref_ZP_04604 549 ---------------------RGGLAFL------------------DRVQQRLD-----LFR-SRVEQA- 572

gi_240169446_ref_ZP_04748 516 ---------------------PYARALV------------------DQLRRHID---------DVLTAP- 536

gi_227497172_ref_ZP_03927 529 ---------------------PYALQVI------------------GELRGEA------GVI-KRLAGS- 551

gi_269956178_ref_YP_00332 521 ---------------------PVADAVL------------------DLLKNELT-----FIR-DELRQE- 544

gi_268325165_emb_CBH38753 392 -------------------SDTEERKIAS-----------------ERNLERLG-----ALK-NKLLRE- 418

gi_83312057_ref_YP_422321 556 QP-------------KAPDQGTPLTRLY------------------GELRHLIGPEGPLNSIENMLAMV- 593

gi_17229535_ref_NP_486083 455 ---------------------ASTLKFV------------------SSLYQKID-----ELQ-QNVQRK- 478

gi_154496732_ref_ZP_02035 537 -------------------NTEEHQALFQRSFSWSPIDLTGGEKTSKVVMDYLDD---LLPQ-KEITRK- 582

gi_189219502_ref_YP_00194 490 ---------------------KSAHEIS------------------LLLGALIT-----HLK-GRYDRI- 513

gi_121606151_ref_YP_98348 486 ---------------------KSAWDVS------------------RLLIALTE-----TLD-ERLKAC- 509

gi_75908069_ref_YP_322365 511 --------------------IKSSRDWL------------------DALQHELH-----NYQ-FNLQEA- 535

gi_193212596_ref_YP_00199 543 --------------------IKALWMRVDNKERGGL----------DYTIELIG-----RIK-DRLENA- 575

gi_149916637_ref_ZP_01905 515 PELNLYEQRHVLIDFRESADGPLAPQAI------------------DELREQVQ-----RLQ-AE----- 555

gi_88602022_ref_YP_502200 ----------------------------------------------------------------------

gi_153807774_ref_ZP_01960 445 ---------------------GAAESII------------------LGIQAQIN-----VFM-KEMNSE- 468

[Consensus_aa:](http://prodata.swmed.edu/promals3d/info/consensus.html) .......................*h*..*hh*....................*h*...*h*.......*h*...p*h*.p..

[Consensus_ss:](http://prodata.swmed.edu/promals3d/info/consensus_ss.html) hhhhhhh hhhhhhhh hhh hhhhhh

Conservation:

gi_160892492_ref_ZP_02073 531 PSGEYSRIIESI-----------------------------CEIVAKRFFTFP----------------- 554

gi_163815161_ref_ZP_02206 531 PSGEYTRIIDSI-----------------------------KEIVAKRFFTFP----------------- 554

gi_153954291_ref_YP_00139 529 -----SREIKLK-----------------------------KELAEDKFLQAKK---------AILF--- 552

gi_226315300_ref_YP_00277 526 -----PPEIEGA-----------------------------RENANEKLGDARS---------AFI---- 548

gi_219848227_ref_YP_00246 431 ---------------------------DP------------DDFWLNRFKRKIF---------GKNH--- 449

gi_22298564_ref_NP_681811 532 -----IQNTWQPN--------ITNRQRQ-------------YENALQDINHFS---------TLFGI--- 563

gi_254410688_ref_ZP_05024 528 -----QDKIWSPK--------ESERQRQ-------------YEAALQDITEFK---------DKFGV--- 559

gi_284051667_ref_ZP_06381 541 -----AEKTWESL--------EKAAWAE-------------YNQGSSQIDEFS---------DRWMA--- 572

gi_37520551_ref_NP_923928 512 -----RQKNS------------IPQERS-------------AANALQTLTKQIDDQ----ARQFLLL--- 544

gi_15805565_ref_NP_294261 502 -----RGWEPSE--------GKSGAANG-------------VLDELDTIRTNTL--------LGMFF--- 534

gi_238059621_ref_ZP_04604 573 -----PRADP-------------GVAKAA------------RDQAEERVREDRGMF-----RGRHQLAYL 607

gi_240169446_ref_ZP_04748 537 -----MGQLGSM--------GSPDVVAL-------------PPNVDTALASLRGV-------ITN----- 568

gi_227497172_ref_ZP_03927 552 -----LAGMGMQ--------QPQHALVL-------------PQDFVAPLTARVK--------ETLTA--- 584

gi_269956178_ref_YP_00332 545 -----AQNFRVWS-QNE---ATKVTAKV-------------GSASGAEITAT------------------ 574

gi_268325165_emb_CBH38753 419 -----LTTPLNT--------RTYHPIAL-------------AEEEITSLKK------------------- 443

gi_83312057_ref_YP_422321 594 -----IESLQGQ---------EKFAAQS-------------ATDALAELERARA--------GGLLS--- 625

gi_17229535_ref_NP_486083 479 -----SKEAQSNFSTLKLEPQEEKIKEAAEAWF---PNKNTIQAACQRYKERADQK----WRIYLHW--- 533

gi_154496732_ref_ZP_02035 583 -----AKSFVD------------LLCSKKDEWTQLDPPEGKGQAAFDAAA--VIR-------EFIQD--- 623

gi_189219502_ref_YP_00194 514 -----EEEITK----------LKPREEEL------------AKKLKDQEKEWAK--------IGLIS--- 545

gi_121606151_ref_YP_98348 510 -----DDNVVR----------ARNAEEQA------------QSRVLGNLQKWSG--------MSLLS--- 541

gi_75908069_ref_YP_322365 536 -----ITDFGGMK-RAE---DIDKKWRD-------------AEQMIEDIEHKIGIPIINTKNSQVQA--- 580

gi_193212596_ref_YP_00199 576 -----NT-------GLV---RVLEENANWFQDLS-GFLQKDETRNLQDHLQQARG-------QLFGA--Q 620

gi_149916637_ref_ZP_01905 556 ------SDLG----GEG------RFRSE-------------MNAQAQEIKRTH---------GGWDKL-L 586

gi_88602022_ref_YP_502200 426 ---------------------------------------------CDQIERA------------------ 432

gi_153807774_ref_ZP_01960 469 -----LDDLSNR---------KPGLEAA-------------VKMAVKDLSDYDS----------KLF--- 498

[Consensus_aa:](http://prodata.swmed.edu/promals3d/info/consensus.html) ......p.*h*.................................p...pp*h*.p.............*h*.....

[Consensus_ss:](http://prodata.swmed.edu/promals3d/info/consensus_ss.html) hhh hhhh hhhhhhhhhhhh

Conservation:

gi_160892492_ref_ZP_02073 555 -SAKRETENGYYD-------ACIK-----ETLAAERNRIMDEID-------------------------- 585

gi_163815161_ref_ZP_02206 555 -SAKRETENGYYE-------ACIK-----EMLAAERTKLIDELD-------------------------- 585

gi_153954291_ref_YP_00139 553 -TKEGRKNDFIEA-------KSEE-----YILKAEEERIVRVIE-------------------------- 583

gi_226315300_ref_YP_00277 549 -SKEKKKNSYIEA-------KINE-----YQLLADQEKLE------------------------------ 575

gi_219848227_ref_YP_00246 450 -VSESPVDAVINH-------IRKNIAS-----IKNVSD-------------------------------- 474

gi_22298564_ref_NP_681811 564 -SKQAKMEEFCQQ-------ALEGIEG-SFNAIIQAKARLLAK--------------------------E 598

gi_254410688_ref_ZP_05024 560 -TKQAKMEEYCES-------ALIGLEG-CLTATIQRKARGIGL--------------------------E 594

gi_284051667_ref_ZP_06381 573 -TKENKMKEWCDQ-------AIGGIEK-SFQATLQRQSRLMAV--------------------------N 607

gi_37520551_ref_NP_923928 545 -DRKRRIDETFNA-------IMLALET-VYVSKIEVKSRTLGV--------------------------Q 579

gi_15805565_ref_NP_294261 535 -LKGKASQQLYPA-------LSRALDD-EIKSRVNQKVREVF----------------ADRGSGQKTEAG 579

gi_238059621_ref_ZP_04604 608 AAEQSLLTAHYQQDCQLRTLLMLERMT-QTVAVVRKEAQGW----------------RDAVD-------D 653

gi_240169446_ref_ZP_04748 569 --ADQVLATTLDG-------FRGAVRR-QVYATAAARLADV----------------FAVIGG------E 606

gi_227497172_ref_ZP_03927 585 TGEGQWAEQVREK-------AAVGIYQ-WLVYALAQNLAKV----------------LDDMVG------S 624

gi_269956178_ref_YP_00332 575 --SDPVLQDMIKS-------AVTSVIC-QYEAELRDLVMAL----------------IPDFVD------G 612

gi_268325165_emb_CBH38753 444 --------------------DREDLKN-LSFLAIMRKLDREDEYFRWTHSPINDGDIIFNPLV------N 486

gi_83312057_ref_YP_422321 626 --FGNWVEEPQRQ-------ARDRYSA-LIAWRQKRHLTEDMR--------------------------D 659

gi_17229535_ref_NP_486083 534 -KRCDKAAELCGM-------LRT----------------------------------------------- 548

gi_154496732_ref_ZP_02035 624 -EFKQVVDETMES-------FLVKLYSGNKDAKIPHLSPDEDQE-------------GHKPLMLA--AET 670

gi_189219502_ref_YP_00194 546 -DMFGKRKRLFDA-------ASVVLQD-LYSSRTAIEGWQFAK--------------------------K 580

gi_121606151_ref_YP_98348 542 KHLLGTPDSLLDA-------HGVHLQE-MYVYRTRAEGWGFAK--------------------------A 577

gi_75908069_ref_YP_322365 581 -EVKRVVQEVCKL-------IKHN-----FDFTVFQEALK------------------------------ 607

gi_193212596_ref_YP_00199 621 SQSEMKLKQISDA-------VRLY-----VRYHLYAAASREAA--------------------------R 652

gi_149916637_ref_ZP_01905 587 TRKDKDFEAARDR-------AVGL-----FNDYVDKIRAWLVKS------------ALLEFKGA---LGR 629

gi_88602022_ref_YP_502200 433 --GVVGLEEKHNL-------IVEL-----FNSRIEELVRKLDSL------------LVSPLKK------P 470

gi_153807774_ref_ZP_01960 499 -KNKARAEEYCED-------VINAATQ-LVINEREIIRRKAAI--------------------------T 533

[Consensus_aa:](http://prodata.swmed.edu/promals3d/info/consensus.html) .......pp*hh*p........*h*........*h*...*h*....................................

[Consensus_ss:](http://prodata.swmed.edu/promals3d/info/consensus_ss.html) hhhhhhhhhhh hhhhhh hhhhhhhhhhhhh h

Conservation:

gi_160892492_ref_ZP_02073 586 AQDLFGDTLRW----------LQQ----RAERLD--------------------------EIYS------ 609

gi_163815161_ref_ZP_02206 586 AQDLFGDTLRW----------LQQ----RAERLD--------------------------EIYS------ 609

gi_153954291_ref_YP_00139 584 FYEKVIENLSQ----------LNH----KIYKVY------------------TDILNELNKIFKEDGDIL 621

gi_226315300_ref_YP_00277 576 QMIEFYEELHR----------LLNAENNRIYSVF------------------TEILNALNQIFEQNGDIL 617

gi_219848227_ref_YP_00246 475 FVRKLRFYYED----------WHA-------MM-------------------QQQLSNVV---------- 498

gi_22298564_ref_NP_681811 599 VMTKLEEWLQG----------MEA----RLAKL-------------------NQRLLNLRDGFKAMA--- 632

gi_254410688_ref_ZP_05024 595 VISRLQEHLTV----------LER----RFNRW-------------------TQKLIQARDLYQEKA--- 628

gi_284051667_ref_ZP_06381 608 IQNRFLDVIQE----------VEN----EVDRW-------------------TGRISGSESKYRDSI--- 641

gi_37520551_ref_NP_923928 580 LVEALREETER----------LLS----DLTRF-------------------DRLMESLEAQFADKE--- 613

gi_15805565_ref_NP_294261 580 TLSIVEEEAEK----------LTR----RLTM-------------------LRRRLTNQADRWRDT---- 612

gi_238059621_ref_ZP_04604 654 ATRVVRLAMED------------------VRNERRQA-------------------ERFPL--------- 677

gi_240169446_ref_ZP_04748 607 LLVPLRDALSE-----------AL----ILLENAQAEPPS--------DVGLARLATDQYAAWPADAD-- 651

gi_227497172_ref_ZP_03927 625 ALKPLEYELNE----------KLK----VLTHDRQSLQD---------VAGVADVATDLYSAWPTEPTAA 671

gi_269956178_ref_YP_00332 613 VLEPLKDAIRD----------AAE----RLAVQRSAA-------------------GSVVALWPQA---- 645

gi_268325165_emb_CBH38753 487 YRHSIDNVMAS----------KYI----DILHDYGFLSLDAEGNVVNEEEKFGHLITVLSSRGDNFDDAR 542

gi_83312057_ref_YP_422321 660 LTREIRKRFEA----------WHH----TVEEAVRSAVLSTD----GSEPALKQVRDGNLARLKD----- 706

gi_17229535_ref_NP_486083 549 RIEKIQEQCQR----------IHS----NLDKV-------------------YRDVEQSYAEVSRQG--- 582

gi_154496732_ref_ZP_02035 671 LVDKLSTQASA----------LLQ----TRTGF------------------------------------- 689

gi_189219502_ref_YP_00194 581 FLPVIIEEIVL----------LKA----DVDKA-------------------ASMIAEALKTFEDQI--- 614

gi_121606151_ref_YP_98348 578 LLIEVIAEITD----------LKG----EVDRV-------------------ATTLQQALKKFEVGI--- 611

gi_75908069_ref_YP_322365 608 IVNELQKHVQE----------RGN----QVTAF-------------------SRVIENLQT-FYEKQ--- 640

gi_193212596_ref_YP_00199 653 LIHDLSEALGKQQGTDENGEPVWSGFIGELQEGRGLV---------------NAIIESAEEQIALT---- 703

gi_149916637_ref_ZP_01905 630 AAEHLRRTFRD----------IES----SAGRL-------------------ARSLEELARRYEFDGGVE 666

gi_88602022_ref_YP_502200 471 DLRERKEKIEK----------ILH----DLEVLKV-----------------KTLKVRTLKQY------- 502

gi_153807774_ref_ZP_01960 534 FYTGLQAELLE----------YYT----RISNI-------------------KDMLVAIFSNYTNRL--- 567

[Consensus_aa:](http://prodata.swmed.edu/promals3d/info/consensus.html) *hh*p.*l*...*h*p...........*h*......p*l*.......................p.*h*......*h*.......

[Consensus_ss:](http://prodata.swmed.edu/promals3d/info/consensus_ss.html) hhhhhhhhhhh hhh hhhhh hhhhhhhhhhhhh

Conservation:

gi_160892492_ref_ZP_02073 ----------------------------------------------------------------------

gi_163815161_ref_ZP_02206 ----------------------------------------------------------------------

gi_153954291_ref_YP_00139 622 AKGE-------EIEGALGRTYSW------------TVVRV--PDLTDYIERVVNKETA--------DELM 662

gi_226315300_ref_YP_00277 618 INGG------EESDRTGNKTYYW------------NIVSV--PDISKVVSNILEQKDA--------DDMI 659

gi_219848227_ref_YP_00246 499 -----------PSR-----PQSMSEP--KSPQNNLSLFEL------------VTELGYER---------- 528

gi_22298564_ref_NP_681811 633 -----------DSQADSADALR---------INGVKLYDR--QELNGLYQDLVERYAGA--NTGVESTFT 678

gi_254410688_ref_ZP_05024 629 -----------NRQAESADALV---------INGIKLYDR--QELNGLYQDLIEQLAGA--SEGSQTAYE 674

gi_284051667_ref_ZP_06381 642 -----------AQSENYIEQLE---------LVGIKLFER--RELKELYDDFISKSQGLDILFSQLTQDI 689

gi_37520551_ref_NP_923928 614 -----------NTYLRETRTLT---------VNGILLYDE--KDVGMIYQNTLGEREQA--VCGLISEKV 659

gi_15805565_ref_NP_294261 613 ----------RARLEN--DSAS---------VNGLSLFEP--SPNGSVDK-----EQEL-----AVSDKQ 649

gi_238059621_ref_ZP_04604 678 -----------RRVVP-------------------AVDDH--RTEDWLYDRSVGAADGD--GLRPRLSDV 713

gi_240169446_ref_ZP_04748 652 -------EFVPSRFAEA--------------NNEVLLISS--SAFKQRYESDLVKVIAAGHTLVPFRAAV 698

gi_227497172_ref_ZP_03927 672 MTSE---QVVPQRFSTA--------------HNEVVLMDV--ATYPSAFEEHVVEAVPP-AARGGFDQAY 721

gi_269956178_ref_YP_00332 646 -------VAIPARLLPA--------------PNEFLLEEP--SSYPTILAELIDRT-----VTASGSDGR 687

gi_268325165_emb_CBH38753 543 LGGGAFKSMVTERFAKD--------------ADVLKLDAPARAHSFGIYTLMI----------------- 581

gi_83312057_ref_YP_422321 707 ---------RLLRMTRDAS-------------TMISLQPN--DTTMQGFMTVLRNVAVM-DQDRDLATAA 751

gi_17229535_ref_NP_486083 583 -----------NSDNPFIHTIQ---------RVDLESKRP--KVSGEDFIRWYREKSQS--LTNWSEKKA 628

gi_154496732_ref_ZP_02035 ----------------------------------------------------------------------

gi_189219502_ref_YP_00194 615 -----------AARCADQGGIIDL------RKQVIRFYDP--ELVKSVTKRLIKDQK----EATTQTQRV 661

gi_121606151_ref_YP_98348 612 -----------QARLNDAGSGDL-------RQHLIRFYDP--VQVKTISRRLVLDEA----EQKTQSGRV 657

gi_75908069_ref_YP_322365 641 -----------DSDLRQLNFDE---------MSGEAIFDS--EDIDRCYQTMLPEDDLR--RQLVLASSE 686

gi_193212596_ref_YP_00199 704 -----------NEAMKQNHAMYFVLPAPKSQIDELELLPP--SQARQWAEEAFE---------------- 744

gi_149916637_ref_ZP_01905 667 GLRSEANEYALDVELLQHPSAR------------MRFWSW--YYVDQIESRPELQ----------NQDAV 712

gi_88602022_ref_YP_502200 503 -------------------------------------------VDTKIGEKLSLQ----------NRDSK 519

gi_153807774_ref_ZP_01960 568 -----------AEIQNRVGK-----------ASQTFQID-----LAQSFVNKVVLEEEE----IQVEEFL 606

[Consensus_aa:](http://prodata.swmed.edu/promals3d/info/consensus.html) ............p.......................*h*bp.........*h*.p.*h*.p...........pp..

[Consensus_ss:](http://prodata.swmed.edu/promals3d/info/consensus_ss.html) hhhh eee hhhhhhhhhhh hhhh

Conservation:

gi_160892492_ref_ZP_02073 ----------------------------------------------------------------------

gi_163815161_ref_ZP_02206 ----------------------------------------------------------------------

gi_153954291_ref_YP_00139 663 RKF------------------SYR-LLEESQKWL--DEV------------------------------- 680

gi_226315300_ref_YP_00277 660 RDF------------------TSE-LLRHSDQWV--KEQ------------------------------- 677

gi_219848227_ref_YP_00246 529 ------------------------------------EVPAP---------------GNLPNAQ------W 541

gi_22298564_ref_NP_681811 679 IGL--NQLCTTT--------ATTV-LQEASSLWK--ETRAANE--------------------------- 708

gi_254410688_ref_ZP_05024 675 MGM--NGVCSTL--------SEEV-LNLASPLWK--ETRMADE--------------------------- 704

gi_284051667_ref_ZP_06381 690 KV-----------------------ASQNSKFWT--QSAYGQQ--------------------------- 707

gi_37520551_ref_NP_923928 660 LT------------------ASDA-MLFDLHTFD--TFRARDL--------------------------- 681

gi_15805565_ref_NP_294261 650 REA-HAAR-----------------LIRSWDALV--KGVLPG----------------VNDP-------- 675

gi_238059621_ref_ZP_04604 714 GSALAWRVSLAN---------------GAS-------------------------TLVLASASGQSASAG 743

gi_240169446_ref_ZP_04748 699 GEA-TTRVILGEWQTTGGMVAPGG-LLERSANWV--TRALGSDPDTGRSRVPSVAQFDVHTR-------- 756

gi_227497172_ref_ZP_03927 722 REA-VREIIRGEWEQGSGAPAPAD-LLEIASPWV--PAGLPGVMS---QMPAPA-QYEVRLS-------- 775

gi_269956178_ref_YP_00332 688 REA-ELQVLLGTDD----PTGDTQALVNAHQTWV--PKNHNLAPDF-GLIPRRA-HFAVASS-------- 740

gi_268325165_emb_CBH38753 582 ----------------------------GLQPWA--PGPGL----------PPR---------------- 595

gi_83312057_ref_YP_422321 752 LEAAAWKAEIGE--------------DGRP-------------------------RLALNVGEAG-FDGT 781

gi_17229535_ref_NP_486083 629 ED------------------VKDEILAFMDETYR--PLTSM----------------------------- 649

gi_154496732_ref_ZP_02035 ----------------------------------------------------------------------

gi_189219502_ref_YP_00194 662 RT------------------ALAE-KLGERLDFK--TLN-------------------ERVS-------- 683

gi_121606151_ref_YP_98348 658 RA------------------ALIE-KIGPDAGFA--QFN-------------------QRVP-------- 679

gi_75908069_ref_YP_322365 687 IT------------------EPAG-RGQSLASFIDRERTTPEQ--------------------------- 710

gi_193212596_ref_YP_00199 745 ------------------------------------DFGG------------------------------ 748

gi_149916637_ref_ZP_01905 713 LV------------------ALRE-ALRP--RFD--ERGGS----------VRP---------------D 734

gi_88602022_ref_YP_502200 520 EK------------------ILGV-ASIV--TFA------------------------------------ 532

gi_153807774_ref_ZP_01960 607 KSL------------------IY---PEKIYEFS--DKRTTEI--------------------------- 626

[Consensus_aa:](http://prodata.swmed.edu/promals3d/info/consensus.html) ......................................................................

[Consensus_ss:](http://prodata.swmed.edu/promals3d/info/consensus_ss.html) hh hh hhhhh hhh h

Conservation: 5

gi_160892492_ref_ZP_02073 610 ---------------------QFGED-LKNSIEDL----------------------------------- 622

gi_163815161_ref_ZP_02206 610 ---------------------QFGED-LKNSIDDL----------------------------------- 622

gi_153954291_ref_YP_00139 681 ---HIDVVGSISNF----VSEEYGNV-ITQSMEEF----------------------------------- 707

gi_226315300_ref_YP_00277 678 ---ELDIVSSISEF----LSEKFGEV-ITKSMEEF----------------------------------- 704

gi_219848227_ref_YP_00246 542 PIDRFVFS--LASL----SGDNRAKL-INEVGLHAVFNLVYKFEKGEHKLFLDHPPNHDPVKAYLETVQG 604

gi_22298564_ref_NP_681811 709 VMRLLDVAQ-LADV----QESDLAEI-IQETVRRT----------------VQNAPE------------- 743

gi_254410688_ref_ZP_05024 705 VMRLFDITE-IPDV----QDEDLRQI-IVDRSQVV----------------VAKAPQ------------- 739

gi_284051667_ref_ZP_06381 708 EFHLLDVAR-IDEL----QYPQFEDV-VEQFTRRT----------------IQEAPA------------- 742

gi_37520551_ref_NP_923928 682 FSRLLDA--------------SLDEF-VGRSRARV----------------------------------- 701

gi_15805565_ref_NP_294261 676 -DWLLGA---WAVGQDNFERAQLNAL-EQAAVEPF----------------ESSLRS------------- 711

gi_238059621_ref_ZP_04604 744 PAETVVRALRD------RARPLIAPL-RQLSVFEL----------------LERSRV------------- 777

gi_240169446_ref_ZP_04748 757 PAELLARARLYVE----RPGEAFDEF-CRVSLRDY----------------VQGAGA------------- 792

gi_227497172_ref_ZP_03927 776 RAQVLGRARAFVG----RRGEAFESF-VSQSLRDY----------------LTDPAA------------- 811

gi_269956178_ref_YP_00332 741 PDQILARAEAWLR----KEGTAVGRY-MTQGLRDY----------------LTPENV------------- 776

gi_268325165_emb_CBH38753 596 -----LRELEWIER-------------------------------------------------------- 604

gi_83312057_ref_YP_422321 782 ALARLHDALYA------RFRPAIDQRLQPFDIFDY----------------LLYVKD------------- 816

gi_17229535_ref_NP_486083 650 --------------------NIEQVL-ADSNPED------------------------------------ 662

gi_154496732_ref_ZP_02035 ----------------------------------------------------------------------

gi_189219502_ref_YP_00194 684 KTVFLDTLEKQCME---NAKIAHNNL-VQTAAERL----------------------------------- 714

gi_121606151_ref_YP_98348 680 ESAFLDVLETVCED---NARIAHQNL-VQNPKERL----------------------------------- 710

gi_75908069_ref_YP_322365 711 LQTEIDLK--VDSL----FASRVTNI-VNSVIKRF----------------MQK---------------- 741

gi_193212596_ref_YP_00199 749 TQELFKML--KDE-------DGRSEL-LGKLRNRA---------------LDLIGDD------------- 780

gi_149916637_ref_ZP_01905 735 ARRVVQE--------------IVDRL--VEMAKER----------------LGPEILGDPK----ADDAA 768

gi_88602022_ref_YP_502200 533 ---RKKD--------------AYRQ----NVLKKR----------------LGLSGSGRVLNPALSEDIM 565

gi_153807774_ref_ZP_01960 627 EKIFLDYAHTLS----------TARVWAKTTVDDI----------------------------------- 651

[Consensus_aa:](http://prodata.swmed.edu/promals3d/info/consensus.html) ....*h*.................*h*.p*h*.*h*.ps.pc*h*...................................

[Consensus_ss:](http://prodata.swmed.edu/promals3d/info/consensus_ss.html) hhhhhhhhh hh hhhhhh hhhhhhhh

Conservation:

gi_160892492_ref_ZP_02073 623 ----------------ANEG--------KVT-----------------------TKN-------ILKNAA 638

gi_163815161_ref_ZP_02206 623 ----------------SIAG--------SQT-----------------------VVN-------ILKKAK 638

gi_153954291_ref_YP_00139 708 ----------------LSLQ-----YGEDKS-----------------------IIS-------IIKDNI 726

gi_226315300_ref_YP_00277 705 ----------------LVIK-----YGQDET-----------------------LDR-------IVERKI 723

gi_219848227_ref_YP_00246 605 FVNPMLSESW-TAIRYLRWF-----YNNIQYYSNTS------------TESRGSPID-------PLRHLK 649

gi_22298564_ref_NP_681811 744 --ESCLVRDL-TACDRLLQV-----YRNDEG----------------------EILS-------VLRLAY 776

gi_254410688_ref_ZP_05024 740 --SSKLQQDL-AACDRLFKV------FNDDA----------------------EIVN-------NLRIAY 771

gi_284051667_ref_ZP_06381 743 --NSKLYTEM-DACTRFMRL-----YPKTQE-----------------------QER-------EIERLF 774

gi_37520551_ref_NP_923928 702 -----------SAARKFLET-----YPTVEQ-----------------------QEA-------QIKTTF 725

gi_15805565_ref_NP_294261 712 ------SGKD-VIRRLYEQR-----SPSFD------------------------PDS-------QAMHAA 738

gi_238059621_ref_ZP_04604 778 ------PADE--LGRELLDGSDWLVNYDDEE-----------------------HRR-------LVNLAQ 809

gi_240169446_ref_ZP_04748 793 ------PESE--LTARRHD-----------------------------------IAT-------KFAEAL 812

gi_227497172_ref_ZP_03927 812 ------GEFE--QARRADE-----------------------------------VLE-------GMQRAM 831

gi_269956178_ref_YP_00332 777 ------PAAE--HARRLAR-----------------------------------FEA-------KFIAAL 796

gi_268325165_emb_CBH38753 605 --------------------------AYNES-----------------------DFT-------KLQRHH 618

gi_83312057_ref_YP_422321 817 RKG-----------------------------------------------------I-------TTDAVV 826

gi_17229535_ref_NP_486083 663 ------------AGE-------------------------------------------------DLQQLG 671

gi_154496732_ref_ZP_02035 ----------------------------------------------------------------------

gi_189219502_ref_YP_00194 715 -------IGV-SIIEKLKDR-----YSDRQS-----------------------LRL-------YLDGVV 741

gi_121606151_ref_YP_98348 711 -------LGV-SIIDKLRDR-----YGADPQ----------------------ELKS-------YVNELV 738

gi_75908069_ref_YP_322365 742 -------------------------YPLAA------------------------RST-------RLAQVM 755

gi_193212596_ref_YP_00199 781 EMQDEE-------------------NPLFAALDQ-----------------HQNLSM-------LFSDFF 807

gi_149916637_ref_ZP_01905 769 RRLGLRIDDA----LALEAA-----YYGRQTDDPRGEPVDLLEDRPLDPTALWRDEKVRTYARRKIDLAI 829

gi_88602022_ref_YP_502200 566 NSVSLVRDINVAHMKSLADY-----FQTLKFSQKDVDKI-------IKNRIEQSRDR-------HLSVAI 616

gi_153807774_ref_ZP_01960 652 -------------MNKMDEE---------------------------------SFTH-------ILKMAI 668

[Consensus_aa:](http://prodata.swmed.edu/promals3d/info/consensus.html) ................*h*................................................*h*p.*hh*

[Consensus_ss:](http://prodata.swmed.edu/promals3d/info/consensus_ss.html) hhhhhhh hhh hhhhhh

Conservation: 5

gi_160892492_ref_ZP_02073 639 R-----HEFLPT-----------------------------------D-------YVNDSR--------- 652

gi_163815161_ref_ZP_02206 639 R-----HEFLPT-----------------------------------D-------YVTSKR--------- 652

gi_153954291_ref_YP_00139 727 APKLDKDAVPIF-------------HINN-MEA---VNFPT------WS------MVSVPTN-------- 759

gi_226315300_ref_YP_00277 724 ASKLDEEAIPVF-------------HLSNNLGN---MHFPS------WG------FVSVPLK-------- 757

gi_219848227_ref_YP_00246 650 L----RSEKPFL-------------RIDNARL----------------------------GTEHA-SDIE 673

gi_22298564_ref_NP_681811 777 Q-----KSKPLL-------------LLSQAVMSGRDAGFT----PSVNT------NIAIVGGENT-TDFA 817

gi_254410688_ref_ZP_05024 772 N-----KSKPLI-------------LLARSVLTGKDAGFT----PATNT------NVALLGGRNT-SDPA 812

gi_284051667_ref_ZP_06381 775 N-----LSKPLI-------------RLDTAIPQE--GGFN----YIQFH------LAGIVGGENT-PETA 813

gi_37520551_ref_NP_923928 726 E-----KSEPFL-------------RFSQEQARLGWDDKP----EKRQT------LVGIQGGNKP-DDPA 766

gi_15805565_ref_NP_294261 739 T-----QAELFL-------------QLNEPLGQVDPMSPL----PRRKL------LVGMHMT-----PDF 775

gi_238059621_ref_ZP_04604 810 D-----DDG------------------EPQQQVYA--------------------FAALPSS----GAP- 831

gi_240169446_ref_ZP_04748 813 S-----LARPLA-------------SVSDQALTRVHPGQQ----VEYRY-----KFSEIPFA----GQPV 851

gi_227497172_ref_ZP_03927 832 A-----MARPLV-------------QVDAQVFQRL-HGSA----PALSF-----NFSSVPFR----HTKV 869

gi_269956178_ref_YP_00332 797 N-----ASTPLV-------------KVNPAVLVNVHGTDK----VPYTS-----HFGEIPLP----DNSP 835

gi_268325165_emb_CBH38753 619 SLF-YGTPKPFS-------------EIT-----------------------------GIPYSPGA-DEKN 644

gi_83312057_ref_YP_422321 827 E-----QLGNAAK-----------VLLDVS------CAGI-------CR------WVYARPS-----GEH 856

gi_17229535_ref_NP_486083 672 K-----LAVPLW-------------QYQDSEIP-----------AKQQHVITEFYYYGVESNNTIFSNPP 712

gi_154496732_ref_ZP_02035 690 ---------------------------SLNDCG-------------CSK------YMTVPDGCR----WL 709

gi_189219502_ref_YP_00194 742 K-----AAGNYL-------------LFNADEKRRSGPGINPAAQIGVSN------FTVILPK-----AAE 782

gi_121606151_ref_YP_98348 739 S-----RAGNFV-------------ALEPLEIHRAAPGIPLGVPTAVGK------FTVILPK-----APE 779

gi_75908069_ref_YP_322365 756 Q-----EAEPLL-------------RLNLSDPYFREDP------AKSSK------LIGFKDK----DELE 791

gi_193212596_ref_YP_00199 808 Q-----RAMPWI-------------AAKV-DGYLKPQNPN----DQYKC------FIGVKDS----SKFR 844

gi_149916637_ref_ZP_01905 830 N-----KAQPLS-------------RYQPEL-RGTIK-------HPNML------LVGLHDS------LR 861

gi_88602022_ref_YP_502200 617 G-----SGGEILTNPREELFILCNMLHESAL-GDNIA-------FASMK------VVKIPSQ-------- 659

gi_153807774_ref_ZP_01960 669 N-----KSAPLF-------------RYNYR-------GYTPQEKPCDSY------YIGVPDKKNS-RLVE 706

[Consensus_aa:](http://prodata.swmed.edu/promals3d/info/consensus.html) .......s.s*hh*..............*h*p...........................*hh*s*h*...........

[Consensus_ss:](http://prodata.swmed.edu/promals3d/info/consensus_ss.html) h e eee

Conservation: 5

gi_160892492_ref_ZP_02073 653 INEVKDG--LIRLMLDNE--------------AN-----ID-------------NGRVVPVKD------- 681

gi_163815161_ref_ZP_02206 653 IGEMSSG--IIKLMLDNE--------------AN-----ID-------------NGRVIPVKD------- 681

gi_153954291_ref_YP_00139 760 AKKILEG---IKEYKRNS---------HRGDSIN-----IKE---STV------TNRIFWLNT--KNGVP 801

gi_226315300_ref_YP_00277 758 APSILKG---IKNYQNTA---------ISGSRFT-----VKE---SEV------KNRIFWLNT--KNGVP 799

gi_219848227_ref_YP_00246 674 VQRLIGYSPLLTSAERHDPDSVLSDFDSYYHVDT------------GT------PNRIDVLVI--SFGYR 723

gi_22298564_ref_NP_681811 818 AKKLIPL---LKNLVKPN--------GQSITSDD-----IKPL-GDRE------RHRIVFVQE--MGGFS 862

gi_254410688_ref_ZP_05024 813 AQKIIPK---LEEFL--------------GNEES-----IKPL-GDLE------RHRLVFVQE--TGGFS 851

gi_284051667_ref_ZP_06381 814 AQQQVPL---LKKFF--------------VQTDA-----IAPL-SKRE------RHKILGTHE--IAGFS 852

gi_37520551_ref_NP_923928 767 AATLLPF---IRKTS-------------TITDKD-----IRPL---GE------SHRIFFVQE--AGAFP 804

gi_15805565_ref_NP_294261 776 RRAIQPW---VNKSP-------------EAKET-----------EGID------PYRVVMLEE--WYRFA 810

gi_238059621_ref_ZP_04604 832 GAALSRE---LWAFL-------------GKQRVD----LNDIV-PSNEAEERPVTDKIVLFAS--RHSLR 878

gi_240169446_ref_ZP_04748 852 GMALADT---LRSNP-------------RVDQASK-DNFARALTDDDG------VTHIDIFGS--YPNYS 896

gi_227497172_ref_ZP_03927 870 ATELVTY---ASHET-------------SFDGPAVAQTIEQAL-SDDQ------VSRVDVFGS--YPRTL 914

gi_269956178_ref_YP_00332 836 AKATVRR---VLESR-------------DEWREE----VNKAF-DDGN------GGFIDVFTH-LNAAFE 877

gi_268325165_emb_CBH38753 645 RDTVT-----------------------------------EYW------------KNYEIIESGALWNNV 667

gi_83312057_ref_YP_422321 857 KARLVDE---LNARL---------------RPVAG-DELRDAKQNHSD------RTSLTLLRA--AEPD- 898

gi_17229535_ref_NP_486083 713 LSNRLPK---GKGNVS-----------------------FVP---TGE------PHKLTLFRI--EIGVP 745

gi_154496732_ref_ZP_02035 710 DKHIANY---AVGHG-------------VVDGSD----SVYR---SSA------RDEIVLYRL--YVCVP 748

gi_189219502_ref_YP_00194 783 MEDYLKL---LREVFFEC---------IRNPSKE-----FIE--NDNR------PNEIILVSI--TNLFP 825

gi_121606151_ref_YP_98348 780 QAEFSRV---LKDALREA----------KTGDVE-----IIE--SDGR------LNEITLVSI--TNLLP 821

gi_75908069_ref_YP_322365 792 VRQFKTV---LAQDL-------------GIESSV-----IKA---TQS------EDEILIVNE--YAGFP 829

gi_193212596_ref_YP_00199 845 EKYGEQL---LSKLP-------------TVTMMTRKEVGFVE---IDA------PGKLICYTE--LSGLP 887

gi_149916637_ref_ZP_01905 862 TGPFHEV---YGEAT-------------GDE--------SQEL-EWPD------SHRIVFYRS--ILGVP 898

gi_88602022_ref_YP_502200 660 ---------------------------------------------TYN------DEKVEFIDY--TLNLS 676

gi_153807774_ref_ZP_01960 707 DNKLKNL---LPP---------------GSVNLD-----FAS---IGV------KDRVIIYRQ--VGVVP 742

[Consensus_aa:](http://prodata.swmed.edu/promals3d/info/consensus.html) ...*h*......*l*......................s.....................pc*l*.*hh*p.....s*h*s

[Consensus_ss:](http://prodata.swmed.edu/promals3d/info/consensus_ss.html) hhhhhhh hhh eeeeee

Conservation: 5 8 5

gi_160892492_ref_ZP_02073 682 ---------AME-------KIYRRLFSGIGAYG-----------PEK----------------------- 701

gi_163815161_ref_ZP_02206 682 ---------EME-------KIYREFFSGVGAYG-----------PEK----------------------- 701

gi_153954291_ref_YP_00139 802 LYSYAPI-KHYE-------EIYEKTLFERDGIG-----------RHLYQSENRN----WIYLP------- 841

gi_226315300_ref_YP_00277 800 LFAYTPL-KVYE-------ESYERTILDREGIG-----------RHLVQTEKNN----WAYLP------- 839

gi_219848227_ref_YP_00246 724 IADLRDL-PELK-------KAYEYFMKTQ-------------KETLHIHKNWSD-----GRLP------- 760

gi_22298564_ref_NP_681811 863 LRCIEGM-SELR-------QAYQDWRGQMITAKRDRLRGENRDLPIPVHLQKD------PPFW------- 911

gi_254410688_ref_ZP_05024 852 LRCIDGM-KELR-------QSYQDWKGESIVAKRAQLRGESRDLPIPVHLQKE------PPFW------- 900

gi_284051667_ref_ZP_06381 853 LRCMAGT-ENLR-------KAYQKWRGERIQAERDLLKGMNAKLPAPVHIQKD------MVFW------- 901

gi_37520551_ref_NP_923928 805 LRLIEGM-ARMR-------SVYRAVKAV---------------ERNPLHTDSDE-----DRFG------- 839

gi_15805565_ref_NP_294261 811 LRGADDV-RELSYSKPTRFNTYFTRKRSDI---------------------------------------- 839

gi_238059621_ref_ZP_04604 879 LAAFDGF-TDLR-------RSYEEQRASP-------------------------------PSP------- 902

gi_240169446_ref_ZP_04748 897 PLVFDSVLRPPA-------QQWAEVAGPG--------------RMQFWRYRRS------RPLQ------- 932

gi_227497172_ref_ZP_03927 915 PVAYSGLLKSVS-------TAWHHATGSS-----------GARA-SFWQFRRA------RPLP------- 952

gi_269956178_ref_YP_00332 878 PVVFDSLMKPIA-------SDWGQKSMGA-----------DGRA-EFWRWRRA------RPLT------- 915

gi_268325165_emb_CBH38753 668 PIVLAQSLKMFD---------------------------------------------------------- 679

gi_83312057_ref_YP_422321 899 PDKIPNL-LDCQ-------RSYVQSLSKNL---------------------------------------- 920

gi_17229535_ref_NP_486083 746 LFTLNGM-KDME-------MAYLDPDKV------------------FKHLHRNW-----TNLA------- 777

gi_154496732_ref_ZP_02035 749 AWALSWV-EEAE-------QTYEGNPHEV-----------------GLHMEHGKNGRDWSRFP------- 786

gi_189219502_ref_YP_00194 826 LRIVQPV-FFLK-------SKYLERVSGPD----------SKRARMELHIEGDG-----QLLP------- 865

gi_121606151_ref_YP_98348 822 LRYLKPL-KFLE-------EKYRRRIDTG-----------GARARLELHTEGDG-----NAWP------- 860

gi_75908069_ref_YP_322365 830 LRLISSL-ERMR-------NPYLREQNS---------------ATSFLHNDYQ------VAFP------- 863

gi_193212596_ref_YP_00199 888 IPSIKSL-DQWY-------VSYRSE------------------DKIPVHTHRIT-----STFVHARELTL 926

gi_149916637_ref_ZP_01905 899 LYCFPHINEDMK-------AAYRRFQGQR-------------EPAWPLHIDHHW-----ERLS------- 936

gi_88602022_ref_YP_502200 677 I-------EDVK--------EYNIRKAE------------------------------------------ 689

gi_153807774_ref_ZP_01960 743 AYCITPL-PSYE-------EKYNNCNA-------------------CCHFDAIIRNKMLREEY------- 778

[Consensus_aa:](http://prodata.swmed.edu/promals3d/info/consensus.html) ...*h*.s*h*...*h*p.......p.**Y**.p.....................................*h*........

[Consensus_ss:](http://prodata.swmed.edu/promals3d/info/consensus_ss.html) hhhhhhh hhhh hhhhhhh

Conservation: 5

gi_160892492_ref_ZP_02073 ----------------------------------------------------------------------

gi_163815161_ref_ZP_02206 ----------------------------------------------------------------------

gi_153954291_ref_YP_00139 842 SPI---------PEQSWGTTYVNERVKNYNDEVREL---------------------------------- 868

gi_226315300_ref_YP_00277 840 SPI---------PEKSWGDTYQNGRIKAYNAKVREL---------------------------------- 866

gi_219848227_ref_YP_00246 761 DLV------------------------------------------------------------------- 763

gi_22298564_ref_NP_681811 912 DVF---------PEN---------------PQILNL---------------------------------- 923

gi_254410688_ref_ZP_05024 901 DVF---------PED---------------PAIFSL---------------------------------- 912

gi_284051667_ref_ZP_06381 902 DFH---------RPD---------------PTIEQL---------------------------------- 913

gi_37520551_ref_NP_923928 840 DLM---------PSTE------------DEVQVRRN---------------------------------- 854

gi_15805565_ref_NP_294261 840 DWT---------PIND-----------AEIRKLQEAE--------------------------------- 856

gi_238059621_ref_ZP_04604 903 HVI---------AE--------------EKNAAVLEAYSE------------------------------ 919

gi_240169446_ref_ZP_04748 933 ASL---------PMGD-----------AERRTMTAG---------------------------------- 948

gi_227497172_ref_ZP_03927 953 GGL---------PMGD-----------DDRRAMVHG---------------------------------- 968

gi_269956178_ref_YP_00332 916 EFV---------PVST-----------TVLMSMVKG---------------------------------- 931

gi_268325165_emb_CBH38753 680 -------------------------------DLLTG---------------------------------- 684

gi_83312057_ref_YP_422321 921 ------------GMGAAD------DHEVQRSMIYHAFRG------------------------------- 941

gi_17229535_ref_NP_486083 778 NLI---------PPEDD---------GGAL---------------------------------------- 789

gi_154496732_ref_ZP_02035 787 NLFNQSLFHGAVPKWDMR------EAALAKEAVEDLKRADELGLEVRTNPDDAGATAEYAIYLPNPGTSA 850

gi_189219502_ref_YP_00194 866 SLF---------VPSRS----------EMEERIAPA---------------------------------- 882

gi_121606151_ref_YP_98348 861 RLF---------VASSA----------EVKLQALPY---------------------------------- 877

gi_75908069_ref_YP_322365 864 DII---------PPDA-----------IAMEKLEDV---------------------------------- 879

gi_193212596_ref_YP_00199 927 DEL---------ANR---------------AEDFKL---------------------------------- 938

gi_149916637_ref_ZP_01905 937 DLD---------PEDRRA-------KLEAVEAQRRV---------------------------------- 956

gi_88602022_ref_YP_502200 ----------------------------------------------------------------------

gi_153807774_ref_ZP_01960 779 NLY---------PK-------------AAIDDTLEL---------------------------------- 792

[Consensus_aa:](http://prodata.swmed.edu/promals3d/info/consensus.html) s.*h*.........s...................*h*.p...................................

[Consensus_ss:](http://prodata.swmed.edu/promals3d/info/consensus_ss.html) hhhhhhhh

Conservation:

gi_160892492_ref_ZP_02073 ----------------------------------------------------------------------

gi_163815161_ref_ZP_02206 ----------------------------------------------------------------------

gi_153954291_ref_YP_00139 ----------------------------------------------------------------------

gi_226315300_ref_YP_00277 ----------------------------------------------------------------------

gi_219848227_ref_YP_00246 ----------------------------------------------------------------------

gi_22298564_ref_NP_681811 ----------------------------------------------------------------------

gi_254410688_ref_ZP_05024 ----------------------------------------------------------------------

gi_284051667_ref_ZP_06381 ----------------------------------------------------------------------

gi_37520551_ref_NP_923928 ----------------------------------------------------------------------

gi_15805565_ref_NP_294261 ----------------------------------------------------------------------

gi_238059621_ref_ZP_04604 ----------------------------------------------------------------------

gi_240169446_ref_ZP_04748 ----------------------------------------------------------------------

gi_227497172_ref_ZP_03927 ----------------------------------------------------------------------

gi_269956178_ref_YP_00332 ----------------------------------------------------------------------

gi_268325165_emb_CBH38753 ----------------------------------------------------------------------

gi_83312057_ref_YP_422321 ----------------------------------------------------------------------

gi_17229535_ref_NP_486083 ----------------------------------------------------------------------

gi_154496732_ref_ZP_02035 851 GDLLAAAQLDKDKTYTMAELCAILADKQVPDSEGFPKPAMVREDLKYVNQVMTTTDIPAPADLGERLAER 920

gi_189219502_ref_YP_00194 ----------------------------------------------------------------------

gi_121606151_ref_YP_98348 ----------------------------------------------------------------------

gi_75908069_ref_YP_322365 ----------------------------------------------------------------------

gi_193212596_ref_YP_00199 ----------------------------------------------------------------------

gi_149916637_ref_ZP_01905 ----------------------------------------------------------------------

gi_88602022_ref_YP_502200 ----------------------------------------------------------------------

gi_153807774_ref_ZP_01960 ----------------------------------------------------------------------

[Consensus_aa:](http://prodata.swmed.edu/promals3d/info/consensus.html) ......................................................................

[Consensus_ss:](http://prodata.swmed.edu/promals3d/info/consensus_ss.html)

Conservation:

gi_160892492_ref_ZP_02073 702 -----------------------MLSVAFSDKKL------------------------------------ 712

gi_163815161_ref_ZP_02206 702 -----------------------MFTVAFSDNKL------------------------------------ 712

gi_153954291_ref_YP_00139 869 ----------------------FKRGVELGCVIDTEKGSNER------YKCIITKDFNIDEFMEQY---- 906

gi_226315300_ref_YP_00277 867 ----------------------FDRAVRYGAIRE--KGSDSQTSN--RYESIVTKSFSLAAFLAKH---- 906

gi_219848227_ref_YP_00246 ----------------------------------------------------------------------

gi_22298564_ref_NP_681811 924 ----------------------VVIARALGVLKQAENRATRE----------------ATIRYTRH---- 951

gi_254410688_ref_ZP_05024 913 ----------------------VVQARALGVLRQEENRTTKE----------------TVIRYTRQ---- 940

gi_284051667_ref_ZP_06381 914 ----------------------VLIARAFEILRQEINKKTKQ----------------DVIRYRIM---- 941

gi_37520551_ref_NP_923928 855 ----------------------AILGRALDLIDLVENKLTGYQ--------------EARLRFFDR---- 884

gi_15805565_ref_NP_294261 857 --------------------QLVFLSALHGNLQL--TGGHL-------------------VMEWPQ---- 881

gi_238059621_ref_ZP_04604 920 -----------------KLVGDGVLARSLGRLG------------------------------------- 935

gi_240169446_ref_ZP_04748 949 ----------------------WLLGQIIGRIQI---PESPYI---------------EPVRVYDG---- 974

gi_227497172_ref_ZP_03927 969 ----------------------WWVAFFSGGIQR---PGWGME------KDT------DPISVWDA---- 997

gi_269956178_ref_YP_00332 932 ----------------------WFVARRLGHLDL--TPATAR----------------IWVPETNT---- 957

gi_268325165_emb_CBH38753 685 ----------------------MDIAENISNLKV---PDSFS---------------------------- 701

gi_83312057_ref_YP_422321 942 ------------------EAEAWFIERQFARERQ------------------------TDIQGADQ---- 965

gi_17229535_ref_NP_486083 790 --------------------RWFALALAPNPYSLIVNE---------------------RKQYFVH---- 814

gi_154496732_ref_ZP_02035 921 GLRRKMAAWTALKAAFPVVEELKALLEAHNTAAGLQEKAAQ------------------RSQTFLK---- 968

gi_189219502_ref_YP_00194 883 ----------------------LLLGMVLKEKDDGGNERELVQK-----CQTDTGG--FQLGFMRK---- 919

gi_121606151_ref_YP_98348 878 ----------------------VLLAKALGFIHEGRNPATGA----------------DEVLLLTK---- 905

gi_75908069_ref_YP_322365 880 ----------------------FYPCLAFRLLKE--NQENQQ----------------LEFQYYDS---- 905

gi_193212596_ref_YP_00199 939 ----------------------FVLAVALGVLRRTENGADAGLYSVSKKGRTQAIGDEKKLRLMGIPDAY 986

gi_149916637_ref_ZP_01905 957 ----------------------SVVGLALGLARGTVEFGD----------QAVKGGPGYVLRVRED---- 990

gi_88602022_ref_YP_502200 690 -----------------------YVQNKLKEKTD------------------------------------ 700

gi_153807774_ref_ZP_01960 793 ----------------------WVKGFIFGLIKN---END--------------------KYYYQS---- 813

[Consensus_aa:](http://prodata.swmed.edu/promals3d/info/consensus.html) ......................*hhh*t..*h*s.*h*p.............................*hh*......

[Consensus_ss:](http://prodata.swmed.edu/promals3d/info/consensus_ss.html) hhhhhhhhhh

Conservation:

gi_160892492_ref_ZP_02073 713 ------AENDIN-----------------------------------------VMFGSPTN--------- 726

gi_163815161_ref_ZP_02206 713 ------TEQDIN-----------------------------------------VMFGSPVN--------- 726

gi_153954291_ref_YP_00139 907 -----SIDMQEDTPNIGE--------------IKRA----------ITDIEK-LLKEGITPVENHDMQKY 946

gi_226315300_ref_YP_00277 907 -----QAQGDATKLSPGE--------------IKRA----------LVELKG-FMREGLEQEFTRDVFGS 946

gi_219848227_ref_YP_00246 ----------------------------------------------------------------------

gi_22298564_ref_NP_681811 952 -----TAVGLEDVDI-------------------AAN---------WEEVTQ-VLEV------------- 974

gi_254410688_ref_ZP_05024 941 -----TAIGAESVDI-------------------ASS---------WEEASQ-VLEV------------- 963

gi_284051667_ref_ZP_06381 942 -----TKLGEDKVTV-------------------AAN---------WEDTVQ-VLQL------------- 964

gi_37520551_ref_NP_923928 885 -----QSGLEKTE------------------VL-GND---------WQSATE-FLLN------------- 907

gi_15805565_ref_NP_294261 882 -----QPG-------EPT--EPEKRARRF-----AGS---------FGKAAR-QLAFEP----------- 911

gi_238059621_ref_ZP_04604 936 ---------------------------GDLLPLCRDAD-------LLQAAAV-ALAN------------- 957

gi_240169446_ref_ZP_04748 975 -----DAEQWLSFPSPLL--TPPSNFTASYDWL-PAV---------LEGVLL-AIAQ------------- 1013

gi_227497172_ref_ZP_03927 998 -----DSERFVPFPAPML--TSHSQMVTPNSAL-AAV---------LESILL-AYLD------------- 1036

gi_269956178_ref_YP_00332 958 -----RAGYLADFPQVMLRSDPLNQSEG----L-AVV---------LESMML-ALVQ------------- 994

gi_268325165_emb_CBH38753 702 --------------------------IAGLTVL-VQG---------LENASR-SMEK------------- 721

gi_83312057_ref_YP_422321 966 ---------LIP--------PRIARLLGAPDFC-QAF---------IQCLAAQAIVR------------- 995

gi_17229535_ref_NP_486083 815 -----TD--------------QARKLEGGTLLL-GDD---------RKSAFK-AFKNNVS---------- 844

gi_154496732_ref_ZP_02035 969 ----SLAGGLIAYDDLRACWNMELGDEKP--LSGELESRLERDCKEYYAAQA-FYAL------------- 1018

gi_189219502_ref_YP_00194 920 -----DEYGFDQPV-----------------YLGGET---------FSETLA-NIRE------------- 944

gi_121606151_ref_YP_98348 906 -----DADGFDND------------PVA----L-GKS---------FMASAD-SIDL------------- 930

gi_75908069_ref_YP_322365 906 -----LRDSYNTA------------------TL-SPE---------WSQALE-ELANRNDM--------- 932

gi_193212596_ref_YP_00199 987 RSIITEQVSLDIEK--------------------LSS---------LEQLAM-LVALLDYYVNFVYPVAT 1026

gi_149916637_ref_ZP_01905 991 -----RTLP-----------------------L-GTS---------LLAAAD-ALIAL------------ 1009

gi_88602022_ref_YP_502200 701 -----IAGPI------------------------GTI--------------------------------- 708

gi_153807774_ref_ZP_01960 814 -----EEYGDP---------------------L-DDN---------WVELLK-YRDE------------- 833

[Consensus_aa:](http://prodata.swmed.edu/promals3d/info/consensus.html) .....p.............................s..........*h*..*h*....*h*...............

[Consensus_ss:](http://prodata.swmed.edu/promals3d/info/consensus_ss.html) hhh hhhhhh hhhh

Conservation:

gi_160892492_ref_ZP_02073 727 ------------DRRD--EVMQK-VAAAF--------VNDIQEEKQLCVLKDGYKELLLNKKYISVPNVM 773

gi_163815161_ref_ZP_02206 727 ------------DRRT--AIMEE-AAKAF--------VDDISEEQPLCVLKDDYKEKLLAKKYISVPNVM 773

gi_153954291_ref_YP_00139 947 YIFESSDEERAQNNLIRTPELTRLLLQEVEKY--EAIIAKKEELESTIEGLSK---------------AQ 999

gi_226315300_ref_YP_00277 947 --INEEMAKENLIRYP--EQIRL-IEQEVRKY--EEIEAKIQELEQIVGAIKD---------------EE 994

gi_219848227_ref_YP_00246 ----------------------------------------------------------------------

gi_22298564_ref_NP_681811 975 ------------LACRP-------DLEEIQR--QVTVILKA----------AETPA---QKQALY----- 1005

gi_254410688_ref_ZP_05024 964 ------------LACRP-------DREEIQR--QVMQKLNA----------AETPT---QKQAIF----- 994

gi_284051667_ref_ZP_06381 965 ------------PDCRE-------DRQEVER--QLNELLDQ----------AETES---QKQQLG----- 995

gi_37520551_ref_NP_923928 908 ------------DANRR-------LKDILAD--ELARQGGS----------PTTRP---AKQEFY----- 938

gi_15805565_ref_NP_294261 912 ------------QDSSR------PSKSLTNAATYLRSEIEARRKAIVGR--YPTPA---EGRQA------ 952

gi_238059621_ref_ZP_04604 958 ------------ERLSP-------ILAPDGG---------------DHVLGWTT-----RSQRGR---PH 985

gi_240169446_ref_ZP_04748 1014 ------------SQDPP-------VMRSLRPYQVLRGLYDA-NSQDPAGGIVQL-----SGVGL------ 1052

gi_227497172_ref_ZP_03927 1037 ------------VDDS---------LSVFTPWQVLRRWADTSDNAPSSKMGVRT-----PTQQA------ 1074

gi_269956178_ref_YP_00332 995 ------------VNTRQ-------SVDPINPYARLRTLGE----------GPTA-----FGNQLC----- 1025

gi_268325165_emb_CBH38753 722 ------------VGLWA--------KGAGKGFAQLKTEFEESIAK---LKGAEA-----PGSEKA----- 758

gi_83312057_ref_YP_422321 996 ------------DEES------------------LKWVWRIPDS--EQKVVLSRDGAT-LDDDFI----- 1027

gi_17229535_ref_NP_486083 845 ------------------------LVKEIAQ--KVDKITHQ------------------DKEKAR----- 865

gi_154496732_ref_ZP_02035 1019 ------------SDD---DYER--FTDALAE--QKETATDA------------------DLKAAR--EQR 1049

gi_189219502_ref_YP_00194 945 ------------AE-----------GLLLEE--KVALLLSN----------PEYKIES-QRKAAE----- 973

gi_121606151_ref_YP_98348 931 ------------AN-----------LHTIKS--VCNAMLAG----------AAYLHQD-RRTEVQ----- 959

gi_75908069_ref_YP_322365 933 ------------------TEA---LLQLLER--EISVISGQ----------PEL-----WENQYL----- 959

gi_193212596_ref_YP_00199 1027 L-RLDGTDVDKKQFPT--LICEK-LVEEWAG--RLEAKAGS----------ANAER---LRREAS----- 1072

gi_149916637_ref_ZP_01905 1010 ------------RKDMP---------AVYDS--WVAPLLAD----------VATLDD--ALRAEL----- 1039

gi_88602022_ref_YP_502200 709 --------------------------FAYPE--WFPKD-------------------------------- 718

gi_153807774_ref_ZP_01960 834 ----------------------------AFD--AFKR----------------------SKNSVR----- 846

[Consensus_aa:](http://prodata.swmed.edu/promals3d/info/consensus.html) ............................*h*.....*h*.................p........p.*h*......

[Consensus_ss:](http://prodata.swmed.edu/promals3d/info/consensus_ss.html) hhhhhh hhhhhh hhhhhh

Conservation:

gi_160892492_ref_ZP_02073 774 PYFSRAVKGILMDKP--------YNEKEDS-ITLNPG--------EIEISINDMYVGVPASMMA------ 820

gi_163815161_ref_ZP_02206 774 PYFSKAVKAILMGKP--------YNEKEDS-ITLNPG--------EIEISIDDIFVGVPVSMMQ------ 820

gi_153954291_ref_YP_00139 1000 ADMDNFLRALYTDTIVKKGVFYIYNGGDDSKVSIEPFINTLKEKEYHHEAVFKKYSSLDERDKVIIDVTS 1069

gi_226315300_ref_YP_00277 995 ELVTRFIEALYTGTICKRGALYVYDKDEEE-DAWEPFINLMKVNKHFEFAIYEKFRALDHKSMATIQRKA 1063

gi_219848227_ref_YP_00246 ----------------------------------------------------------------------

gi_22298564_ref_NP_681811 1006 EHLLNYLKQREEELHKAGGRD----------------SLEYKR-----------EAAILQGV-------- 1040

gi_254410688_ref_ZP_05024 995 GQLTRYLEQRALELDKQGGKD----------------SPDYKR-----------EARILLDV-------- 1029

gi_284051667_ref_ZP_06381 996 KQLQDFLAERLK-TEFRNNDE----------------ETMYLR-----------EKNIILEF-------- 1029

gi_37520551_ref_NP_923928 939 TRLMQWLAVHRA--EIEGGED----------------NPAYRE-----------AAEAIEDF-------- 971

gi_15805565_ref_NP_294261 953 --YVKWLHDNMSSSSARAVRDWSDQAAQTA--------LMAYLTQDADL-----RQALLETFPPDN---- 1003

gi_238059621_ref_ZP_04604 986 QEIGDEARLGL-----------------------ILRNLVTLSTRLAEIR----RDAVLAAA-------- 1020

gi_240169446_ref_ZP_04748 1053 --LRDFILNGWSTPDVVS----------------RIKAITAAET--PTDR----AMAAEEWL-------- 1090

gi_227497172_ref_ZP_03927 1075 --ISELLGHG-EVAGL------------------PTPAFLEGLTQPEER-----RQALATLC-------- 1110

gi_269956178_ref_YP_00332 1026 QELQDWVLLGANARSTVR----------------PTEATAEGR-----------QKEVVNDL-------- 1060

gi_268325165_emb_CBH38753 759 EKIVKMIDDSARNMEILTERIDDLSNRFGEDINLVLDRAMEFISGIPSEET---SSSVIRHI-------- 817

gi_83312057_ref_YP_422321 1028 SAAVTFILQRSEAKLGSRVT-----------IGLDAARTSASHWSARINPGK-PLHQVVADF-------- 1077

gi_17229535_ref_NP_486083 866 FSLESYI--------------------------------------------------------------- 872

gi_154496732_ref_ZP_02035 1050 EALRDYC--------------------------------------------------------------- 1056

gi_189219502_ref_YP_00194 974 SAIVKLVNGLRQ--SIPPT------------------DPRAKK-----------LPDWGKLA-------- 1004

gi_121606151_ref_YP_98348 960 RAILAEVEAVKA--ARGGNIQ----------------DETYRR-----------FLDAGRRA-------- 992

gi_75908069_ref_YP_322365 960 PKLRQFVQAVD-----DLSED----------------SPNYPY-----------KLAVVGTSASTDPTVK 997

gi_193212596_ref_YP_00199 1073 DKLNEWTAEL------PGSVK-----------------DVYEYE-------------------------- 1093

gi_149916637_ref_ZP_01905 1040 KQLTKSWAVRAQDLELDGKTR----------------SAEYRD--------------------------- 1066

gi_88602022_ref_YP_502200 719 --------------------------------------PLVQEV-------------------------- 724

gi_153807774_ref_ZP_01960 847 KEFNQYFEQFQKTK----------------------------------------GAEAMQAL-------- 868

[Consensus_aa:](http://prodata.swmed.edu/promals3d/info/consensus.html) ..*h*.p*hh*.................................*h*................*h*...*h*........

[Consensus_ss:](http://prodata.swmed.edu/promals3d/info/consensus_ss.html) hhhhhhhhhhh hhhhh hhhhhhhh

Conservation:

gi_160892492_ref_ZP_02073 821 ------------------CTAEMQKAY----NAVDSSYKG------------------------------ 838

gi_163815161_ref_ZP_02206 821 ------------------CAMEMQMAY----NAV-YDYKG------------------------------ 837

gi_153954291_ref_YP_00139 1070 DRRLENLSMEEDTKTLILNIDTLLETF----KECIRELDIRKYQLVNGDEL------------------- 1116

gi_226315300_ref_YP_00277 1064 AKRSDAMTSAEDTQQLLTKLEEIATAF----QEAKNDLEYDRDEYVNGEEL------------------- 1110

gi_219848227_ref_YP_00246 ----------------------------------------------------------------------

gi_22298564_ref_NP_681811 1041 ----IQTYQLAQGTSAP--------------SPMPTPPAPE------PPL--PQPE-------------- 1070

gi_254410688_ref_ZP_05024 1030 ----IERYKLKVESQSV-SPGVVQQPRSDGGN-APTQIEAK------SVT--PQAH-------------- 1071

gi_284051667_ref_ZP_06381 1030 ----IINHKLGTVNSPS--------------TPPPVITAPHLGFTTEPEDNQNKML-------------- 1067

gi_37520551_ref_NP_923928 972 ----IKTHNLYIEGTPRPGAAANGPARATPAPPPAAILQPGDPAR--ESNL-DKYRKLVESCVKDGELSA 1034

gi_15805565_ref_NP_294261 1004 -NLIDGLYKD--KGERLPKGRVANT---QGYYCRVCGGP------------------------------- 1036

gi_238059621_ref_ZP_04604 1021 ----DEARRD--MDWEYG---------TRRFYALPSA--------------------------------- 1042

gi_240169446_ref_ZP_04748 1091 ----ATVRD---TAAEYLPPGTSRAVNAGAFARIATRSKAS-KTP------------------------- 1127

gi_227497172_ref_ZP_03927 1111 ----DNILAD--LDQRYLPGEGKQ-DGPGSFTNFRSRELVD-SAP------------------------- 1147

gi_269956178_ref_YP_00332 1061 ----VAVRA---KYREY-------------FATIESQRKDPLDFP------------------------- 1085

gi_268325165_emb_CBH38753 818 ----TKAESEVSKIRET-------------SMKVVRGMKD------------------------------ 840

gi_83312057_ref_YP_422321 1078 ----VAPA----ALDKF-------------LATALPEPN--VGDP------------------------- 1099

gi_17229535_ref_NP_486083 873 ----KQVNE---LLKNNR-VGLPIK--------------------------------------------- 889

gi_154496732_ref_ZP_02035 1057 ----EKLRG---LKRADKELGISCAMASRDFEDKAG---------------------------------- 1085

gi_189219502_ref_YP_00194 1005 ----I----------------------------------------------------------------- 1005

gi_121606151_ref_YP_98348 993 ----V----------------------------------------------------------------- 993

gi_75908069_ref_YP_322365 998 EGIIHRFRRK---MNERFSISQ----SRAFAP-------NNNTSMQTAIAGEIVVDMP------------ 1041

gi_193212596_ref_YP_00199 1094 ----VNFREC---MAKKVLQLDVFDDGWSFTGRKQEAEPEIQVSKPEPIRPQLCFHVAVD---------G 1147

gi_149916637_ref_ZP_01905 ----------------------------------------------------------------------

gi_88602022_ref_YP_502200 ----------------------------------------------------------------------

gi_153807774_ref_ZP_01960 869 ----IDDAKLYYFDKYSQIS--------LTKEQIKAK--------------------------------- 893

[Consensus_aa:](http://prodata.swmed.edu/promals3d/info/consensus.html) ......................................................................

[Consensus_ss:](http://prodata.swmed.edu/promals3d/info/consensus_ss.html) hhhh

Conservation:

gi_160892492_ref_ZP_02073 839 ---------------LHIDEV-NRDMRDF--------------PNLCTL--------------------- 857

gi_163815161_ref_ZP_02206 838 ---------------LHIDEV-NKDMKSF--------------PDFVTI--------------------- 856

gi_153954291_ref_YP_00139 1117 ------FAFY-NEMLSRLQEV-RKQLEQ------------------------------------------ 1136

gi_226315300_ref_YP_00277 1111 ------FQFY-KKVWSKVNDM-RKTLQ------------------------------------------- 1129

gi_219848227_ref_YP_00246 ----------------------------------------------------------------------

gi_22298564_ref_NP_681811 1071 ---PTLETTA-AASSAGLEQL-QRLIAMYQ-----------QGLLSEVE-----------------FQAA 1107

gi_254410688_ref_ZP_05024 1072 ---VFCTNCG-TKNPSN----------------------------SKFC-----------------FKCG 1092

gi_284051667_ref_ZP_06381 1068 --GNVMANTGIKYCNKCGYSM-SISDKFC--------------SNCGN-------------------NMS 1101

gi_37520551_ref_NP_923928 1035 TEKTLLERFR-VRYSVSPEQS-QQLIELLTPKPQNKGAVLEYGLMFRAFLENDAAIDPEEQAQLLELQEE 1102

gi_15805565_ref_NP_294261 1037 ----VGETFE---DVIGQGLK-CEYHPDD--------------VEHPFG-QRYSVFG------------- 1070

gi_238059621_ref_ZP_04604 1043 -----------HRIEPELWHL-LTVAAHV-------------------AIDRRS---------------- 1065

gi_240169446_ref_ZP_04748 1128 ----IFRDLA-PDVFWAAEML-IKLVRQV--------------KATAV--DGKSPTAAVTFDEGEQVVIP 1175

gi_227497172_ref_ZP_03927 1148 ----LSIDLA-QEMYDELTSV-REVIASV--------------APAGAAATRRSPLEG------GMVY-- 1189

gi_269956178_ref_YP_00332 1086 ----PMWDLR-RLIDRALTEL-KHGVEAL--------------ETTTGD-TGWS---------------- 1118

gi_268325165_emb_CBH38753 841 -----MGEPL-AMILATLREL-KRVTETS-------------------EAEGTGEPAQV-----EAVTGK 879

gi_83312057_ref_YP_422321 1100 ----VKSTLH-RRNCAALGLI-FRFYGRP--------------GAQTAL-SSRNL--------------- 1133

gi_17229535_ref_NP_486083 890 -----------EQVEMEIQEI-DAYLEDL--------------DVIS----------------------- 910

gi_154496732_ref_ZP_02035 1086 -----------EELAAKIRRFYDWMIEEL----------------------------------------- 1103

gi_189219502_ref_YP_00194 1006 ----------------------KRIRGEI----------------------------------------- 1012

gi_121606151_ref_YP_98348 994 ----------------------AILKGEA--------------A-------------------------- 1001

gi_75908069_ref_YP_322365 1042 VDTTDNRVRR----RLELERL-KQDLDED--------------FITQDEYEREKQRIFA-----QYPL-- 1085

gi_193212596_ref_YP_00199 1148 HAAGPFME----------EKL-LQMISMG--------------QLT------ETTKVWKKGMPSWLTASE 1186

gi_149916637_ref_ZP_01905 1067 -----------------LAEA-RAILAPV--------------LD------------------------- 1079

gi_88602022_ref_YP_502200 725 ----------------------YLDIGSE--------------S-------------------------- 732

gi_153807774_ref_ZP_01960 894 ----GFESIR-KLITEELEYV-KKEL-------------------------------------------- 913

[Consensus_aa:](http://prodata.swmed.edu/promals3d/info/consensus.html) .................*h*..*h*....*h*............................................

[Consensus_ss:](http://prodata.swmed.edu/promals3d/info/consensus_ss.html) hhhh hhhhhhhhhh hhhhhh

Conservation:

gi_160892492_ref_ZP_02073 ----------------------

gi_163815161_ref_ZP_02206 ----------------------

gi_153954291_ref_YP_00139 ----------------------

gi_226315300_ref_YP_00277 ----------------------

gi_219848227_ref_YP_00246 ----------------------

gi_22298564_ref_NP_681811 1108 KKKLLGL--------------- 1114

gi_254410688_ref_ZP_05024 1093 TQLVKLN--------------- 1099

gi_284051667_ref_ZP_06381 ----------------------

gi_37520551_ref_NP_923928 1103 LGLTNDQVHIIEANVKEELGQS 1124

gi_15805565_ref_NP_294261 ----------------------

gi_238059621_ref_ZP_04604 ----------------------

gi_240169446_ref_ZP_04748 1176 DGGTF----------------- 1180

gi_227497172_ref_ZP_03927 ----------------------

gi_269956178_ref_YP_00332 ----------------------

gi_268325165_emb_CBH38753 880 DNGPELPDLSLSLEREEE---- 897

gi_83312057_ref_YP_422321 ----------------------

gi_17229535_ref_NP_486083 ----------------------

gi_154496732_ref_ZP_02035 ----------------------

gi_189219502_ref_YP_00194 ----------------------

gi_121606151_ref_YP_98348 ----------------------

gi_75908069_ref_YP_322365 ----------------------

gi_193212596_ref_YP_00199 1187 VPELSVFFETPPALNDGPPLLD 1208

gi_149916637_ref_ZP_01905 ----------------------

gi_88602022_ref_YP_502200 ----------------------

gi_153807774_ref_ZP_01960 ----------------------

[Consensus_aa:](http://prodata.swmed.edu/promals3d/info/consensus.html) ......................

[Consensus_ss:](http://prodata.swmed.edu/promals3d/info/consensus_ss.html)

**C: FtsZ-like 2**

Conservation: 9 6

gi_90409221_ref_ZP_012173 1 MEIKKSALKEKNHDSEAFNNTELEESKNNS-------------INEEKEISM----------NSQS--IL 45

gi_34499290_ref_NP_903505 1 MIM-QSMYD---------------QSSNPP-------------TT----------------PDEPTAAPP 25

gi_152997985_ref_YP_00134 ----------------------------------------------------------------------

gi_87119891_ref_ZP_010757 1 MMS------------------------------------------------------------------- 3

gi_226941044_ref_YP_00279 1 MS-----------------------TTSDTSVELLEP--VDKTLPSSKGAVI------------------ 27

gi_192360015_ref_YP_00198 1 MA-----------------------TRKNSRQTSTLDEGQQDDVALLDATEE------------------ 29

gi_146308213_ref_YP_00118 1 MK-----------------------TKKDV-------------VAPAKEATL--------ADIADN--LL 24

gi_109896728_ref_YP_65998 1 MT-----------------------TKTQM------------DLPAVDETTEQVIEKKSDAKKITS--ES 33

gi_77457477_ref_YP_346982 1 MT-----------------------SKNNP-------------ATAVSDLTL--------SPAANSPAPM 26

gi_70728682_ref_YP_258431 1 MT-----------------------SKNKP-------------ANAVSEMTL--------TSPTTSPSDP 26

[Consensus_aa:](http://prodata.swmed.edu/promals3d/info/consensus.html) **M**........................opp...............*h*s.........................

[Consensus_ss:](http://prodata.swmed.edu/promals3d/info/consensus_ss.html)

Conservation: 9769 6 99 776 66 7 99999

gi_90409221_ref_ZP_012173 46 DDHVP---------SSSK------HMYFIEDDTQRILENLTELKKQIYPLH--DNFSVEDQTNAPFPSVC 98

gi_34499290_ref_NP_903505 26 QAVTTR-----QLLNLNERGQHGLYQYFIEHDTNRILQHLDTLRGMAFSRD--AEDGQSGPTEKPFPSVC 88

gi_152997985_ref_YP_00134 1 ---------MSQENLPIE------HLYFTETNLEQILENLDVVRNRIHPQS-TDE---EGINEQVFPSVC 51

gi_87119891_ref_ZP_010757 4 -----------KDVALNE------HLYYTEENLDNILENLDIVRRQIFPNL-DPE---SEINDQNFPSVC 52

gi_226941044_ref_YP_00279 28 ----HGQL---QLEGSEA------FQYFTEKDFDRILRNLDGLRSMVFPLG-GTDMQDDANAEQPFPSVC 83

gi_192360015_ref_YP_00198 30 ---NPSVAEVVRSDEGPG------YMYFTERDLGKILFNLDNLRSSVYPHL-NLDQDSDNRKQPQFPSVC 89

gi_146308213_ref_YP_00118 25 APALPG-----RSDTGEQ------YLYFTERDIERILDNLDGLRNLVFPLG-EPLEEGESSRIQQFPSVC 82

gi_109896728_ref_YP_65998 34 APSPPSTS---DTLVTDP------YMYFTERDLDKILANIDALRNSVFPVI-NPDADIESRSPQNFPSVC 93

gi_77457477_ref_YP_346982 27 KPLPPS-----RPLGTQQ------YLYFTETNTDRILDNLDGLRDLVFPRPPHLEGDNEQHNDQEFPSVC 85

gi_70728682_ref_YP_258431 27 KPLSSS-----RPLSTQQ------YLYFTETNTDRILDNLDGLRDTVFPRPPHLEGEPDNRRDQEFPSVC 85

[Consensus_aa:](http://prodata.swmed.edu/promals3d/info/consensus.html) ...........p............*@*b**Y***@h***E**ps*h*pp**IL**.p**L**s.*l***+**..*h@*s.....p...p..p...**FPSVC**

[Consensus_ss:](http://prodata.swmed.edu/promals3d/info/consensus_ss.html) eeeehhhhhhhhhhhhhhhhhh ee

Conservation: 9999999999969969 999799 9 65 5 6 97 9 5669

gi_90409221_ref_ZP_012173 99 LIGLGRCGSNISLDLANLVYNARNYYLNDFQKSGAKT-----KN-QSSLTTQWIKNTLGIKDKHNKHAAF 162

gi_34499290_ref_NP_903505 89 LIGLGRCGSNIALDVATLVYNARKFYLNEFNQDDKSA-----PEQEQRRPRRWLHRNLLAQ-HKAAKPAF 152

gi_152997985_ref_YP_00134 52 LIGLGRCGSNIALDVASLVYDARANYMREVESQEILD-----SE-SEFRPMRWIRKHLPLEDKDGFKPVF 115

gi_87119891_ref_ZP_010757 53 LIGLGRCGSNIALDVATLVYNARSNYMADLELQDKAS-----SE-GEFRPMRWIQKHLPIEIKGEPRPVF 116

gi_226941044_ref_YP_00279 84 LIGLGRCGSNIALDVASLVYNARQFYSNEFEHEEASKS--PVVE-TERQPKNWIRRNLARTQSRVSKPVF 150

gi_192360015_ref_YP_00198 90 LIGLGRCGSNIALDVASLVYNARNFYLNEFLSEEKAI-----RE-QDQRPMRWIKRSLNIKSGRQLKPVF 153

gi_146308213_ref_YP_00118 83 LIGLGRCGSNIALDVASLVYNARQFYLEEFHSEATAA-----IE-QASRPSRWIRSNLLRAPHKSSKPVF 146

gi_109896728_ref_YP_65998 94 LIGLGRCGSNIALDVASLVYNARNFYLNEFENEERET-----RE-QEYRPMRWIKRGLHLSSAQDVKPVF 157

gi_77457477_ref_YP_346982 86 LIGLGRCGSNIALDVAELVYNARKFYLNEFNNEDRAADRRLASD-KGYSPAQWIKQNLRIGPNKSTKPVF 154

gi_70728682_ref_YP_258431 86 LIGLGRCGSNIALDVAELVYNARKFYLNEFNNEDK------AYE-GGYSPGQWIRQNLRLGQNKASKPVF 148

[Consensus_aa:](http://prodata.swmed.edu/promals3d/info/consensus.html) **LIGLGRCGSNI**t**LD***l***A**p**LVY**s**AR**..**Y***h*.**-***h*p.p..........p.....s.p**W***l*ppp**L**........**+**s*h***F**

[Consensus_ss:](http://prodata.swmed.edu/promals3d/info/consensus_ss.html) eeee hhhhhhhhhhhhhhhhhhhhhhh hhhhhhh ee

Conservation: 9699 9 99999699 996569 6 66 9 9669999799999969999699999999999966

gi_90409221_ref_ZP_012173 163 LIEPFVILGDLDQDISGRVHYSYMDGKHSLLDGYPKMKIMDLSEVHAGGSGNAPVLGQYLAKIILNKGAE 232

gi_34499290_ref_NP_903505 153 LIEPMVMLGDLDKDIKGRIRFSRRGEQGDFINNYNKLKIMDLAEVHAGGAGNAPILGQYLAKIILNKDTL 222

gi_152997985_ref_YP_00134 116 LIEPIVLLGDLDKDIEGRIRFSNQEGRTKFLEEYTKLQIMDLSEVHAGGAGNAPILGQYLAKIILNKDAT 185

gi_87119891_ref_ZP_010757 117 LIEPVVMLGDLDKDIEGRIRFSSQNGRSNFLDEYKKLQIMDLSEVHAGGAGNAPILGQYLAKIILNKDTN 186

gi_226941044_ref_YP_00279 151 LIEPMVMLGDLDKDIKGRILFSRRGGDGSFLSDYNKMKIMDLSEVHAGGAGNAPILGQYLAKIILNKDTQ 220

gi_192360015_ref_YP_00198 154 LIEPLVMLGDLDKDIDGRIRFSHKGERSNFLKDYTKMKIMDLSEVHAGGSGNAPILGQYLAKIILNKDTQ 223

gi_146308213_ref_YP_00118 147 LIEPLVMLGDLDKDIAGRIRFSRKGEMSGFLDDYSKMKIMDLSEVHAGGAGNAPILGQYLAKIILNKDTQ 216

gi_109896728_ref_YP_65998 158 LIEPLVMLGDLDKDIEGRVRFSNKGEKASFLQEYTKMKIMDLSEVHAGGAGNAPILGQYLAKIILNKDTQ 227

gi_77457477_ref_YP_346982 155 LVEPLVMLGDLDKDIAGRIRFSRKGEKSGFLRDYSKMKIMDLSEVHAGGAGNAPILGQYLAKIILNKDTQ 224

gi_70728682_ref_YP_258431 149 LVEPLVMLGDLDKDIAGRIRFSRKGEKSGFIRDYSKMKIMDLSEVHAGGAGNAPILGQYLAKIILNKDTQ 218

[Consensus_aa:](http://prodata.swmed.edu/promals3d/info/consensus.html) **L***l***EP***h***V***h***LGDLD**p**DI**.**GR***l***+***@***S**....p..*hl*p.**Y**.**K***h*p**IMDL**t**EVHAGG**t**GNAP***l***LGQYLAKIILNK**s*h*.

[Consensus_ss:](http://prodata.swmed.edu/promals3d/info/consensus_ss.html) ee hhhhh eeeee hh eeeeehhhhh hhhhhhhhhhh hh

Conservation: 9 5 595 99996999999999999696999999999999999999999 6976996

gi_90409221_ref_ZP_012173 233 KFLNKDWKSIHSYLIDSCGIKANQSRLFFYIFSAGGGTGSGMASEFGLAQQYAYMSKTLHTEKKIQSERR 302

gi_34499290_ref_NP_903505 223 SFSDPDWTLIHSYLVDSCGIKANQSRLYFYIFSAGGGTGSGMASEFGLAQQYAYMSKTFETRNEGKADQ- 291

gi_152997985_ref_YP_00134 186 TFRNENWKQMHSYLVDSCGIKANQSRLYFYIFSAGGGTGSGMASEFGLAQQFSYLSKTFDYRSDQSQMA- 254

gi_87119891_ref_ZP_010757 187 SFNNSAWRHMHSYLVDSCGIKANQSRLYFYIFSAGGGTGSGMASEFGLAQQYSYMSKTFDYHSAQSKVL- 255

gi_226941044_ref_YP_00279 221 RFSNADWKFIHSYLIDSCGIKANQSRLYFYIFSAGGGTGSGMASEFGLAQQYSYMSKTFDTRDESQIDD- 289

gi_192360015_ref_YP_00198 224 RFSNPDWKFVHSYLIDSCGIKANQSRLYFYIFSAGGGTGSGMASEFGLAQQYAYMSKTFETKSLNDNSA- 292

gi_146308213_ref_YP_00118 217 RFSNEDWKFIHSYLIDSCGIKANQSRLYFYIFSAGGGTGSGMASEFGLAQQFAYMSKTFDSKAPEDGEA- 285

gi_109896728_ref_YP_65998 228 RFANKDWKYIHSYLIDSCGIKANQSRLYFYIFSAGGGTGSGMASEFGLAQQYAYMSKTFETRAVNESED- 296

gi_77457477_ref_YP_346982 225 RFSSPDWKMIHSYLIDSCGIKANQSRLYFSIFSAGGGTGSGMASEFGLAQQHSYMNKTFDTKPMDEHDG- 293

gi_70728682_ref_YP_258431 219 RFSSPDWKMIHSYLIDSCGIKANQSRLYFSIFSAGGGTGSGMASEFGLAQQYSYMNKTFDTKPMDEHDS- 287

[Consensus_aa:](http://prodata.swmed.edu/promals3d/info/consensus.html) p**F**.s.s**W**p.*h***HSYL***l***DSCGIKANQSRL***@***F**.**IFSAGGGTGSGMASEFGLAQQ***@*t**Y***h*s**KT***h*c*h*c...p....

[Consensus_ss:](http://prodata.swmed.edu/promals3d/info/consensus_ss.html) h hhhhhhhhhhhhh eeeeeeee hhhhhhhhhhhhhhhh

Conservation: 6999999969999699996 7 7999999999997995699979999 9 56 96

gi_90409221_ref_ZP_012173 303 SDAHSFVFEPIFSSGICILPNIACQSVEISEALHINAGRLLCKYLSEEWDFSYNVDREDHESDRTIVDRL 372

gi_34499290_ref_NP_903505 292 GDDYGFVFEPIFTSGICILPNISGQHAEGSEALHINAGRLLCKYLSEEWDFSYNFDNEEAGA-ESVMRRI 360

gi_152997985_ref_YP_00134 255 DKRHSFVFEPIFTSGICILPNISGKNVEISEALHINAGRLLTKYISEEWNFSYNEEREDDEVPADVMERI 324

gi_87119891_ref_ZP_010757 256 DKRHSFVFEPIFTSGICILPNISGDKIEVSEALHINAGRLLCKYLSEEWDFSYNVEREDHDHPSNVMQRI 325

gi_226941044_ref_YP_00279 290 GSGRSFVFEPIFTSGICILPNISDSTSSGAEALHINAGRLLCKYLAEEWDFSYNFDNEEANT-ESVMHRI 358

gi_192360015_ref_YP_00198 293 NHGHSFVFEPIFTSGICILPNISDHGVEMSEALHINAGRLLCKYLSEEWDFSYNFDNEDSSD-ASVMHRI 361

gi_146308213_ref_YP_00118 286 ERDRGFVFEPIFTSGICILPNISDQRSEMSEALHINAGRLLCKYLAEEWDFSYNFDNEQSSA-ESVMRRI 354

gi_109896728_ref_YP_65998 297 NRGHSFVFEPIFTSGICILPNISDHGVEMSEALHINAGRLLCKYMAEEWDFSYNFDKEDSSD-ASAMHRI 365

gi_77457477_ref_YP_346982 294 KSGHSFVFEPIFTSGICVLPNISDHRSEMSEALHINAGRLLCKYLSEEWDFSYNFANEDSSE-ASVMGRI 362

gi_70728682_ref_YP_258431 288 KSGHSFVFEPIFTSGICVLPNISDHRSEMSEALHINAGRLLCKYLSEEWDFSYNFDNEDSSE-ASVMGRI 356

[Consensus_aa:](http://prodata.swmed.edu/promals3d/info/consensus.html) .p..t**FVFEPIF**o**SGIC***l***LPNI**tsp..**E**.**SEALHINAGRLL***h***KY***l*t**EEW**s**FSYN**.**-**p**E-**.....s*lh*.**R***l*

[Consensus_ss:](http://prodata.swmed.edu/promals3d/info/consensus_ss.html) eeeeeeee eee eeee hhhhhhhhhhh ee hhhhhh

Conservation: 9999699999999999995 5997699669999999999999999999 99966997799697

gi_90409221_ref_ZP_012173 373 RPWNSMMLISNDIMRYAEEADGGSMENVDVNTMEKYANQYISQQIFNILTAQAVTKDYDQDYFRRAGVDI 442

gi_34499290_ref_NP_903505 361 RPWNAMMLISNDIMRYAEESEGGNIEHLDVNAMERHANQYISQQIFNILTAQAVTSDYDENYFLRAGIDM 430

gi_152997985_ref_YP_00134 325 RPWNSMMLISNDIMRYAEEENGGDSDNIDVNTMEKYANQYISQQIFNILTAQAVTTDYDEDYFRRAGIDI 394

gi_87119891_ref_ZP_010757 326 RPWNSMMLISNDIMRYAEENDGAE-SDIDVNTMEKYANQYISQQIFNILTAQAVTTDYDEDYFRRAGVDI 394

gi_226941044_ref_YP_00279 359 RPWNAMMLISNDIMRYAESDQDSGIGHIDVNAMERHANQYISQQIFNILTAQAVTTDYDQNYFRNAGIDI 428

gi_192360015_ref_YP_00198 362 RPWNAMMLISNDIMRYAEATDEGNIPHIDVNAMEKYANQYISQQIFNILTAQAVTTDYDENYFRRAGIDI 431

gi_146308213_ref_YP_00118 355 RPWNAMMLISNDIMRYAEESGDGSIHNIDVTAMERHANQYISQQIFNILTAQAVTSDYDQNYFRRAGIDI 424

gi_109896728_ref_YP_65998 366 RPWNAMMLISNDIMRYAEETDDSGIQYIDVNAMEKHANQYISQQIFNILTAQAVTTDYDENYFRRAGIDI 435

gi_77457477_ref_YP_346982 363 RPWNAMMLISNDIMRYAEESDDGNIQNIDVNAMEKHANQYISQQIFNILTAQAVTTDYDQNYFRRAGIDI 432

gi_70728682_ref_YP_258431 357 RPWNAMMLISNDIMRYAEESDDGNIQNIDVNAMEKHANQYISQQIFNILTAQAVTTDYDQNYFRRAGIDI 426

[Consensus_aa:](http://prodata.swmed.edu/promals3d/info/consensus.html) **RPWN**t**MMLISNDIMRYAE**p.pst...p*l***DVN***h***ME+***@***ANQYISQQIFNILTAQAVT**p**DYD**ps**YF**.**RAG***l***D***h*

[Consensus_ss:](http://prodata.swmed.edu/promals3d/info/consensus_ss.html) hhhhhhhhhhhhhh eeeeehhhhhhhhhhhhhhhhhhhhhhhh hhhhhhhh h

**T7**

Conservation: 6999999999999999996 9999967 5699999 999699999 9

gi_90409221_ref_ZP_012173 443 SDTIRLDANDLFMSLAGPVTIAYAESTVKKTAPII--NGRQED--SELVELDIDDLFFRSIDLPHFNKDT 508

gi_34499290_ref_NP_903505 431 GETIRLDANDLFMSLAGPVAVAYAESVVSDPLG--------SD--GFVNKLDIDDLFYRSIDLPHFNQQT 490

gi_152997985_ref_YP_00134 395 AETIRLDANDLFMSLAGPVAVAYAESVVNEGT--------------RQDSVDIDDLFCRSIELPHFNNDT 450

gi_87119891_ref_ZP_010757 395 AETIRLDANDLFMSLAGPVAIAYAESVVTSST---------------KENIDIDDLFCRSIDLPHFNQDT 449

gi_226941044_ref_YP_00279 429 GETIRLDANDLFMSLAGPVAVAYAESVVSDPL--------ALP--EHNGGLDIDDLFFRSIELPHFNRTT 488

gi_192360015_ref_YP_00198 432 GETIRLDANDLFMSLAGPVAVAYAESVIPALPQ-NSEKSRPQD--KKSSELNIDDLFYRSIDLPHFNKVT 498

gi_146308213_ref_YP_00118 425 GETIRLDANDLFMSLAGPVAVAYAESVVPEQHAQLSEKFRVLDKEQHPPRLNIDDLFFRSIDLPHFNKVT 494

gi_109896728_ref_YP_65998 436 GETIRLDANDLFMSLAGPVALAYAESVVPVENVSAVDKIKILD--RGANDLDIDDLFFRSIDLPHFNKVT 503

gi_77457477_ref_YP_346982 433 GETIRLDANDLFMSLAGPVAIAYAESVVPETPPPSSDKFKVFD--KEPQRLNIDDLFFRSIDLPHFNKVT 500

gi_70728682_ref_YP_258431 427 GETIRLDANDLFMSLAGPVAIAYAESVVPEQPVPTGDKFKVFE--KEPQRLNIDDLFFRSIDLPHFNKVT 494

[Consensus_aa:](http://prodata.swmed.edu/promals3d/info/consensus.html) t**-TIRLDANDLFMSLAGPV***hl***AYAES***h***V**..............s......p*l*s**IDDLF***h***RSI-LPHFN**p.**T**

[Consensus_ss:](http://prodata.swmed.edu/promals3d/info/consensus_ss.html) hheeee hhhhhhh hhhhhhhhh hhhhhh h

Conservation: 999969599 66 97 7 6 6577 9 6 99569996959 9997699976 699799 999

gi_90409221_ref_ZP_012173 509 RAIEGVSLLPMKAEIYREHLAECRDNNYDTSKLKEILFFNKCSSVVSIISLPKDYKLSYMDLNRLKTHLN 578

gi_34499290_ref_NP_903505 491 QAIEGISLLPIESASYRAALADYKQSGYAAEKLNQLFFFQNCSSVVAILSLPKGYKLSYLDLNRLKRHLN 560

gi_152997985_ref_YP_00134 451 SAIEGVSVLPVESDRYREAIKNYRKSGYDATALNHLHFFKNCSSIVSIISLPRDYKLSFTALNRLKMHLN 520

gi_87119891_ref_ZP_010757 450 SAIEGISILPIESSSYSEALKNYRESGYDTNSLNHLHFFKNCSSVVSIISLPKDYKLSFTALNRLKLHLN 519

gi_226941044_ref_YP_00279 489 QAIEGISLLPIESQRYRAALDQYRQSGFDAEQLRELFFFKNCSSIVTIVSLPKGYKLAYTDLNRLKTHLN 558

gi_192360015_ref_YP_00198 499 QAIEGISLLPIESDRYRAALGRYLQNSYDPEELKQLHFFKNCSSIVSIVSLPKDYKLSYMDLNKLKSHLN 568

gi_146308213_ref_YP_00118 495 QAIEGISLLPIESKRYRQALEQYKASGYDATQLSDLHFFKNCSSVVSIVSLPKDYKLSYMDLNRLKTHLN 564

gi_109896728_ref_YP_65998 504 QAIEGISLLPIESTRYRERLSEYVKSGYDATHLKDLHFFKNCSSVVSIISLPKDYKLSYMDLNRLKSHLN 573

gi_77457477_ref_YP_346982 501 QAIEGISLLPIESKRYRASLEQYKNSGYDAAALHDLHFFKNCSSVVSIVSLPKDYKLSYMDLNRLKTHLN 570

gi_70728682_ref_YP_258431 495 QAIEGISLLPIESKRYRAALEQYKASGYDAAALHDLHFFKNCSSVVSIVSLPKDYKLSYMDLNRLKTHLN 564

[Consensus_aa:](http://prodata.swmed.edu/promals3d/info/consensus.html) p**AIEG***l***S***l***LP***h*ct..**Y**p..*l*.p*h*..ss**Y**s*h*..**L**pp*lh***FF**pp**CSS***l***V**t**I***l***SLP+**s**YKLS***@h*s**LNRLK**.**HLN**

[Consensus_ss:](http://prodata.swmed.edu/promals3d/info/consensus_ss.html) hhh eee hhhhhhhhhhhhh hhhhhhhhhh hhhhhhhh eehhhhhhhhhhhhh

Conservation: 9797999999999999959699999669999999999996966 99999 76656 9 7 59

gi_90409221_ref_ZP_012173 579 SLFPNTTLKRYALVIGASENISLTTLIAKSPCLSDDFLTLIVSYVKRCFAKNEYCFDDNFDDAFLNFITS 648

gi_34499290_ref_NP_903505 561 DLFPSTTLKRYALVIGASTNLSLTTLVAKSPCLSDDFLTLIVAYIKRCFAHGNHRFDDSLDNVILDLITA 630

gi_152997985_ref_YP_00134 521 RLCPNTTLKRYALVIGASANLSLTTLIAKSPCLSDDFLTLMVAYMKRCFARDDYRYTDEIDKAIIKMIQD 590

gi_87119891_ref_ZP_010757 520 SLFPNTTLKRYALVIGASANLSLTTLVAKSPCLSDDFLTLMVAYVKRCFARDEYRFSDEVDKAILNFITT 589

gi_226941044_ref_YP_00279 559 NLFPNTTLKRYALVIGASANLSLTTLVVKSPCLSDDFLTLIVAFIKRCFAVDQYRYDESLDKEILDFITS 628

gi_192360015_ref_YP_00198 569 ALFPNTTLKRYALVIGASANISLTTLIVKSPCLSDDFLTLIVAFIKRCFAKSDYRFDEQLDKAMLEFISA 638

gi_146308213_ref_YP_00118 565 NLFPNTTLKRYALVIGASANLSLTTLIVKSPCLSDDFLTLIVAYIKRCFARDQYRFDDSLDDAMLDFIVA 634

gi_109896728_ref_YP_65998 574 ALFPNTTLKRYALVIGASANISLTTLVVKSPCLSDDFLTLIVAYIKRCFAKDNYRFDEQLDQAMLDFIRG 643

gi_77457477_ref_YP_346982 571 SLFPNTTLKRYALVIGASANLSLTTLIAKSPCLSDDFLTLIVAFIKRCFAKTPYRFDETLDNSILDFIIQ 640

gi_70728682_ref_YP_258431 565 SLFPNTTLKRYALVIGASANLSLTTLIAKSPCLSDDFLTLIVAFIKRCFARNPYRFDDTLDNSVLDFIVS 634

[Consensus_aa:](http://prodata.swmed.edu/promals3d/info/consensus.html) .**L***h***P**s**TTLKRYALVIGAS**.**N***l***SLTTL***lh***KSPCLSDDFLTL***h***V**t*@h***KRCFA+**s.*@*.*@*s**-**p*h***D**ps*hl*p*h***I**.s

[Consensus_ss:](http://prodata.swmed.edu/promals3d/info/consensus_ss.html) hh eeeeeeee eeeeee hhhhhhhhhhhhhhhh hhhhhhhhhhhh

Conservation: 6 967 7 97999999999599999999999966 6959999999 77 7599999

gi_90409221_ref_ZP_012173 649 DDFDEAGLDKMISEFENPAKILDTNWHAIKPMYEKKYREILGDEEKFISINDIRLTSTNVKRAIKYLREI 718

gi_34499290_ref_NP_903505 631 DTFDETVLERLLNEYENPAKILDTNWFAIKPMYEKKYRELIRDSKRFVSINDIRLTREHVQKAVCYLREI 700

gi_152997985_ref_YP_00134 591 DSFDDALIDKFFTTYENPAKILDTNWYAIKPMYEKKYRELIDNNQRFVSINDIRLDINNVKNAIKYLREI 660

gi_87119891_ref_ZP_010757 590 EEFNEEEVDRYFNTHENPAKILDTNWYAIKPMYEKKYRELIDDQEKFVSINDIRLDVENVKKSIKYLREI 659

gi_226941044_ref_YP_00279 629 EHFDESRLDGMLEPHENPAKILDTNWYAIKPMYEKKYRELIHNPDKFVSINDIRLSRDSIKKTIRYLREI 698

gi_192360015_ref_YP_00198 639 DQFDEAFVDEMLNEYENPAKILDTNWYAIKPMYEKKYRELINDPEKFCSINDIRLSRTSVKKAIKYLREI 708

gi_146308213_ref_YP_00118 635 ERFNEAQLDAMLNEHEDPAKILDTNWYAIKPMYEKKYRELIHDAEKFVSINDIRLTRDSVKQAIKYLREI 704

gi_109896728_ref_YP_65998 644 ETFDEDALDAMLEEHENPAKILDTNWYAIKPMYEKKYRELIREKDKFVSINDIRLSRDSVKKAIKYLREI 713

gi_77457477_ref_YP_346982 641 EEFDEDRIDDLLNEFENPAKILDTNWYAIKPMYEKKYRELINDKEKFVSINDIRLSRDCVKKSIKYLREI 710

gi_70728682_ref_YP_258431 635 DQFDEDRIDDLLNEFENPAKILDTNWYAIKPMYEKKYRELINDKDKFVSINDIRLSRDCVKKAIKYLREI 704

[Consensus_aa:](http://prodata.swmed.edu/promals3d/info/consensus.html) **-**p**F**s**-**..*l***-**.*hh*pp*@***ENPAKILDTNW***@***AIKPMYEKKYRE***ll*.s.p**+F***l***SINDIRL**s.p.**V**ppt*l*.**YLREI**

[Consensus_ss:](http://prodata.swmed.edu/promals3d/info/consensus_ss.html) hhhhhhhhhh hhhhh hhhhhhhhhh hhheeee eee hhhhhhhhhhhhhh

Conservation: 99666699 799

gi_90409221_ref_ZP_012173 719 YRYRVSKTKLISLNGKKKTSK------ 739

gi_34499290_ref_NP_903505 701 YRHRIGKTRVVSLNSYSRKHHA----- 722

gi_152997985_ref_YP_00134 661 YRHRISKTNVLTLNG------------ 675

gi_87119891_ref_ZP_010757 660 YRHRVSKTNVLSLNF------------ 674

gi_226941044_ref_YP_00279 699 YRHRVGKTKIVSLNEYVADQARHSA-- 723

gi_192360015_ref_YP_00198 709 YRHKISKTRIVSLNADFPGSLEPARKS 735

gi_146308213_ref_YP_00118 705 YRHRIGKTRVISLNDYGK--------- 722

gi_109896728_ref_YP_65998 714 YRHKISKTKVISLNDDSSGM------- 733

gi_77457477_ref_YP_346982 711 YRHRIGKTKVISLNNHTGKSYSV---- 733

gi_70728682_ref_YP_258431 705 YRHRIGKTRVISLNSHTGKTAY----- 726

[Consensus_aa:](http://prodata.swmed.edu/promals3d/info/consensus.html) **YR***@***+***l*t**KT**p*ll*o**LN**.............

[Consensus_ss:](http://prodata.swmed.edu/promals3d/info/consensus_ss.html) hhhh eeeeee
