# Supplementary material for: Two new families of the FtsZ-tubulin protein superfamily implicated in membrane remodeling in diverse bacteria and archaea
Source: Biol Direct. 2010 May 7;5:33. doi: 10.1186/1745-6150-5-33 (PMC2875224; doi:10.1186/1745-6150-5-33)
Supplement: Additional file 6 — Uncharacterized putative components of the predicted FtsZl1-centered membrane remodeling system. The data represents the list and comments for gene families associated with FtsZl1 genes in archaeal and bacterial genomes. [file 1745-6150-5-33-S6.DOCX]

**Uncharacterized putative components of the predicted FtsZl1-centered membrane remodeling system (presented in the Figure 2C)**

| Family | Representative | Comment |
| --- | --- | --- |
| **Archaea** | | |
| A1 | MA2713 | Mixed α and β |
| A2 | MA2710 | Membrane-associated protein |
| GTPase | MA2712 | Membrane associated GTPase |
| **Bacteria** | | |
| vWA | CKL_1663 | Von Willebrand factor type A (vWA) domain containing proteins, often membrane associated. Belong to COG2304. |
| OmpA-like | DR_0536 | Membrane-associated protein with distinct β-stranded transmembrane domains. |
| GTPase | cgR_2621 | Diverged GTPase often fused to Zn-binding domains |
| B1 | SGR_3832 | Membrane-associated protein |
| B2 | cgR_2628 | Membrane-associated protein |
| B3 | cgR_2625 | Large membrane-associated protein |
| B4 | cgR_2624 | Large protein with fibronectin type II domain, often associated with S/T protein kinase domain |
| B5 | cgR_2623 | Large protein with low-complexity regions |
| B6 | cgR_2622 | Protein with low-complexity regions |
| B7 | Ava_1849 | Membrane-associated protein |
| B8 | CKL_1665 | Large protein with low-complexity region |
| B9 | CKL_1664 | - |
